# Proceedings of the Canadian Society of Allergy and Clinical Immunology Annual Scientific Meeting 2021

**DOI:** 10.1186/s13223-022-00647-5

**Published:** 2022-03-22

## Allergic Rhinitis/Asthma

### 01 Investigating the prevalence, accuracy of self-reporting, and mental health impacts of allergic disease in health care professional students during the COVID-19 pandemic

#### Alyssa G. Burrows^1,2^, Sydney Joy^1,2,3^, Sarah Garvey^1^, Sophia Linton^1,2^, Jenny Thiele^1,2^, Lisa M. Steacy^2^, Dean A. Tripp^4^, Anne K. Ellis^1,2,3^

##### ^1^Department of Medicine, Queen’s University, Kingston, ON, Canada, ^2^Allergy Research Unit, Kingston, ON, Canada, ^3^Department of Biomedical Medical Sciences, Queen’s University,, Kingston, ON, Canada, ^4^Department of Psychology, Anesthesia, Urology, Queen’s University,, Kingston, ON, Canada

###### **Correspondance:** Alyssa G. Burrows

*Allergy, Asthma & Clinical Immunology* 2022, **17(Suppl 1)**: 01

**Background:** COVID-19 symptoms overlap with allergic rhinitis (AR) and asthma, potentially impacting mental well-being [1]. Research regarding the effects of anxiety and stress on Health Care Professional (HCP) students throughout the COVID-19 pandemic is beginning to emerge. It is currently unknown if trainees with atopic conditions experience different stress levels than their non-atopic peers. In Canadian adults, the estimated prevalence for AR and food allergy(FA) is 44% and 6.1%, respectively [2,3].

**Methods:** Between August 2020 to June 2021, Faculty of Health Sciences students (n = 266) completed a one-time questionnaire using the QualtricsXM^TM^survey platform of which 114 respondents disclosed their atopic status. The following data was collected: Self-reported atopy status, Generalized Anxiety Disorder-7(GAD-7), Patient Health Questionaire-9(PHQ-9), and Perceived Stress Score-10(PSS-10). Participants were then classified based on the type and number of atopic conditions they reported. A follow-up visit involving skin prick testing (SPT) to a standard panel of 9 aeroallergen and food extracts, and/or fresh fruits, where applicable, was completed (n = 34) to determine how accurately allergies were self-reported. Statistical analyses were performed using SPSS 27.

**Results:** Having a self-reported allergic condition or asthma did not impact GAD-7, PSS, and PHQ-9 scores, in HCP students. Further stratifying the dataset by the type and number of allergic conditions also did not impact GAD-7, PSS, and PHQ-9 scores or severity. The self-reported prevalence of asthma, AR and FA was 5.71%, 64.71%, and 29.41%, respectively. SPT confirmed 64.71% and 8.82% of participants were sensitized to AR and food allergens, respectively. Generally, seasonal AR allergies were underreported whereas, perennial AR and FA were overreported.

**Conclusions:** Atopic conditions did not impact mental health scores in HCP student’s which suggests that they are generally aware of their atopic conditions and able to differentiate allergy and COVID-19 symptoms. Self-reported accuracy varied and may be impacted by the specific allergic condition.

**References:**

1. Shaker MS, Oppenheimer J, Grayson M, Stukus D, Hartog N, Hsieh EW, Rider N, Dutmer CM, Vander Leek TK, Kim H, Chan ES. COVID-19: pandemic contingency planning for the allergy and immunology clinic. The Journal of Allergy and Clinical Immunology: In Practice. 2020 May 1;8(5):1477–88.
2. Keith PK, Desrosiers M, Laister T, Schellenberg RR, Waserman S. The burden of allergic rhinitis (AR) in Canada: perspectives of physicians and patients. Allergy, Asthma & Clinical Immunology. 2012 Dec;8(1):1–1.
3. Clarke AE, Elliott SJ, Pierre YS, Soller L, La Vieille S, Ben-Shoshan M. Temporal trends in prevalence of food allergy in Canada. The Journal of Allergy and Clinical Immunology: In Practice. 2020 Apr 1;8(4):1428–30.

### 02 Triple therapy (LAMA, ICS and LABA) in asthma control in patients with uncontrolled, persistent asthma: a systematic review and meta-analysis

#### Anna Whalen-Browne, Lisa Kim, Carol Saleh, Paul O’Byrne, Derek Chu

##### McMaster University, Hamilton, ON, Canada

###### Correpondance: Anna Whalen-Browne

*Allergy, Asthma & Clinical Immunology* 2022, **17(Suppl 1)**: 02

**Background:** Among patients with moderate-severe asthma, benefits and harms of adding long-acting muscarinic antagonists (LAMA) to inhaled corticosteroids (ICS) and long-acting bronchodilators (LABA) remains unclear due to lack of systematic reviews and meta-analyses. The latest asthma recommendations comment on only one LAMA and include fewer than five studies from before 2017. The objective of this study was to systematically synthesize the efficacy and safety of triple (ICS-LABA-LAMA) vs. dual therapy (ICS-LABA) in persistent, uncontrolled asthma.

**Methods:** MEDLINE, EMBASE, CENTRAL, ICTRP, FDA and EMA databases from November 2017 to December 8, 2020 were searched. Two investigators independently selected randomized controlled trials (RCTs) comparing triple and dual therapy in moderate-severe asthma. Two reviewers independently extracted data and assessed risk of bias. Data was analysed using random-effects meta-analyses, including individual patient-level exacerbation data. GRADE approach was used to assess certainty. The primary outcome was severe exacerbations (risk ratio [RR], incidence rate ratio [IRR], hazard ratio [HR]). Secondary outcomes included asthma control, quality of life, FEV_1_, and adverse events. PROSPERO number CRD42020172608.

**Results:** Twenty RCTs that enrolled 11,894 patients and used three LAMA types were included. High certainty evidence revealed that compared with dual therapy, triple therapy decreased the number of severe asthma exacerbations (RR 0.83, 95% CI 0.77 to 0.90), and improved asthma control (SMD -0.06, 95% CI − 0.10 to − 0.02; MD in ACQ-7 scale − 0.04, 95% CI − 0.07 to − 0.01). There were no significant differences in asthma-related quality of life (SMD 0.05, 95% CI − 0.03 to 0.13; MD in AQLQ scale 0.05, 95% CI − 0.03 to 0.13) or mortality (RR 0.96, 95% CI 0.33 to 2.75). Adverse events were similar between groups.

**Conclusions:** In moderate-severe asthma, triple therapy safely reduces severe exacerbations with modest improvements in other patient-important outcomes. This supports add-on LAMA in patients at high risk for future exacerbation.

### 03 Serum biomarkers and Staphylococcus aureus carriage in ragweed-induced allergic rhinitis using the nasal allergen challenge model

#### Sophia Linton^1,2^, Jenny Thiele^1,2^, Lisa M. Steacy^2^, Lubnaa Hossenbaccus^1,2^, Anne K. Ellis^1,2^

##### ^1^Department of Medicine, Queen’s University, Kingston, ON, Canada, ^2^Kingston Allergy Research, Kingston Health Sciences Center - KGH Site, Kingston, ON, Canada

###### **Correspondance:** Sophia Linton

*Allergy, Asthma & Clinical Immunology* 2022, **17(Suppl 1)**: 03

**Background:** The relationship between *Staphylococcus aureus*(SA) nasal carriage and allergic rhinitis blood biomarkers is not known. The current study compares ragweed(RW) specific(s) immunoglobulin-E(IgE) levels and serum cytokine levels and in RW-allergic participants colonized with and without SA. The allergen exposure was modeled using the nasal allergen challenge model(NAC), which consists of a screening and challenge visit.

**Methods:** Nasal SA carriage was assessed using culture-based screening methods in 15 RW-allergic participants before their NAC with individualized RW-extract doses determined using a titration challenge. Peripheral blood samples collected at the screening visit were used to evaluate sIgE levels, while those collected at the challenge visit were used to evaluate serum cytokine(baseline, 6- and 24-hour post-NAC). The cytokines IL-1b, IL-4, IL-5, IL-6, IL-10, IL-13, TNF-a, IFN-g, MCP-1, MIP-1b, and RANTES were measured using Luminex xMAP^TM^ technology, and sIgE concentrations were measured using an ImmunoCAP assay on the Phadia^®^ 100 machine. All statistical analyses were performed on GraphPad Prism 9.0.

**Results:** Four RW-allergic participants were colonized with antibiotic-sensitive SA (SA^+^), and 11 were not(SA^-^). The sIgE (p = 0.3429) levels were not significantly different between the two populations. The concentration of MCP-1 at 24-hours was significantly higher in SA^+^ than SA^-^ (p = 0.0399). MIP1-b (p = 0.0170) and IFN-g (p = 0.0214) concentrations were significantly elevated in SA^-^ at 24-hours compared to baseline. Individual cytokine levels were widely distributed, and therefore the change in cytokine levels from baseline was calculated. The change in IL-5 concentration at 6-hours compared to baseline was significantly greater in SA^+^ than SA^-^(p = 0.0148).

**Conclusions:** There is differential cytokine expression in the blood of SA^+^ and SA^-^ populations post-NAC while sIgE levels were not significantly different. These results suggest that SA carriage may impact the allergic response to RW exposure in the peripheral blood, however more investigations are needed to support these findings and identify potential mechanistic pathways.

### 04 The role of gut microbiota in mediating allergic asthma in infants

#### John Christy Johnson, Peter Anto Johnson, Austin Mardon

##### University of Alberta, Edmonton, AB, Canada

###### **Correspondance:** John Christy Johnson

*Allergy, Asthma & Clinical Immunology* 2022, **17(Suppl 1)**: 04

**Background:** Allergic asthma in infants represents an increasingly common chronic disease, classically associated with hyperactivation of the T helper 2 (Th2) arm of adaptive immunity [1]. Recent culture-independent molecular detection technology presents growing evidence in rodent models that reveal correlations between allergic asthma with gut microbiota [1,2]. However, the mechanism of gut microbial strains in inducing chronic inflammation-related changes in infants with allergic asthma are still poorly understood [2]. Here, we conducted a narrative review to synthesize current evidence in the literature.

**Methods:** We conducted a review of the literature followed by a qualitative narrative synthesis following ENTREQ guidelines. Databases including PubMed/MEDLINE, EMBASE and Google Scholar were screened, and no time, setting, or language restrictions were imposed on the search strategy. Keywords in our search included: “allergic asthma”, “dysbiosis”, “gut microbiota”, “prebiotic” or “probiotic,” “Th2”, “adaptive immunity” and “IgE”. Primary research articles such as case studies, systematic reviews and meta-analyses, were included. Technology-based, animal, and non-infant studies were excluded.

**Results:** We identified 25 articles that met our inclusion criteria. Of the 25, fourteen discussed the role of specific microbial species that were associated with either pathogenic or protective roles in allergic asthma in infants. Four were systematic reviews identifying 346 cases cumulatively and evaluating outcomes and discussing risk factors. Three major mechanisms, including cesarean birth, formula feeding, and early-life exposure to antimicrobials accumulated the greatest mentions in literature. Maternal factors such as exposure to livestock/pets and antimicrobial use during pregnancy were further characterized or mentioned by 8 studies.

**Conclusions:** Evidence appears to suggest gut microbial flora is likely influenced by nutrition, medications, birth conditions, maternal factors, and environmental exposures. Numerous retrospective studies, case studies, and reviews and therapeutic applications of early prebiotic/probiotic therapy corroborate these mechanisms [3], but do not exclude other plausible manners in which gut dysbiosis induces and/or exacerbates allergic asthma.

**References:**

1. Di Gangi A, Di Cicco ME, Comberiati P, Peroni DG. Go With Your Gut: The Shaping of T-Cell Response by Gut Microbiota in Allergic Asthma. Front Immunol. 2020 Jul 14;11:1485. doi: 10.3389/fimmu.2020.01485. PMID: 32760404; PMCID: PMC7372123.
2. Fujimura KE, Lynch SV. Microbiota in allergy and asthma and the emerging relationship with the gut microbiome. Cell Host Microbe. 2015 May 13;17(5):592–602. doi: 10.1016/j.chom.2015.04.007. PMID: 25974301; PMCID: PMC4443817.
3. Kang YB, Cai Y, Zhang H. Gut microbiota and allergy/asthma: From pathogenesis to new therapeutic strategies. Allergol Immunopathol (Madr). 2017 May-Jun;45(3):305–309. doi: 10.1016/j.aller.2016.08.004. Epub 2016 Oct 28. PMID: 28029408.

### 05 The 12-SQ HDM SLIT-tablet shows similar safety and efficacy across geographies, ethnic and age groups

#### Hendrik Nolte^1^, Tomokazu Matsuoka^2^, David I. Bernstein^3^, Yuriko Maekawa^4^, Veronica Hulstrøm^5^

##### ^1^ALK, Bedminster, NJ, USA, ^2^Department of Otorhinolaryngology, Head & Neck Surgery, Faculty of Medicine, Graduate Faculty of Interdisciplinary Research, University of Yamanashi, Yamanashi, Japan, ^3^Bernstein Clinical Research Center and Division of Immunology, Allergy and Rheumatology, University of Cincinnati, Cincinnati, OH, USA, ^4^Torii Pharmaceuticals Co., Ltd., Tokyo, Japan, ^5^ALK, Hørsholm, Denmark

###### **Correspondance:** Hendrik Nolte

*Allergy, Asthma & Clinical Immunology* 2022, **17(Suppl 1)**: 05

**Background:** House dust mite (HDM) sublingual immunotherapy (SLIT)-tablets have been evaluated in large clinical trials of adolescents and adults with allergic rhinoconjunctivitis in North America and Japan. Because of the diversity of participants in the trials and the large sample sizes, it is possible to assess safety and efficacy across age groups and world regions.

**Methods:** Efficacy and safety data from 2 randomized, double-blind, placebo-controlled phase III clinical trials (NCT01700192 and JapicCTI-121848) with 12-SQ HDM were analysed by age (12–17 years/18–64 years) and ethnicity/region (Japan/North America [NA]). Trials were designed similarly with respect to medical practice, target population, eligibility criteria, efficacy and safety monitoring.

**Results:** The treatment effect on the primary endpoint of total combined rhinitis score (TCRS) in Japanese (N = 633) and NA (N = 1482) subjects were comparable both for the overall trial populations and the adolescent and adult subgroups, ranging between 16–22% relative to placebo. HDM SLIT-tablet was well tolerated, and in general, the safety profile was similar in Japanese and NA populations. The placebo-subtracted treatment-related adverse event (AE) rate in NA adolescents/adults was 45%/41% and in Japanese adolescents/adults was 47%/46%. There was no epinephrine use due to treatment-related events in adolescent subjects in either trial. Only 4 events of epinephrine use due to treatment-related events were reported among NA adult subjects. The HDM IgE and IgG_4_ responses were comparable between Japanese and NA subjects and between age groups.

**Conclusions:** In conclusion, the results show that SQ HDM SLIT-tablet is insensitive to ethnic, age or regional differences with a similar safety, efficacy, and immunologic profile.

### 06 Dupilumab provides early and durable improvement of symptoms in patients with chronic rhinosinusitis with nasal polyps: Results from the SINUS trials

#### Philippe Gevaert^1^, Stella E. Lee^2^, Russell Settipane^3^, Martin Wagenmann^4^, Jérôme Msihid^5^, Shahid Siddiqui^6^, Scott Nash^6^, Juby A. Jacob-Nara^7^, Asif H. Khan^8^, Siddhesh Kamat^6^, Chien-Chia Chuang^9^

##### ^1^Department of Otorhinolaryngology, Ghent University, Ghent, Belgium, ^2^Department of Otolaryngology—Head & Neck Surgery, University of Pittsburgh Medical Center, Pittsburgh, PA, USA, ^3^Department of Medicine, The Warren Alpert Medical School of Brown University, Providence, RI, USA, ^4^Department of Otorhinolaryngology, Düsseldorf University Hospital (UKD), Düsseldorf, Germany, ^5^Health Economics and Value Assessment, Sanofi, Chilly-Mazarin, France, ^6^Medical Affairs, Regeneron Pharmaceuticals, Inc., Tarrytown, NY, USA, ^7^Global Medical Affairs, Sanofi, Bridgewater, NJ, USA, ^8^Global Medical Affairs, Sanofi, Chilly-Mazarin, France, ^9^Health Economics and Value Assessment, Sanofi, Cambridge, MA, USA

###### **Correspondance:** Philippe Gevaert

*Allergy, Asthma & Clinical Immunology* 2022, **17(Suppl 1)**: 06

**Background:** Chronic rhinosinusitis with nasal polyps (CRSwNP) is a type 2 inflammatory disease, with cardinal symptoms of nasal congestion (NC), loss of smell (LoS), and rhinorrhea significantly impacting patients’ daily lives. We report dupilumab’s effect on patient-reported CRSwNP symptoms from the SINUS-24 (NCT02912468) and SINUS-52 (NCT02898454) studies.

**Methods:** Post hoc analysis in CRSwNP patients receiving dupilumab 300 mg or placebo q2w. Pooled SINUS-24/-52 intent-to-treat (ITT) population (N = 724) was used for assessment to Week 24 and SINUS-52 population (N = 303) for Week 52 assessment. Patients reported symptom scores (0 = none; 1 = mild; 2 = moderate; 3 = severe) daily for NC, LoS, and rhinorrhea. Proportions of patients with baseline scores ≥2 (moderate/severe) achieving improvement (score ≤1; none/mild) at Weeks 2, 24, and 52 were reported for the ITT population and subgroups with asthma/prior sinonasal surgery.

**Results:** At baseline, 86.7%, 94.1%, and 64.1% of patients had scores ≥2 for NC, LoS, and rhinorrhea, respectively. Significantly more patients achieved improvement (score ≤1; none/mild symptoms) with dupilumab vs placebo at Weeks 2/24/52 (dupilumab vs placebo, NC: 11.5%/54.2%/54.6% vs 1.6%/13.7%/15.9%; LoS, 5.1%/43.4%/43.8% vs 1.1%/5.5%/4.2%; rhinorrhea, 9.4%/53.2%/58.3% vs 2.2%/15.6%/20.4%). Results were consistent across subgroups at Weeks 24 and 52. Among patients not achieving scores ≤1 at Week 24, more dupilumab- vs placebo-treated patients experienced symptom improvement (scores of > 1–≤ 2).

**Conclusions:** In CRSwNP patients with moderate-to-severe symptom burden at baseline, dupilumab significantly improved patient-reported symptoms vs placebo. Symptom improvement was observed as early as Week 2, and continued to Week 52, suggesting early onset, durable treatment effect of dupilumab.

### 07 Time-varying effects of allergy on the childhood asthma risk: a retrospective cohort study

#### Rui Li^1^, Arthur H. Owora^1,3^, Robert S. Tepper^2^, Clare D. Ramsey^4^, Moira Chan-Yeung^5^, Wade T. Watson^6^, Allan B. Becker^3^

##### ^1^Department of Epidemiology and Biostatistics, School of Public Health, Indiana University Bloomington, Bloomington, IN, USA, ^2^Indiana University School of Medicine, Indianapolis, IN, USA, ^3^Children’s Hospital Research Institute of Manitoba, Department of Pediatrics and Child Health, University of Manitoba, Winnipeg, MB, Canada, ^4^Department of Internal Medicine, Max Rady College of Medicine, University of Manitoba, Winnipeg, MB, Canada, ^5^Department of Medicine, University of British Columbia, Vancouver, BC, Canada, ^6^Department of Pediatrics, Dalhousie University, Halifax, NS, Canada

###### **Correspondance:** Rui Li

*Allergy, Asthma & Clinical Immunology* 2022, **17(Suppl 1)**: 07

**Background:** The relationship between childhood allergies and asthma is heterogeneous and time-dependent[1]; however, there is a paucity of research on how different allergy patterns impact asthma risk over time. Our study objective was to examine the effect of different allergy patterns on asthma risk from birth to adolescence.

**Methods:** Secondary analysis of the Canadian Asthma Primary Prevention Study (CAPPS), a multifaceted prenatal intervention among children at high risk of asthma, followed from birth to 15 years was performed. Asthma and allergy diagnoses were based on a pediatric allergist’s clinical decision and skin prick test results, respectively. Marginal Structural Modeling (MSM) was used to examine whether asthma risk differs among children with different allergy histories at ages 1, 2, 7 and 15 years. Transition models were used to quantify the lagged effect of prior allergies at 7 and 15 years.

**Results:** On average, the odds of asthma were higher among children who had at least one positive allergy skin test result in the 1^st^ year (adjusted odds ratio [aOR]: 1.47; 95% CI: 1.15, 1.86); this association persisted for different skin test reactivity patterns over time. Irrespective of a child’s allergy history, the odds of asthma were lower among children randomized to the CAPPS intervention (aOR: 0.56; 95% CI: 0.42, 0.75). The effect of prior allergies on future diagnoses decreased over time (at age 7 years OR: 2.39; 95% CI: 2.00, 2.86 and at 15 years OR:1.46; 95% CI: 1.18, 1.81).

**Conclusions:** The magnitude and persistence of allergy effects on asthma risk during childhood into adolescence suggests greater efforts should be devoted to early allergy screening and preventive intervention.

**References:**

1. Dick, S., et al., *A systematic review of associations between environmental exposures and development of asthma in children aged up to 9 years.* BMJ Open, 2014. 4(11): p. e006554.

### 08 Modeling multi-state childhood allergy-asthma transitions: a retrospective cohort study

#### Arthur H. Owora^1^, Robert H. Tepper^2^, Clare H. Ramsey^3^, Moira H. Chan-Yeung^4^, Wade H. Watson^5^, Allan H. Becker^6^

##### ^1^Indiana University School of Public Health, Bloomington, IN, USA, ^2^Indiana University School of Medicine, Indianapolis, IN, USA, ^3^Department of Internal Medicine, Max Rady College of Medicine, University of Manitoba, Winnipeg, MB, Canada, ^4^Department of Medicine, University of British Columbia, Vancouver, BC, Canada, ^5^Department of Pediatrics, Dalhousie University, Halifax, NS, Canada, ^6^Children’s Hospital Research Institute of Manitoba, Department of Pediatrics and Child Health, University of Manitoba, Winnipeg, MB, Canada

###### **Correspondance:** Arthur H. Owora

*Allergy, Asthma & Clinical Immunology* 2022, **17(Suppl 1)**: 08

**Background:** Two-thirds of childhood asthma diagnoses especially severe cases have allergic origins[1, 2]. Prior to an asthma diagnosis, children may experience a time-varying burden of allergies as they grow older. Modeling these transitions can provide insights regarding disease pathogenesis and prognostic factors that influence disease progression. Our study objective was to characterize the natural course of allergy-mediated childhood asthma.

**Methods:** We carried out a secondary analysis of the Canadian Asthma Primary Prevention Study (CAPPS), a multifaceted prenatal intervention among children at high risk of asthma, followed from birth to 15 years. Asthma and allergy diagnoses were based on a pediatric allergist’s clinical decision and positive skin test results to food or non-food allergens, respectively. A staged Markov model was used to estimate the distribution, mean time to asthma diagnosis, and transition ratios between allergy-asthma states for 493 children.

**Results:** Our transition model had four bi-directional stages: I) no allergies/asthma, II) one allergy/no asthma, III) ≥ 2 allergies/no asthma, and IV) current asthma diagnosis; the average (standard error) duration in each stage was 15(2.9), 3(0.4), 11(2.4) and 8(2.3) years, respectively. The mean time to an asthma diagnosis was 10.2 years (95%CI: 1.7, 11.0). Adjusted for CAPPS intervention, transition to an asthma diagnosis was more likely from stage II (Transition Ratio [TR]: 3.4; 95%CI: 1.4, 8.0) and III (TR: 2.8; 95%CI: 1.5, 5.4) than Stage I. Recovery (i.e., reverse association) was more likely than disease progression from Stage II (TR 3.6; 95%CI: 1.92, 11.04) but less likely from Stage III (TR: 0.16; 95%CI: 0.1, 0.5) or IV (TR: 0.33; 95%CI: 0.1, 0.8).

**Conclusions:** Among children at high-genetic risk, allergy-mediated asthma risk appears to be reversible before but not after multiple sensitizations to aeroallergens. Our findings support prioritization of prevention efforts that mitigate early sensitization.

**References:**

1. Holt, P.G., et al., *The role of allergy in the development of asthma.* Nature, 1999. 402(6760 Suppl): p. B12–7.
2. *GINA Report 2020; Global Strategy of Asthma Management and Prevention. *

### 09 Long-term impact of dupilumab on the reduction of oral corticosteroid (OCS) use in patients with OCS-dependent asthma

#### Lawrence D. Sher^1^, Michael E. Wechsler^2^, Klaus F. Rabe^3,4^, Jorge F. Maspero^5^, Nadia Daizadeh^6^, Xuezhou Mao^7^, Benjamin Ortiz^8^, Leda P. Mannent^9^, Elizabeth Laws^7^, Nami Pandit-Abid^7^, David J. Lederer^8^, Megan Hardin^6^

##### ^1^Peninsula Research Associates, Rolling Hills Estates, CA, USA, ^2^National Jewish Health, Denver, CO, USA, ^3^LungenClinic Grosshansdorf (member of the German Center for Lung Research [DZL]), Airway Research Center North (ARCN), Grosshansdorf, Germany, ^4^Christian-Albrechts University (member of the German Center for Lung Research [DZL]), Airway Research Center North (ARCN), Kiel, Germany, ^5^Fundación CIDEA, Buenos Aires, Argentina, ^6^Sanofi, Cambridge, MA, USA, ^7^Sanofi, Bridgewater, NJ, USA, ^8^Regeneron Pharmaceuticals, Inc., Tarrytown, NY, USA, ^9^Sanofi, Chilly-Mazarin, France

###### **Correspondance:** Lawrence D. Sher

*Allergy, Asthma & Clinical Immunology* 2022, **17(Suppl 1)**: 09

**Background:** Patients with severe asthma may require long-term oral corticosteroids (OCS) treatment to control disease. Dupilumab, a fully human monoclonal antibody, blocks the shared receptor component for interleukin-4/interleukin-13, key/central drivers of type 2 inflammation in multiple diseases. In VENTURE (NCT02528214), dupilumab 300mg vs placebo reduced OCS dose and exacerbations and improved lung function in patients with OCS-dependent severe asthma. TRAVERSE, a single-arm, open-label extension study (NCT02134028), evaluated long-term safety, tolerability, and efficacy of dupilumab. We evaluated long-term maintenance of OCS reduction and clinical efficacy in VENTURE patients enrolled in TRAVERSE.

**Methods:** LS mean percentage change from parent study baseline (PSBL) in OCS use and percentage of patients with OCS reduction ≥50%, annualized exacerbation rate, LS mean change from PSBL in lung function (FEV_1_), and mean change from PSBL in asthma control (ACQ-5) at TRAVERSE Week48 were evaluated in VENTURE patients receiving dupilumab (dupilumab/dupilumab) or placebo (placebo/dupilumab) enrolled in TRAVERSE.

**Results:** VENTURE patients treated with dupilumab enrolled in TRAVERSE reduced LS mean OCS dose by 68.8% from PSBL to end of VENTURE. LS mean OCS dose was reduced by 78.3% (dupilumab/dupilumab) and 53.4% (placebo/dupilumab) from PSBL to TRAVERSE Week48. 74/90 dupilumab and 52/97 placebo patients achieved ≥50% reduction in OCS dose by end of VENTURE. Of evaluable patients, 47/49 (dupilumab/dupilumab) and 37/39 (placebo/dupilumab) sustained OCS reduction at TRAVERSE Week48. Unadjusted annualized exacerbation rates over TRAVERSE Weeks 0–48 were 0.442 (dupilumab/dupilumab) and 0.321 (placebo/dupilumab). FEV_1_ (L) increased (LS mean change [SE]: 0.25 [0.06] and 0.25 [0.05]) and ACQ-5 improved (mean change [SD]: – 1.06 [1.25] and – 1.21 [1.00]) from PSBL to TRAVERSE Week48 in dupilumab/dupilumab and placebo/dupilumab, respectively.

**Conclusions:** Patients with severe OCS-dependent asthma demonstrated sustained OCS dose reduction and clinical outcomes improvement during TRAVERSE. The OCS-sparing effect, exacerbation reduction, and lung function and asthma control improvements of dupilumab observed in VENTURE were maintained in TRAVERSE.

### 10 Impact of severe exacerbations on lung function in patients with uncontrolled, moderate-to-severe asthma treated with dupilumab: LIBERTY ASTHMA QUEST

#### Kenneth R. Chapman^1^, Alberto Papi^2^, Mario Castro^3^, Daniel J. Jackson^4^, Nadia Daizadeh^5^, Nami Pandit-Abid^6^, Yamo Deniz^7^, Paul J. Rowe^6^, Benjamin Ortiz^7^

##### ^1^University of Toronto, Toronto, ON, Canada, ^2^Respiratory Medicine Unit, University of Ferrara, S. Anna University Hospital, Ferrara, Italy, ^3^University of Kansas School of Medicine, Kansas City, KS, USA, ^4^University of Wisconsin School of Medicine and Public Health, Madison, WI, USA, ^5^Sanofi, Cambridge, MA, USA, ^6^Sanofi, Bridgewater, NJ, USA, ^7^Regeneron Pharmaceuticals, Inc., Tarrytown, NY, USA

###### **Correspondance:** Kenneth R. Chapman

*Allergy, Asthma & Clinical Immunology* 2022, **17(Suppl 1)**: 10

**Background:** Severe asthma exacerbations are associated with lung function impairment. Dupilumab, a fully human monoclonal antibody, blocks the shared receptor component for interleukin-4/interleukin-13, key and central drivers of type 2 (T2) inflammation in multiple diseases. In phase 3 QUEST (NCT02414854), add-on dupilumab 200/300mg every 2 weeks vs placebo reduced severe exacerbations and improved pre-bronchodilator forced expiratory volume in 1 second (FEV_1_) in patients with uncontrolled, moderate-to-severe asthma. Treatment effects were greater in patients with elevated T2 biomarkers. This post hoc analysis assessed the impact of severe exacerbations on post-bronchodilator FEV_1_ in QUEST patients with T2 inflammatory asthma.

**Methods:** Change from baseline in post-bronchodilator FEV_1_ was assessed in QUEST patients with T2 phenotypes (≥150 eosinophils/µL and/or FeNO ≥ 25ppb or ≥ 300 eosinophils/µL and/or FeNO ≥ 25ppb) who did/did not experience asthma exacerbations during QUEST, and in patients after experiencing a first severe exacerbation.

**Results:** Post-bronchodilator FEV_1_ recovered faster after exacerbation in dupilumab patients. Greater benefits were observed within 6 weeks of exacerbation and were sustained over time. Patients with ≥ 300 eosinophils/µL and/or FeNO ≥ 25ppb at baseline benefited most from dupilumab after first severe exacerbation event (LS mean difference vs placebo [95% CI], ≥ 150 eos/≥ 300 eos: Week3: 0.13L [0.06–0.20]/0.14L [0.06–0.22], Week12: 0.06L [– 0.01 to 0.12]/0.08L [– 0.002 to 0.16], Week24: 0.07L [– 0.02–0.15]/0.09L [– 0.01 to 0.18]). Exacerbator and non-exacerbator post-bronchodilator FEV_1_ was comparable between dupilumab and placebo groups at study baseline. At Week12, improvements in post-bronchodilator FEV_1_ were greater in dupilumab groups, irrespective of exacerbations or baseline eosinophil levels (LS mean difference vs placebo [95% CI]; exacerbators: ≥150 eos/≥300 eos: 0.14L [0.08–0.20], *P* < 0.0001/0.17L [0.10–0.24], *P* < 0.0001; non-exacerbators: ≥ 150 eos/≥300 eos: 0.14L [0.09–0.19], *P* < 0.0001/0.18L [0.13–0.24], *P* < 0.0001). Improvements were sustained over time.

**Conclusions:** Dupilumab reduced the impact of exacerbations and improved post-bronchodilator FEV_1_ in T2 inflammatory asthma patients, regardless of exacerbation status. Lung function improvements after exacerbation were rapid and sustained in dupilumab patients.

### 11 Randomized controlled trial of ragweed sublingual immunotherapy tablet in subpopulation of Canadian children with allergic rhinoconjunctivitis

#### Rémi Gagnon^1^, Anne K. Ellis^2^, David I. Bernstein^3^, Hendrik Nolte^4^

##### ^1^Clinique Spécialisée en Allergie de la Capitale, Québec, QC, Canada, ^2^Department of Medicine, Queen’s University, Kingston, ON, Canada, ^3^Department of Internal Medicine, University of Cincinnati College of Medicine and Bernstein Clinical Research Center, Cincinnati, OH, USA, ^4^ALK, Bedminster, NJ, USA

###### **Correspondance:** Rémi Gagnon

*Allergy, Asthma & Clinical Immunology *2022, **17(Suppl 1)**: 11

**Background:** Post hoc analyses of randomized placebo-controlled trials have demonstrated efficacy and tolerability of the ragweed sublingual immunotherapy (SLIT)-tablet in Canadian adults with ragweed pollen-induced allergic rhinitis/conjunctivitis (AR/C). This analysis evaluated the efficacy and tolerability of the ragweed SLIT-tablet in the subpopulation of Canadian children with AR/C in a previously described randomized, double-blind, placebo-controlled trial.

**Methods:** The trial (NCT02478398) was conducted in North American and European children ages 5–17 years with ragweed pollen-induced AR/C with or without asthma. Participants were randomized to daily ragweed SLIT-tablet (12 Amb a 1-U) or placebo for up to 28 weeks. The primary endpoint was the average total combined score (TCS; sum of rhinoconjunctivitis daily symptom score [DSS] and daily medication score [DMS]) during peak ragweed pollen season (RPS). Post hoc analyses were conducted in the Canadian participants. All statistical analyses were per protocol.

**Results:** Of the 1025 randomized participants, 246 (SLIT-tablet, n = 116; placebo, n = 130) were in the Canadian subpopulation. In the total study population, relative TCS (95% CI) improvement with ragweed SLIT-tablet versus placebo was − 38.3% (− 46.0%, − 29.7%; least square [LS] mean difference, − 2.73; P<0.001) during peak RPS. In the Canadian subpopulation, relative TCS improvements with ragweed SLIT-tablet versus placebo were − 40.8% (− 54.5%, − 20.2%; LS mean difference, − 1.59; P = 0.001) during peak RPS and − 36.6% (− 50.2%, − 16.5%; LS mean difference, − 1.36; P = 0.002) during the entire RPS. DSS and DMS during peak RPS in the Canadian subpopulation improved with SLIT-tablet versus placebo by − 30.6% (− 45.2%, − 7.7%; LS mean difference, − 0.94; P = 0.01) and − 77.2% (− 97.5%, − 44.2%; LS mean difference, − 0.66; P = 0.003), respectively. No events of anaphylaxis, airway compromise, eosinophilic esophagitis, or severe treatment-related systemic allergic reactions were reported in the overall population or Canadian subpopulation.

**Conclusions:** The ragweed SLIT-tablet resulted in clinically meaningful improvement in symptoms, decreased symptom-relieving medication use, and was well tolerated in Canadian children.

## Food Allergy/Anaphylaxis

### 12 Multi-centre real-world experience with epinephrine 0.5 mg dosing for anaphylaxis with allergen immunotherapy

#### Natasha Correa^1^, Ariba Quidwai^1^, Samira Jeimy^1^, Natalie Rondilla^2^, Fred White^1^, William Moote^1^, Mark Kuprowski^1^, Harold Kim^1,3^

##### ^1^Western University, London, ON, Canada, ^2^Grand River Allergy, Kitchener, ON, Canada, ^3^McMaster University, Hamilton, ON, Canada

###### **Correspondance:** Natasha Correa

*Allergy, Asthma & Clinical Immunology* 2022, **17(Suppl 1)**: 12

**Background:** Epinephrine is the first line treatment for anaphylaxis. The optimal dose of epinephrine in anaphylaxis is not well studied. Anaphylaxis occurs in approximately 5% of patients receiving subcutaneous immunotherapy. The recommended dose of epinephrine for adults with anaphylaxis is 0.3 to 0.5 mg. The efficacy and safety of epinephrine 0.5 mg has never been assessed in patients reacting to subcutaneous allergen immunotherapy.

**Methods:** We reviewed the electronic medical records of two outpatient allergy practices for patients who received 0.5 mg intramuscular epinephrine as the initial dose for treatment of anaphylaxis secondary to subcutaneous allergen immunotherapy. Data on patient demographics, vital signs, and patient outcomes were collected. Counts and percentages were computed to summarize the data. Means and 95% confidence intervals (CI) were calculated for vital signs.

**Results:** Thirty-eight patients received an initial dose of 0.5 mg intramuscular epinephrine for allergic reactions to subcutaneous allergen immunotherapy between March 2006 to February 2020. Eleven (30%) and 2 (5%) patients required a second and third dose of epinephrine respectively. Mean systolic blood pressure after administration of epinephrine was 120 mmHg (95% CI: 112–127). Data on heart rate after administration of epinephrine was available for 35 patients. Mean heart rate was 98 beats per minute (95% CI: 88–107). Twenty-two patients (58%) had resolution of symptoms in clinic. Sixteen patients (42%) were transferred to the emergency department with ongoing symptoms. Data on time to symptom resolution was available for 21 patients—the majority (81%) had symptom resolution within 30 minutes. There were no adverse reactions or fatalities.

**Conclusions:** Use of 0.5 mg intramuscular epinephrine to treat anaphylaxis caused by subcutaneous allergen immunotherapy is safe and effective. Further studies on optimal initial dosing of intramuscular epinephrine are needed to reduce the need for repeat doses and emergency department visits due to refractory anaphylaxis

### 13 Prehospital treatment of anaphylaxis from the Cross-Canada Anaphylaxis Registry (C-CARE)

#### Tiffani White^1^, Sofianne Gabrielli^1^, Luca Delli Colli^1^, Christine McCusker^1^, Moshe Ben-Shoshan^1^, Ann E. Clarke^2^, Judy Morris^3^, Jocelyn Gravel^4^, Rodrick Lim^5^, Edmond S. Chan^6^, Ran D. Goldman^7^, Andrew O’Keefe^8^, Jennifer Gerdts^9^, Derek Chu^10^, Julia Upton^11^, Elana Hochstadter^12^, Adam Bretholz^13^, Xun Zhang

##### ^1^Division of Allergy and Clinical Immunology, Department of Pediatrics, Montreal Children’s Hospital, McGill University Health Centre, Montreal, QC, Canada, ^2^Division of Rheumatology, Department of Medicine, Cumming School of Medicine, University of Calgary, Calgary, AB, Canada, ^3^Department of Emergency Medicine, Sacré-Coeur Hôpital, Montreal, QC, Canada, ^4^Department of Pediatric Emergency Medicine, Centre Hospitalier Universitaire Sainte-Justine, Montreal, QC, Canada, ^5^Division of Pediatric Emergency Medicine, Department of Pediatrics, Children’s Hospital at London Health Science Centre,, London, ON, Canada, ^6^Division of Allergy and Immunology, Department of Pediatrics, BC Children’s Hospital, University of British Columbia,, Vancouver, BC, Canada, ^7^Division of Clinical Pharmacology and Emergency Medicine, Department of Pediatrics, BC Children’s Hospital, University of British Columbia, Vancouver, BC, Canada, ^8^Department of Pediatrics, Faculty of Medicine, Memorial University, St. John’s, St. John’s, NL, Canada, ^9^Executive Director, Food Allergy Canada, Toronto, ON, Canada, ^10^Division of Clinical Immunology & Allergy, Department of Medicine, and Department of Health Research Methods, Evidence & Impact, McMaster University, Hamilton, ON, Canada, ^11^Division of Immunology and Allergy, Department of Pediatrics, The Hospital for Sick Children, Department of Paediatrics, University of Toronto,, Toronto, ON, Canada, ^12^Department od Pediatric Emergency Medicine, The Hospital for Sick Children, University of Toronto, Toronto, ON, Canada, ^13^Division of Pediatric Emergency Medicine, Department of Pediatrics, Montreal Children’s Hospital, Montreal, QC, Canada, ^14^Centre for Outcomes Research and Evaluation, Research Institute of McGill University Health Centre, Montreal, QC, Canada

###### **Correspondance:** Tiffani White

*Allergy, Asthma & Clinical Immunology* 2022, **17(Suppl 1)**: 13

**Background:** Data regarding anaphylaxis treatment is often poorly understood. We aimed to assess the effect of prehospital treatment of anaphylaxis on the risk of admission to hospital ward and/or intensive care unit (ICU).

**Methods:** From April 2011 to 2021, 4801 adults (20.5%) and children (79.5%) presenting with anaphylaxis to eleven different emergency departments (ED) across Canada and Israel were recruited as part of the Cross-Canada Anaphylaxis Registry (C-CARE). A standardized form documenting prehospital treatment was obtained and their disposition was documented. Multivariate logistic regression was used to identify associations between prehospital treatment and admission to a hospital ward and/or ICU.

**Results:** Of the recruited patients, the median age was 8.2 years [Interquartile Range (IQR 2.9, 16.5] and 56% were males. The most common prehospital treatments provided were antihistamines (44.9%), epinephrine (35.4%), short acting inhaled beta-agonists (6.3%) and corticosteroids (3.3%). Admission to a hospital ward (4.8%) and/or ICU (0.8%) was infrequent.

Admission to a hospital ward was associated with being an adult and prehospital administration of corticosteroids [adjusted Odds Ratio (aOR) 1.13 (95% CI 1.11, 1.15) and 1.22 (95% CI 1.18, 1.26), respectively], while adjusting for sex, prehospital epinephrine, antihistamines and beta-agonist. Admission to an ICU was associated with prehospital use of corticosteroids [aOR 1.04 (95% CI 1.03, 1.06)], while adjusting for sex, prehospital epinephrine, antihistamines and beta-agonist.

There was a decreased likelihood of being admitted to a hospital ward if the patient was administered prehospital epinephrine or antihistamines [aOR 0.98 (95% CI 0.97–0.99) and 0.97 (95% CI 0.95–0.98), respectively], while adjusting for sex, prehospital epinephrine, antihistamines and beta-agonist

**Conclusions:** This study showed that patients who received corticosteroid for anaphylaxis before ED arrival are at higher risk of hospitalisation while the use of epinephrine and antihistamine decrease the risk of admission.

### 14 The impact of food allergy on health-related quality of life in children and adolescents: a scoping review

#### Katherine Goren^1^, Jennifer L. Protudjer^2,3^, Samira Jeimy^4^

##### ^1^Schulich School of Medicine and Dentistry, Western University, Windsor, ON, Canada, ^2^Department of Pediatrics and Child Health, University of Manitoba, Winnipeg, MB, Canada, ^3^The Children’s Hospital Research Institute of Manitoba, Winnipeg, MB, Canada, ^4^Division of Clinical Immunology and Allergy, Department of Medicine, Western University, London, ON, Canada

###### **Correspondance:** Katherine Goren

*Allergy, Asthma & Clinical Immunology* 2022, **17(Suppl 1)**: 14

**Background:** A diagnosis of food allergy has substantial impacts on the health-related quality of life (HRQOL) and psychosocial well-being of children and adolescents. This scoping review aims to explore the primary literature related to HRQOL and anxiety in children and adolescents diagnosed with food allergy and provide recommendations for improvement.

**Methods:** Searches were conducted in MEDLINE and EMBASE from database inception to March 2021 and reference lists of included articles were screened. Non-intervention studies that reported on quality of life and/or anxiety in food allergic individuals up to age 18 were eligible for inclusion. One reviewer screened studies against predefined inclusion criteria and independently extracted data. Data were analyzed using a qualitative thematic approach.

**Results:** 187 articles were identified, of which 21 (11.2%) articles were included. The Food Allergy Quality of Life Questionnaire (FAQLQ) was the most widely used tool (11/21). Food allergy related anxiety was not widely reported; however, several themes related to HRQOL were identified across studies. Older age (>3 years), female sex, and increased severity of past reactions resulting in epinephrine autoinjector use and/or hospitalization were found to have a negative impact on child and adolescent HRQOL. Increased rates of bullying were not consistently reported across studies; however, bullying through exposure to the allergenic food was reported in one paper. In papers that compared child HRQOL data provided by parents to child HRQOL data provided by children, parents rated their child’s HRQOL better than the children themselves.

**Conclusions:** The recognition of mental health concerns related to decreased HRQOL should be incorporated into food allergy management. Ongoing food allergy mental health education and supports should be provided to families. Collaboration between allergists and mental health professionals is needed to provide effective treatment to children and adolescents with food allergy and develop evidence-based clinical guidelines for effective management of these challenges.

### 15 Oral food challenge outcomes in adults with a history of shrimp allergy

#### Yan J. Liu, Louis Paradis, Normand Dubé, Marie-Soleil Masse, Jean Paradis, Philippe Bégin, François Graham

##### Centre Hospitalier de l’Université de Montréal, Montreal, QC, Canada

###### **Correspondance:** Yan J. Liu

*Allergy, Asthma & Clinical Immunology* 2022, **17(Suppl 1)**: 15

**Background:** Shrimp allergy is one of the most frequently reported food allergies in adults. The objective of this study was to determine shrimp oral food challenge (OFC) outcomes in adults consulting in a tertiary hospital center with a reported history of IgE-mediated reaction to shrimp.

**Methods:** A retrospective chart review was performed for adults who underwent a shrimp OFC at the CHUM allergy clinic between 2017 and 2020. Patients with a convincing history of immediate IgE-mediated reaction to shrimp were included. Patients with shrimp sensitization without a clinical history of reaction to shrimp, with a reaction to seafood other than shrimp, and with reactions non-suggestive of IgE-mediated reactions were excluded.

**Results:** A total of 135 shrimp OFCs were completed, of which 84 had a convincing history of IgE-mediated reaction after shrimp ingestion. Symptoms reported at initial reaction involved cutaneous (62%), gastrointestinal (49%), lower respiratory (35%), cardiovascular (7%) and upper respiratory (1%) systems. Anaphylaxis criteria were present in 35% of patients with a median Brown severity grade^1^of 2. The median age (range) at initial reaction and time of OFC were 28 (3–70) and 36 (14–76) years, respectively. Seventy-seven percent (36/47) were sensitized to dust mites. Six patients (7%) had positive OFCs with a median severity grade of 2. Two patients with negative low dose OFCs underwent oral immunotherapy and subsequently reacted to shrimp at home. Eleven patients (13%) had transient isolated oral symptoms but were able to complete OFC. Median skin test size was significantly higher in the shrimp-allergic group (4.5 mm; 0–10) than in the shrimp tolerant group (0 mm; 0–11, p = 0.0444).

**Conclusions:** The rate of positive OFCs to shrimp in adults with a reported history of immediate reaction to shrimp was low. These findings may suggest a favorable natural history for adult-onset shrimp allergy, which warrants further prospective studies.

**References:**

1. Brown SG. Clinical features and severity grading of anaphylaxis. J Allergy Clin Immunol. 2004 Aug;114(2):371–6.

### 16 Temporal trends of skin prick tests for food induced allergic reactions/anaphylaxis

#### Pasquale Mulé^1^, Bruce Mazer^1^, Danbing Ke^1^, Duncan Lejtenyi^1^, Liane Beaudette^1^, Julia Upton^2^, Edmond S. Chan^3^, Ann Clarke^4^, Xun Zhang^1^, Sofianne Gabrielli^1^, Moshe Ben-Shoshan^1^

##### ^1^Division of Allergy and Clinical Immunology, Department of Pediatrics, Montreal Children’s Hospital, McGill University Health Centre, Montreal, QC, Canada, ^2^Division of Immunology and Allergy, Department of Pediatrics, The Hospital for Sick Children, Department of Pediatrics, University of Toronto, Toronto, ON, Canada, ^3^Division of Allergy and Immunology, Department of Pediatrics, BC Children’s Hospital, University of British Columbia, Vancouver, BC, Canada, ^4^Division of Rheumatology, Department of Medicine, Cumming School of Medicine, University of Calgary, Calgary, AB, Canada

###### **Correspondance:** Pasquale Mulé

*Allergy, Asthma & Clinical Immunology* 2022, **17(Suppl 1)**: 16

**Background:** Although clinical practice recommends performing skin prick tests (SPTs) at least four weeks following an allergic reaction, it is not established if SPTs done earlier are useful in detecting the culprit allergen. We aimed to assess SPTs for the culprit allergen as a function of time after food induced allergic reaction/ anaphylaxis.

**Methods:** 35 patients with a history of food allergies, and who underwent a food challenge at the Montreal Children’s Hospital were recruited. SPTs were assessed at baseline, 1–2 hours, 1–2 weeks, and 30–40 days after the onset of allergic reaction/anaphylaxis which occurred during the food challenge. All SPTs performed 1–2 hours post-reaction were conducted within one hour of antihistamine administration. A non-parametric test (Wilcoxon signed-rank test) was used to assess changes in wheal diameter between the time intervals at a significance level of 0.05.

**Results:** Patient ages ranged from 5 to 19 years old (mean 12.3, 54% male). Allergens used included eggs (31%), milk (17%), hazelnuts (6%), and peanuts (46%). All patients developed objective allergic symptoms during the food challenge, and 27 cases of anaphylaxis were observed.

The baseline mean wheal diameter was 5.9 mm (SD 3.5). Mean wheal diameter significantly decreased to 3.3 mm (SD 2.7, p = 0.040) 1–2 hours post-reaction; creating a false negative result. However, mean wheal diameter significantly increased to 4.9 mm (SD 2.3, p = 0.013) 1–2 weeks post-reaction, when compared to 1–2 hours post-reaction. No significant difference in SPT results were measured between 1–2 weeks and 30–40 days post-reaction (p = 0.673).

**Conclusions:** SPT sensitivity may decrease 1–2 hours post-reaction. However, this hyposensitivity is transient, and SPTs become an accurate diagnosis of food allergy within 1–2 weeks. This challenges the traditionally held viewpoint regarding the timing of SPTs.

### 17 Sesame desensitization in children in real-world clinical practice

#### Dima Elgendy^1^, Karen Sigman^1^, Christine McCusker^1^, Sofianne Gabrielli^1^, Sarife Saker^1^, Xun Zhang^2^, Moshe Ben-Shoshan^1^

##### ^1^Division of Allergy and Clinical Immunology, Department of Pediatrics, Montreal Children’s Hospital, McGill University Health Centre, Montreal, QC, Canada, ^2^Centre for Outcomes Research and Evaluation, Research Institute of McGill University Health Centre, Montreal, QC, Canada

###### **Correspondance:** Dima Elgendy

*Allergy, Asthma & Clinical Immunology* 2022, **17(Suppl 1)**: 17

**Background:** As of 2020, 0.3% of Canadian children are reported to have sesame allergy. Although studies suggest that oral immunotherapy (OIT) should be incorporated into clinical practice, a major limitation of all current OIT protocols is the risk of anaphylaxis. We aim to explore modified OIT protocols that will promote a safer approach to sesame desensitization, without compromising effectiveness.

**Methods:** Children less than 12 years old with a history-based sesame allergy diagnosis and a positive sesame skin prick test were recruited at hospital and community-based allergy clinics. Upon initial visit, a dose of 5–12mg of sesame protein was introduced and guardians filled out a quality-of-life questionnaire. Patients would then continue the dose for 2–5 weeks at home, fill out a symptom diary, and return to the clinic to receive a new dose. R (v4.0.2) was used to analyze the demographics of the study population in addition to the safety of the modified OIT protocols used.

**Results:** From January 29 to June 17, 2021, 11 children (45.5% male; median age: 1 year, 10 months) were recruited. All 11 patients (100%) had eczema, two (18.2%) had asthma, and eight (72.7%) had other food allergies (mainly peanut (n = 5; 62.5%), cashew (n = 3; 37.5%), and pistachio (n = 3; 37.5%)). OIT was performed using 3 strategies: initial doses were either sesame seeds, tahini muffin, or hummus. Children were desensitized aiming to reach 2 teaspoons of hummus or equivalent of sesame protein (50mg). To date, five patients (45.5%), after on average 3.2 visits, have reached this point. Among the 964 total intake doses of sesame, two cases of mild non-anaphylactic allergic reaction and one case of moderate anaphylaxis occurred.

**Conclusions:** OIT is safe as a method of sesame allergy desensitization. Further studies are needed to determine which of the strategies used are optimal for children.

### 18 Milk-induced anaphylaxis among children from the Cross-Canada Anaphylaxis Registry (C-CARE)

#### Rose Di Ioia^1^, Sofianne Gabrielli^1^, Anne E. Clarke^2^, Judy Morris^3^, Jocelyn Gravel^4^, Rodrick Lim^5^, Edmond S. Chan^6^, Ran D. Goldman^7^, Andrew O’Keefe^8^, Jennifer Gerdts^9^, Derek Chu^10^, Julia Upton^11^, Elena Hochstadter^12^, Adam Bretholz^13^, Christine McCusker^1^, Xun Zhang^14^, Moshe Ben-Shoshan^1^

##### ^1^Division of Allergy and Clinical Immunology, Department of Pediatrics, Montreal Children’s Hospital, McGill University Health Centre, Montreal, QC, Canada, ^2^Division of Rheumatology, Department of Medicine, Cumming School of Medicine, University of Calgary, Calgary, AB, Canada, ^3^Department of Emergency Medicine, Sacré-Coeur Hospital, Montreal, QC, Canada, ^4^Division of Pediatric Emergency Medicine, Department of Pediatrics, Centre Hospitalier Universitaire Sainte-Justine, Montreal, QC, Canada, ^5^Division of Pediatric Emergency Medicine, Department of Pediatrics, Children’s Hospital at London Health Science Centre, London, ON, Canada, ^6^Division of Allergy and Immunology, Department of Pediatrics, BC Children’s Hospital, University of British Columbia, Vancouver, Vancouver, BC, Canada, ^7^Division of Clinical Pharmacology and Emergency Medicine, Department of Pediatrics, BC Children’s Hospital, University of British Columbia, Vancouver, BC, Canada, ^8^Department of Pediatrics, Faculty of Medicine, Memorial University, Saint-John’s, NL, Canada, ^9^Executive Director, Food Allergy Canada, Toronto, ON, Canada, ^10^Division of Clinical Immunology & Allergy, Department of Medicine, and Department of Health Research Methods, Evidence & Impact, McMaster University, Hamilton, ON, Canada, ^11^Division of Immunology and Allergy, Department of Pediatrics, The Hospital for Sick Children, Department of Paediatrics, University of Toronto, Toronto, ON, Canada, ^12^Department of Pediatric Emergency Medicine, The Hospital for Sick Children, University of Toronto, Toronto, ON, Canada, ^13^Division of Pediatric Emergency Medicine, Department of Pediatrics, Montreal Children’s Hospital, Montreal, QC, Canada, ^14^Centre for Outcomes Research and Evaluation, Research Institute of McGill University Health Centre, Montreal, QC, Canada

###### **Correspondance:** Rose Di Ioia

*Allergy, Asthma & Clinical Immunology* 2022, **17(Suppl 1)**: 18

**Background:** Milk is a major cause of anaphylaxis and anaphylaxis-related fatalities in children. However, data on milk-induced anaphylaxis in Canada are sparse. We aimed to assess clinical and demographic characteristics of children (0–17 years old) presenting to emergency departments (EDs) with milk-induced anaphylaxis and determine risk factors associated with multiple doses of epinephrine in the hospital setting.

**Methods:** Between 2011 and 2020, children presenting to seven EDs in five provinces were recruited as part of the Cross-Canada Anaphylaxis Registry (C-CARE). A standardized form documenting triggers, symptoms and management was collected. A logistic regression model was used to determine factors associated with requiring more than one epinephrine dose in the ED following a milk reaction.

**Results:** Data were collected from 247 pediatric patients (median age 3.60, interquartile range (IQR) [1.10, 8.15]) presenting with milk-induced anaphylaxis. Among all, 93.9% of patients presented following oral ingestion of milk, 3.2% after cutaneous exposure. The symptoms most frequently experienced were urticaria (66.0%), angioedema (48.2%), pruritus (41.3%), gastrointestinal symptoms (38.1%) and breathing difficulty (36.8%). Epinephrine was used in 51.0% of patients outside the ED and used in 44.5% in the ED. Among 110 patients requiring epinephrine in the ED, 14 required multiple doses: 11 patients were given 2 doses, 3 were given 3 doses.

Risk factors for multiple epinephrine doses were known allergy to eggs (adjusted odds ratio (aOR) 1.86 (1.53; 2.26)), to wheat (aOR 1.86 (1.53; 2.26)) as well as ingestion of milk in the form of baked goods (aOR 1.18 (1.01; 1.39)) when adjusting for age, sex and asthma.

**Conclusions:** Consumption of milk from baked goods, known allergy to eggs and to wheat are associated with the need to use more than one dose of epinephrine for anaphylaxis in EDs. Prompt epinephrine use by caregivers when children are exposed to milk from baked goods is essential.

### 19 Rates, triggers and clinical characteristics of anaphylaxis over time at Montreal Children’s Hospital

#### Adnan Al Ali^1^, Moshe Ben-Shoshan^1^, Christine McCusker^1^, Sofianne Gabrielli^1^, Luca Delli Colli^1^, Ann E. Clarke^2^, Judy Morris^3^, Jocelyn Gravel^4^, Rodrick Lim^5^, Edmond S. Chan^6^, Ran D. Goldman^7^, Andrew O’Keefe^8^, Jennifer Gerdts^9^, Derek K. Chu^10^, Julia Upton^11^, Elana Hochstadter^12^, Jocelyn Moisan^13^, Adam Bretholz^14^, Xun Zhang^15^, Jennifer L. Protudjer^16^, Elissa M. Abrams^17^, Elinor Simons^17^

##### ^1^Division of Allergy and Clinical Immunology, Department of Pediatrics, Montreal Children’s Hospital, McGill University Health Centre, Montreal, QC, Canada, ^2^Division of Rheumatology, Department of Medicine, Cumming School of Medicine, University of Calgary, Calgary, AB, Canada, ^3^Department of Emergency Medicine, Sacré-Coeur Hôpital,, Montreal, QC, Canada, ^4^Department of Pediatric Emergency Medicine, Centre Hospitalier Universitaire Sainte-Justine, Université de Montréal, Montreal, QC, Canada, ^5^Division of Pediatric Emergency Medicine, Department of Pediatrics, Children’s Hospital at London Health Science Centre, London, Ontario, ON, Canada, 6. Division of Allergy and Immunology, Department of Pediatrics, BC Children’s Hospital, University of British Columbia, Vancouver, British Columbia, BC, Canada, ^7^Division of Clinical Pharmacology and Emergency Medicine, Department of Pediatrics, BC Children’s Hospital, University of British Columbia, Vancouver, British Columbia, BC, Canada, ^8^Department of Pediatrics, Faculty of Medicine, Memorial University, St. John’s,, Newfoundland & Labrador, NL, Canada, ^9^Executive Director, Food Allergy Canada,, Toronto, Ontario, ON, Canada, ^10^Division of Clinical Immunology & Allergy, Department of Medicine, and Department of Health Research Methods, Evidence & Impact, McMaster University,, Hamilton, ON, ON, Canada, ^11^Division of Immunology and Allergy, Department of Pediatrics, The Hospital for Sick Children, Department of Paediatrics, University of Toronto, Toronto, ON, Canada, ^12^Department of Pediatric Emergency Medicine, The Hospital for Sick Children, University of Toronto, Toronto, ON, Canada, ^13^Regional Medical Director of Emergency Medical Services of Outaouais, Outaouais, QC, Canada, ^14^Division of Pediatric Emergency Medicine, Department of Pediatrics, Montreal Children’s Hospital, Montreal, QC, Canada, ^15^Centre for Outcomes Research and Evaluation, Research Institute of McGill University Health Centre, Montreal, QC, Canada, ^16^Department of Pediatrics and Child Health, Children’s Hospital Research Institute of Manitoba, Winnipeg, MB, Canada, ^17^Department of Pediatrics and Child Health, Section of Allergy and Clinical Immunology, Children’s Hospital Research Institute of Manitoba, Winnipeg, MB, Canada

###### **Correspondance:** Adnan Al Ali

*Allergy, Asthma & Clinical Immunology* 2022, **17(Suppl 1)**: 19

**Background:** Anaphylaxis is a systemic and life-threatening allergic reaction. Data are sparse regarding variations in anaphylaxis rates on a year-by-year basis. We aimed to assess changes in yearly rates of anaphylaxis in a pediatric Emergency Department (ED) in Montreal, Canada.

**Methods:** Cases of anaphylaxis presenting to the Montreal Children’s Hospital between April 2011 and May 2021 were recruited prospectively and retrospectively. Data were obtained via a standardized recruitment form. Descriptive analysis was used to assess the trend of anaphylaxis in relation to clinical triggers. Statistical significance was calculated using Pearson’s chi-squared test.

**Results:** Among 760,079 ED visits between April 2011 and May 2021, 2573 (34.95% of whom recruited prospectively) presented with anaphylaxis, The median age was 5.70 years (IQR: 2.20, 11.70), and 58.66% were males. The relative frequency of anaphylaxis cases with respect to ED visits doubled between 2011–2015, from 0.22 (95% CI, 0.19, 0.26) to 0.42% (95% CI, 0.38, 0.46). From 2015 to 2020 the rate was stable. Importantly, during the COVID-19 pandemic, beginning March 2020, the total absolute number of anaphylaxis and emergency cases declined, leading to a significant decrease in anaphylaxis cases by 24 cases per month(p < 0.05) and by 0.5% among ED visits (p < 0.05). Foods, (85.75%),drugs (2.81%) and venom (1.60%). Peanut (19.08%) and tree nuts (14.36%) were the major triggers of food-induced anaphylaxis. Most anaphylactic reactions were moderate (71.81%), defined as crampy abdominal pain, diarrhea, recurrent vomiting, hoarseness, “barky” cough, difficulty swallowing, dyspnea, moderate wheezing, and lightheadedness.

**Conclusions:** The rate of anaphylaxis has plateaued over the last six years, representing increased awareness, modifications in food introduction strategies or lifestyle changes. The observed decrease in anaphylaxis during COVID-19 may reflect hesitancy in arrival for management in a hospital setting, given the similar decrease in ER visits.

### 20 Sesame-induced anaphylaxis in pediatric patients from Cross-Canada Anaphylaxis Registry (C-CARE)

#### Carly Sillcox^1^, Sofianne Gabrielli^1^, Ann E. Clarke^2^, Judy Morris^3^, Jocelyn Gravel^17^, Rodrick Lim^4^, Edmond S. Chan^5^, Ran D. Goldman^6^, Andrew O’Keefe^7^, Jennifer Gerdts^8^, Derek K. Chu^9^, Julia Upton^10^, Elana Hochstadter^11^, Jocelyn Moisan^12^, Adam Bretholz^13^, Christine McCusker^1^, Xun Zhang^14^, Jennifer L. Protudjer^15^, Elissa M. Abrams^16^, Elinor Simons^16^, Moshe Ben-Shoshan^1^

##### ^1^Division of Allergy and Clinical Immunology, Department of Pediatrics, Montreal Children’s Hospital, McGill University Health Centre, Montreal, QC, Canada, ^2^Division of Rheumatology, Department of Medicine, Cumming School of Medicine, University of Calgary, Calgary, AB, Canada, ^3^Department of Emergency Medicine, Sacré-Coeur Hôpital, Montreal, QC, Canada, ^4^Division of Pediatric Emergency Medicine, Department of Pediatrics, Children’s Hospital at London Health Science Centre, London, ON, Canada, ^5^Division of Allergy and Immunology, Department of Pediatrics, BC Children’s Hospital, University of British Columbia, Vancouver, BC, Canada, ^6^Division of Clinical Pharmacology and Emergency Medicine, Department of Pediatrics, BC Children’s Hospital, University of British Columbia, Vancouver, BC, Canada, ^7^Department of Pediatrics, Faculty of Medicine, Memorial University, St. John’s, NL, Canada, ^8^Executive Director, Food Allergy Canada, Toronto, ON, Canada, ^9^Division of Clinical Immunology & Allergy, Department of Medicine, and Department of Health Research Methods, Evidence & Impact, McMaster University, Hamilton, ON, Canada, ^10^Division of Immunology and Allergy, Department of Pediatrics, The Hospital for Sick Children, Department of Paediatrics, University of Toronto, Toronto, ON, Canada, ^11^Department of Pediatric Emergency Medicine, The Hospital for Sick Children, University of Toronto, Toronto, ON, Canada, ^12^Regional Medical Director of Emergency Medical Services of Outaouais, Outaouais, QC, Canada, ^13^Division of Pediatric Emergency Medicine, Department of Pediatrics, Montreal Children’s Hospital, Montreal, QC, Canada, ^14^Centre for Outcomes Research and Evaluation, Research Institute of McGill University Health Centre, Montreal, QC, Canada, ^15^Department of Pediatrics and Child Health, Children’s Hospital Research Institute of Manitoba, Winnipeg, MB, Canada, ^16^Department of Pediatrics and Child Health, Section of Allergy and Clinical Immunology, Children’s Hospital Research Institute of Manitoba, Winnipeg, MB, Canada, ^17^Department of Pediatric Emergency Medicine, Centre Hospitalier Universitaire Sainte-Justine, Université de Montréal, Montréal, QC, Canada

###### **Correspondance:** Carly Sillcox

*Allergy, Asthma & Clinical Immunology* 2022, **17(Suppl 1)**: 20

**Background:** Sesame is a major anaphylaxis food trigger and a priority allergen. We assessed the clinical characteristics and management of pediatric patients with sesame-induced anaphylaxis and identified factors associated with epinephrine treatment.

**Methods:** Children with sesame-induced anaphylaxis to seven Emergency Departments (ED) in four Canadian provinces and one Emergency Medical Services (EMS) in Quebec’s Outaouais region were enrolled in the Cross-Canada Anaphylaxis Registry (C-CARE). C-CARE includes nationwide anaphylaxis data from April 27, 2011-January 31, 2021. Data was obtained with standardized recruitment forms and included symptoms, severity, triggers, and management plan. Multivariate logistic regression was used to assess associations with epinephrine treatment before and in the ED.

**Results:** Among 130 children presenting to Canadian EDs between 2011 and 2021 with sesame-induced anaphylaxis, 61.5% were males with mean age of 5.0 years (SD 4.9). Sesame-induced reactions accounted for 4.0% of all food-induced reactions. The most common sesame triggers were hummus/tahini (53.5%), bagel sesame grains (4.4%), bread (3.5%), and cookies (3.5%). Patients with previous knowledge of their allergy accounted for 37.7%. Epinephrine was used in 32.3% of cases prior to ED and 47.7% in the ED. Among all cases, 20.0% did not receive epinephrine. Almost half (50.8%) were referred to an allergist for evaluation. Patients most likely to be treated with epinephrine pre-hospital included those with a known allergy and males [adjusted Odds Ratio (aOR) 1.36 (95% CI 1.11, 1.68) and aOR 1.27 (95% CI 1.08, 1.50), respectively] with adjustment for age, severity, reaction location, and epinephrine prescription. Older patients were more likely to receive epinephrine treatment in the ED [aOR 1.02 (95% CI 1.00, 1.04)] with adjustment for sex, severity, and known allergy.

**Conclusions:** In Canada, the major trigger of sesame-induced anaphylaxis is tahini, often found in hummus. Educational programs on prompt epinephrine use and increased product labelling policies are required to limit sesame reactions in the community.

### 21 Considerations and recommendations for healthcare professionals caring for patients with food allergy

#### Tessa Memauri^1^, Michael Golding^1^, Jennifer D. Gerdts^2^, Elinor Simons^1^, Elissa Abrams^1^, Susan J. Elliot^3^, Leslie E. Roos^4^, Harold Kim^5^, Jennifer L. Protudjer^1^

##### ^1^Children’s Hospital Research Institute of Manitoba, Winnipeg, MB, Canada, ^2^Food Allergy Canada, Toronto, ON, Canada, ^3^University of Waterloo, Waterloo, ON, Canada, ^4^University of Manitoba, Winnipeg, MB, Canada, ^5^University of Western Ontario, London, ON, Canada

###### **Correspondance:** Tessa Memauri

*Allergy, Asthma & Clinical Immunology* 2022, **17(Suppl 1)**: 21

**Background:** Food allergy imparts a significant burden worldwide, albeit disproportionately affecting children and their families. Continued research supports the association between poor quality-of-life and food allergy, despite advances in related healthcare. Currently, little is understood about how healthcare providers describe standard practices for pediatric food allergy, and how limitations in healthcare structures may compromise quality of care. To better understand how pediatric food allergy care can be improved, we considered healthcare providers’ perceptions of the current standard of care in order to identify gaps in professional knowledge and public resources specific to food allergy.

**Methods:** We performed individual interviews (n = 6) and profession-specific focus groups (n = 2, involving 7 participants) of healthcare providers, based in Manitoba, Canada, who care for children with food allergies. Healthcare providers included pediatric allergists (n = 4), allergy nurse educators (n = 5), and clinical dieticians (n = 4). Interviews/focus groups were audio-recorded and transcribed verbatim. Data were analysed using thematic analysis via an inductive approach, from which we identified main themes.

**Results:** We identified 3 main themes. The first theme, *Bridging professional knowledge gaps,* highlights areas where interviewees felt that additional support or training was needed. The second theme, *Limitations within the current healthcare system, *identifies barriers within the aforementioned professions that create obstacles in delivering superior patient care. Examples ranged from “enormous” waitlists to time constraints making psychological care “not feasible in [the] current scope of my practice”*. *Lastly*, Incorporating a multidisciplinary, patient-centered approach to pediatric food allergy care*, lists the recommendations provided by our study participants on how they perceived professional practice shortcomings could be improved.

**Conclusions:** Herein, healthcare providers collectively described standard practice limitations and systemic flaws within the field of pediatric food allergy care. Moreover, they advocated for improved public and professional food allergy education and a multidisciplinary approach to patient care.

### 22 Peanut desensitization in preschool and older children in real-world clinical practice

#### Sarife Saker^1^, Christine McCusker^1^, Moshe Ben-Shoshan^1^, Sofianne Gabrielli^1^, Dima Elgendy^1^, Xun Zhang^2^, Karen Sigman^1^

##### ^1^Division of Allergy and Clinical Immunology, Department of Pediatrics, Montreal Children’s Hospital, McGill University Health Centre, Montreal, QC, Canada, ^2^Centre for Outcomes Research and Evaluation, Research Institute of McGill University Health Centre, Montreal, QC, Canada

###### **Correspondance:** Sarife Saker

*Allergy, Asthma & Clinical Immunology* 2022, **17(Suppl 1)**: 22

**Background:** Peanut allergy is the most common food allergy in Canadian children and accounts for a significant number of fatalities related to food allergy. Peanut desensitization in young children has been shown to be effective and safe. However, as children grow into adolescents, they encounter more difficulties and have greater risks for severe reactions. The primary aim of this study was to evaluate the safety of peanut desensitization in preschool and school-aged children.

**Methods:** Children, up to 15 years of age, diagnosed with peanut allergy based on history and a positive peanut skin prick test were enrolled from academic and community allergic clinics. Patients underwent peanut desensitization on a four-week interval basis. Upon initial visit, children were introduced to a dose of 5–10mg of peanut flour or peanut puff and they gradually progressed to more concentrated forms of peanut protein (peanut butter). An endpoint of 2 teaspoons of peanut butter (4000 mg of peanut protein) was defined. An initial quality-of-life questionnaire and a daily symptom diary were distributed among parents. Demographics of the study population and adverse reactions were evaluated using R (v4.0.2).

**Results:** From September 25, 2020 to June 10, 2021, 80 children were recruited for peanut desensitization. The mean age was 18 months and 55% were male. Atopic dermatitis (85.3%) and asthma (14.2%) were the main associated comorbidities. The most common co-allergies were egg (57.8%), milk (26.3%), and cashew (21%). At this point, one patient has reached 1 teaspoon of peanut butter. To date, among the 11105 total intake doses of peanut given, 150 non-anaphylactic reactions (4%) and 12 anaphylactic reactions (0.01%) were reported. No severe anaphylaxis occurred (defined as hypotension, cyanosis or loss of consciousness).

**Conclusions:** Our study supports the safety of peanut desensitization using concentrated forms of peanut protein in preschool and older children, in real-world clinical practice.

### 23 Food allergy education and management in schools: a scoping review on current practices and gaps

#### Mae Jhelene Santos^1^, Kaitlyn A. Merrill^2^, Jennifer D. Gerdts^3^, Moshe Ben-Shoshan^4^, Jennifer L. Protudjer^5,6,7^

##### ^1^Department of Food and Human Nutritional Sciences,University of Manitoba, Winnipeg, MB, Canada, ^2^Department of Biochemistry, University of Winnipeg, Winnipeg, MB, Canada, ^3^Executive Director, Food Allergy Canada, Toronto, ON, Canada, ^4^Division of Pediatric Allergy and Clinical Immunology, Department of Pediatrics, McGill University Health Center, Montreal, QC, Canada, ^5^Department of Pediatrics and Child Health, Rady Faculty of Health Sciences, Winnipeg, MB, Canada, ^6^The Children’s Hospital Research Institute of Manitoba, Winnipeg, MB, Canada, ^7^The Centre for Allergy Research, Stockholm, Sweden

###### Correpondance: Mae Jhelene Santos

*Allergy, Asthma & Clinical Immunology* 2022, **17(Suppl 1)**: 23

**Background:** Approximately 20% of anaphylactic reactions occur in school settings. This observation is concerning because food allergy (FA) and anaphylaxis management vary by jurisdiction, and consequently, amongst teachers and school staff. Currently, there are no formal synthesis of published studies in this area. To address this gap, we aimed to report on the baseline FA and anaphylaxis management knowledge, and differences post-educational intervention amongst teachers and school staff.

**Methods:** Guided by the PRISMA-Scoping Review statement, we conducted a search on February 19, 2021 using the OVID-MedLine, Scopus, PsycInfo databases for articles published in 2006 and beyond. Eligible English and French articles from North America, Europe and Australia were included. Title/ abstract screening were done for 2,010 articles, of which 77 were moved to full-texting by two independent reviewers (MS/KM). No third reviewer was needed to resolve conflicts. Reviewers for French articles (JP) were inconsistent with English ones. Results were reported descriptively and thematically.

**Results:** Eight studies conducted pre-post educational intervention surveys. Four studies provided epinephrine autoinjector (EAI) training. Session topics included: FA definition, diagnosis, allergenic foods, symptom recognition, medications and EAI administration. Five (62.5%) studies reported participants previously had students with FA, while related training was variable (37–64%) among teachers and school staff. One study reported associated emotions with FA were “concern” and “anxiety”. All studies reported better knowledge in teachers and school staff who received intervention, better FA-related attitudes and beliefs (12.5%), better self-efficacy and confidence levels (37.5%). Notably, 25% of studies demonstrated higher pre-post scores within teachers from economically-disadvantaged school areas.

**Conclusions:** Teachers and school staff have more FA-related experiences than training. Standardized education sessions and EAI training may promote safe schools for individuals with food allergy. Moreover, training may provide teachers and school staff with confidence and self-efficacy to effectively prevent and manage FA emergencies in schools.

### 24 Rate of dose increment is associated with risk of severe adverse reactions during oral food challenge

#### Danbing Ke^1^, Pasquale Mule^2^, Duncan Lejtenyi^1^, Liane Beaudette^1^, Christine McCusker^3,1^, Julia E. Upton^4,9^, Edmond S. Chan^5,8^, Ann Clarke^6^, Phillippe Begin^7,10^, Eyal Grunebaum^4,9^, Bruce Mazer^1,2,3^, Moshe Ben-Shoshan^3,1^

##### ^1^MUHC-RI, Montreal, QC, Canada, ^2^McGill University, Montreal, QC, Canada, ^3^Montreal Children’s Hospital, Montreal, QC, Canada, ^4^Hospital for Sick Children, Toronto, ON, Canada, ^5^British Columbia Children’s Hospital, Vancoveur, BC, Canada, ^6^University of Calgary, Calgary, AB, Canada, ^7^Universite de Montreal, Montreal, QC, Canada, ^8^University of British Columbia, Vancoveur, BC, Canada, ^9^Univerity of Toronto, Toronto, ON, Canada, ^10^CHU Sainte-Justine, Montreal, QC, Canada

###### **Correspondance:** Danbing Ke

*Allergy, Asthma & Clinical Immunology* 2022, **17(Suppl 1)**: 24

**Background:** Oral food challenge (OFC) is the gold standard for diagnosis of food allergy. Its process is largely defined empirically and severe adverse reactions during OFC remain unpredictable.

**Methods:** Placebo-controlled OFC was conducted as part of a series of Canadian multi-center oral immunotherapy trials to confirm allergy to four common food allergens, namely, hen’s egg, cow’s milk, peanut, and hazelnut. All food doses and corresponding times of dosing were documented as well as adverse reactions during OFC. Incremental doses over time were plotted to calculate the relative growth rate (RGR), a mathematical constant measuring the exponential dose increase for each OFC. The association between adverse reaction severity, defined by the number of epinephrine dose(s), and OFC process parameters including RGR was investigated using logistic regression.

**Results:** A total of 142 positive challenges (18, 6, 91, and 27 for egg, hazelnut, milk, and peanut respectively) were recorded. In 19, OFC was stopped before the third challenge dose due to adverse reactions and none required multiple doses of epinephrine (p = 0.025, Fisher’s exact test). In those involving at least three challenge doses (n = 123), mean RGRs for egg (n = 17), hazelnut (n = 6), milk (n = 76), and peanut (n = 24) OFCs were 2.1, 2.4, 3.7, and 2.4 respectively. In total, 28 patients received multiple doses of epinephrine, accounting for 5.6% (n = 1), 22.0% (n = 20), and 25.9% (n = 7) of OFCs for egg, milk, and peanut respectively. The RGR was associated with these severe adverse reactions (p = 0.004, adjusted odds ratio = 2.34, 95% CI 1.38–4.42).

**Conclusions:** The RGR measures the intensity of dose increment for each OFC and can be used to better standardize OFC or to guide modification of OFC process parameters by administering smaller food doses at each step and/or by increasing interval time between doses, to make an OFC safer.

### 25 Higher and lower income families with food allergy report different food-related cost changes following COVID-19

#### Michael A. Golding^1,2^, Cathérine Lemoine-Courcelles^1,2^, Elissa M. Abrams^1,2,3^, Moshe Ben-Shoshan^4^, Philippe Bégin^5^, Edmond S. Chan^3,6^, Derek K. Chu^7^, Jennifer Gerdts^9^, Beatrice Povolo^9^, Harold L. Kim^7,10^, Elinor Simons^1,2^, Julia Upton^11^, Jennifer Protudjer^1,2,12^

##### ^1^University of Manitoba, Winnipeg, MB, Canada, ^2^Children’s Hospital Research Institute of Manitoba, Wininpeg, MB, Canada, ^3^University of British Columbia, Vancouver, BC, Canada, ^4^McGill University, Montréal, QC, Canada, ^5^Université de Montréal, Montréal, QC, Canada, ^6^BC Children’s Hospital Research Institute, Vancouver, BC, Canada, ^7^McMaster University, Hamilton, ON, Canada, ^8^The Research Institute of St. Joe’s Hamilton, Hamilton, ON, Canada, ^9^Food Allergy Canada, Toronto, ON, Canada, ^10^Western University, London, ON, Canada, ^11^University of Toronto, Toronto, ON, Canada, ^12^Karolinska Institutet, Stockholm, Sweden

###### **Correspondance:** Michael A. Golding

*Allergy, Asthma & Clinical Immunology* 2022, **17(Suppl 1)**: 25

**Background:** It is not clear how the COVID-19-related changes in the price and availability of food affect families with food allergy. Therefore, the current study investigated how COVID-19 has impacted the food-related costs of higher and lower income households with food allergy.

**Methods:** We recruited 102 households with at least one food-allergic member to complete an online survey that assessed their food shopping and preparation habits prior to and during the COVID-19 pandemic. Changes in direct (i.e., out-of-pocket) and indirect (i.e., loss of time) food costs were described using means +/− SDs. We also used multiple regression analyses to identify patient factors associated with cost changes. All analyses were stratified based on whether the respondent’s monthly household income fell above or below the median value of $5830 (CAD).

**Results:** Households in both income strata reported lower indirect shopping costs, but higher indirect food prepa-ration costs and direct grocery costs following the outbreak of COVID-19. Regression analyses indicated that lower income food-allergic families with difficulties finding their typical grocery products reported a greater post-COVID-19 increase in direct grocery costs in comparison to those without such difficulties (+$164.31 vs. − $31.94, p = 0.03). Results also revealed that lower income households with an allergy to milk, wheat, or eggs experienced a larger increase in indirect food preparation costs following COVID-19 relative to those with other food allergies (+$244.58 vs. +$20.28, p = 0.03). Lastly, higher income households who had difficulties finding their typical grocery items reported greater indirect shopping costs after the emergence of COVID-19 contrary to those without such difficulties (+$34.09 vs. − $69.80, p = 0.04).

**Conclusions:** Findings suggest that food-related disruptions linked to COVID-19 affect higher and lower income households with food allergy differently. While more research is needed, the current findings may help inform programs aimed at promoting the well-being of households with food allergy.

### 26 A comparison of the burden of allergies to priority versus both priority and non-priority allergens in children with multiple food allergies

#### Hailey V. Hildebrand^1,2^, Elissa M. Abrams^1,2^, Jennifer Gerdts^3^, Beatrice Povolo^3^, Harold Kim^4,5^, Jennifer L. Protudjer^1,2,6^

##### ^1^University of Manitoba, Winnipeg, MB, Canada, ^2^The Children’s Hospital Research Institute of Manitoba, Winnipeg, MB, Canada, ^3^Food Allergy Canada, Toronto, ON, Canada, ^4^Western University, London, ON, Canada, ^5^McMaster University, Hamilton, ON, Canada, ^6^George and Fay Yee Centre for Healthcare Innovation, Winnipeg, MB, Canada

###### **Correspondance:** Hailey V. Hildebrand

*Allergy, Asthma & Clinical Immunology* 2022, **17(Suppl 1)**: 26

**Background:** Canadian labelling regulations require priority allergens to be listed if used as a component of an ingredient; however, there are no such requirements for non-priority allergens. This difference suggests a gap in non-priority allergen labelling. We aimed to compare the burden of priority allergens only versus those with both priority and non-priority allergies in Canadian children with multiple food allergies.

**Methods:** This study uses data from NUANCES: multidimeNsional bUrden of Allergies in CanadiaN ChildrEn and adultS, an online survey of adults/children with multiple food allergies. Our study population was restricted to children only. Families reporting a single food allergy were excluded. Quantitative data were described using n/N and %. This study was approved by The University of Manitoba Health Research Ethics Board (HS23109 (H2019:317)).

**Results:** In our sample of 123, 42 (34.1%) children reported allergies to both priority and non-priority foods, while 81 (65.9%) were allergic to priority allergens only. Amongst those with priority and non-priority allergy, there were various non-priority allergens reported, with legumes predominating. Notably, 11/42 (26.2%) reported pea allergy. Of those with priority allergies only, milk was consistently the most burdensome allergy across all domains. In contrast, amongst those with both priority and non-priority allergies, milk allergy was reported to be most burdensome within the domains of anxiety, stress, and expense, albeit at significantly lower frequencies than reported by those with priority allergies only (i.e. stress: 23.5% vs. 48.5%, respectively [p = 0.02]). Non-priority allergens were identified as the most burdensome within the domains of wish for tolerance (p = 0.02), worry about eating out (p = 0.04), and most burdensome overall (p = 0.02).

**Conclusions:** Caregivers of children with both priority and non-priority allergies report greater wish for tolerance, worry when eating out, and overall burden for non-priority allergens, but describe limited family anxiety and stress compared to those with priority allergies only.

### 27 Patient satisfaction with a virtual healthcare program for food allergen immunotherapy

#### Alexandra V. Baaske^1,2^, Edmond S. Chan^1,3^, Raymond Mak^1,3^, Tiffany Wong^1,3^, Kyla J. Hildebrand^1,3^, Stephanie Erdle^1,3^, Rishma Chooniedass^1,4^, Lianne Soller^1,3^

##### ^1^BC Children’s Hospital Research Institute, Vancouver, BC, Canada, ^2^Women’s Health Research Institute, Vancouver, BC, Canada, ^3^Division of Allergy & Immunology, Department of Pediatrics, Faculty of Medicine, University of British Columbia, Vancouver, BC, Canada, ^4^School of Nursing, Faculty of Health and Social Development, University of British Columbia, Kelowna, BC, Canada

###### **Correspondance:** Alexandra V. Baaske

*Allergy, Asthma & Clinical Immunology* 2022, **17(Suppl 1)**: 27

**Background:** The COVID-19 pandemic impacted allergy healthcare services by causing closure of clinics and movement towards virtual care. This shift occurred in our Food Immunotherapy Program. Our objective was to evaluate patient satisfaction of virtual Food Allergen Immunotherapy (FAIT), which has not been well characterized.

**Methods:** All families who received virtual FAIT between April 2021 and June 2021 were asked to complete a satisfaction survey online. Participants used Likert scales from 1 to 5 for satisfaction and perception of safety. Participants provided free-text responses about the benefits and risks of a virtual program. Participants selected preference for (1) In-person, longer wait time or (2) Virtual, shorter wait time. Median and Interquartile range were calculated for Likert scales. We scanned the open-ended responses for themes. Overall preference between in-person and virtual FAIT was measured with proportions.

**Results:** 34 families completed the survey. The median for satisfaction was 5 (IQR: 5, 5). The median for perception of safety was 4 (IQR: 4, 5). Eliminating cost and time for commuting to appointments was the most reported benefit. Delayed medical attention in the event of severe reaction emerged as the most common perceived risk. 29 (85.3%) respondents indicated that they preferred virtual immunotherapy treatment with a shorter wait time over in-person with a longer wait time.

**Conclusions:** Patient families are highly satisfied with a virtually supervised FAIT program, especially due to cost and time savings, and most feel it is safe. Most prefer virtual FAIT with a shorter wait time over in-person with a longer wait time. Comprehensive qualitative analysis is needed to better understand perceived risks and benefits. Data should be collected over a longer timeframe with a larger sample for continuous quality improvement and ensuring virtual FAIT is just as safe as an in-person program.

### 28 Differential levels of detection of peanut protein allergens under autoclaving conditions

#### Casey G. Cohen, Bertrand J. Jean-Claude, Bruce D. Mazer

##### McGill University, Montreal, QC, Canada

###### **Correspondance:** Casey G. Cohen

*Allergy, Asthma & Clinical Immunology* 2022, **17(Suppl 1)**: 28

**Background:** Peanut allergy is considered the most severe of all food allergies as it is the leading cause of fatal anaphylaxis. Evidence suggests that the allergenicity of peanuts is influenced by the method of peanut processing prior to consumption. The aim of this project is to develop alternative processing methods and evaluate their effect on the known peanut protein allergens and thus, peanut allergenicity.

**Methods:** Peanuts were ground into a paste and dissolved in hexanes for defatting. Protein extracts from raw, roasted (150 °C, 30 minutes), and autoclaved (136 °C, 2.5 atm, 30 minutes) peanuts were used to quantify relative levels of protein allergens Ara h 2 and Ara h 8 via Western blot analyses and ELISA using antibodies specific for each allergen.

**Results:** Similar levels of detection of Ara h 2 and Ara h 8 were observed in the raw and roasted peanut extracts as shown by Western blot and ELISA. Autoclaved extracts resulted in a decrease in Ara h 2 and Ara h 8 detection by approximately 50% and 100%, respectively, when compared to raw. The absence of signal in the Western Blot analysis indicates potential degradation of each allergen to different degrees in autoclaved peanut samples.

**Conclusions:** The results display discrete levels of detection of two peanut protein allergens following autoclaving. Given the distinct clinical reactions associated with specific IgE for Ara h 2 and Ara h 8, these findings have potential for higher power in peanut allergy diagnosis and treatment.

### 29 Pattern of peanut consumption and peanut sensitization at age 5 years in the CHILD Cohort Study

#### Samantha Lee^1^, Robert Balshaw^2^, Stuart E. Turvey^3^, Theo J. Moraes^4^, Piushkumar J. Mandhane^5^, Meghan B. Azad^6^, Padmaja Subbarao^4^, Elinor Simons^6^

##### ^1^Department of Biochemistry and Medical Genetics, University of Manitoba, Winnipeg, MB, Canada, ^2^Centre for Health Innovation, University of Manitoba, Winnipeg, MB, Canada, ^3^Department of Pediatrics, British Columbia’s Children’s Hospital, Vancouver, BC, Canada, ^4^Department of Pediatrics, Hospital for Sick Children, Toronto, ON, Canada, ^5^Division of Pediatric Respirology, Pulmonary & Asthma, University of Alberta, Edmonton, AB, Canada, ^6^Section of Allergy and Immunology, Department of Pediatrics and Child Health, University of Manitoba and Children’s Hospital Research Institute of Manitoba, Winnipeg, MB, Canada

###### **Correspondance:** Samantha Lee

*Allergy, Asthma & Clinical Immunology* 2022, **17(Suppl 1)**: 29

**Background:** Recommendations for childhood peanut allergy prevention emphasize early dietary peanut introduction and continuous peanut consumption based on studies in high-risk children. General-population CHILD Cohort Study children had lower peanut allergy and sensitization with peanut introduction by age 12 months; importance of continuous peanut consumption requires further study. We examined associations between continuous peanut consumption and peanut sensitization at age 5 years.

**Methods:** CHILD caregivers prospectively reported their child’s peanut consumption at ages 6, 9, 12, 18, 24, 30, 36, and 60 months. They were not advised how or when to introduce peanut. Continuous peanut consumption was defined as having no more than one gap in reported peanut consumption after first introduction before 18 months, and two or fewer gaps thereafter until 5 years. All other patterns were considered transient. Sensitization (positive skin prick testing [SPT] > 2 mm larger than the negative control) was measured at 1 and 5 years. Per current guidelines, children at high risk of peanut allergy had egg sensitization or allergy and/or moderate-to-severe atopic dermatitis in their first year. Multivariable logistic regression examined the odds of peanut sensitization at 5 years in continuous versus transient eaters.

**Results:** Of the 2440 children with SPT at 5 years, 2086 had complete peanut consumption data; 174 were high risk, 1800 consumed peanut continuously and 286 consumed transiently (including 189 who never consumed). Adjusting for maternal race, egg sensitization at 1 year, and study centre, children who consumed peanut continuously had reduced odds of peanut sensitization at age 5 years (OR 0.85, 95%CI: 0.83–0.86), even after excluding children who never consumed peanut (OR 0.88, 95%CI: 0.86–0.90) or after excluding high-risk children (OR 0.90, 95%CI: 0.88–0.91).

**Conclusions:** General-population children with continuous peanut consumption after first introduction had reduced odds of peanut sensitization at age 5 years, even after excluding high-risk children.

**Acknowledgement**: We thank the CHILD Cohort Study (CHILD) participant families for their dedication and commitment to advancing health research. CHILD was initially funded by CIHR and AllerGen NCE. Visit CHILD at childcohort.ca

### 30 Peanut, soy and non-priority allergy in Canada

#### Josie C. Cosyns^1^, Tara L. Frykas^1,2^, Hailey V. Hildebrand^1,2^, Harold Kim^3^, Jennifer D. Gerdts^4^, Elissa M. Abrams^1,2,5^, Jennifer L. Protudjer^2,6,9^

##### ^1^University of Manitoba, Winnipeg, MB, Canada, ^2^The Children’s Hospital Research Institute, Winnipeg, MB, Canada, ^3^Canadian Society of Allergy and Clinical Immunology, Orleans, ON, Canada, ^4^Food Allergy Canada, Toronto, ON, Canada, ^5^University of British Columbia, Vancouver, ON, Canada, ^6^Centre for Allergy Research, Stockholm, Sweden, ^7^Western University, London, ON, Canada, ^8^McMaster University, London, ON, Canada, ^9^George and Fay Yee Centre for Healthcare Innovation, Winnipeg, MB, Canada

###### **Correspondance:** Josie C. Cosyns

*Allergy, Asthma & Clinical Immunology* 2022, **17(Suppl 1)**: 30

**Background:** Peanut allergic individuals may be co-sensitized or co-allergic to another legume(s), although Canadian data are sparse. Our aim was to describe the distribution of legume allergy, specifically peanut and soy (priority allergens in Canada), and lentil, pea, chickpea, or other unspecific non-priority legumes in Canada, with consideration to age.

**Methods:** Caregivers of children (< 18 years) in Canada, with parent-reported allergies to at least one of the following: peanut, soy, lentil, pea, chickpea, or unspecific non-priority legumes, were included in this study population. Data were collected as part of two different online studies, between 2019 and 2021, approved by the University of Manitoba Health Research Ethics Board. Data were described, then analysed using logistic regression, and adjusted for sex, age at diagnosis and total number of food allergies, with statistical significance set at p < 0.05.

**Results:** Our study population included 115 children from all Canadian provinces, who were disproportionately boys (64.6%) and of which one-third were aged 6 or under. Nearly all (109/115; 94.8%) had peanut allergy, with lower prevalences of soy (18/115; 15.7%) and non-priority legumes (15/115; 13.0%). Most children had an epinephrine autoinjector (106/111; 95.5%) and had been diagnosed by an allergist (96/99; 98.0%). Specific to legume allergies, n = 85 children had mono-peanut allergy, n = 6 had mono-soy allergy, no children had non-priority legume allergy, n = 12 children had peanut+non-priority legume allergy, n = 9 had peanut+soy allergy, and n = 3 had peanut+soy+non-priority legume allergy. Compared to children aged 6 or under, older children were significantly less likely to have peanut, plus soy or nonpriority legume allergy (OR 0.22; 95%CI 0.05–0.94, p = 0.04).

**Conclusions:** In Canada, peanut allergy remains the most common legume allergy. However, allergy to peanut + at least one additional legume affects about 20% of children allergic to peanut, but disproportionately amongst young children.

### 31 Evolving EoE etiology: characterization of immediate and delayed allergic profiles

#### Tenzin Gyaltsen^1,2^, Jason Ohayon^1,2^

##### ^1^Hamilton Allergy, Hamilton, ON, Canada, ^2^McMaster University, Hamilton, ON, Canada

###### **Correspondance:** Tenzin Gyaltsen

*Allergy, Asthma & Clinical Immunology* 2022, **17(Suppl 1)**: 31

**Background:** Eosinophilic esophagitis (EoE) is diagnosed by biopsy-proven eosinophilic infiltration of the esophagus in excess of 15 eosinophils per high power field. Skin prick testing (SPT) and in some cases, food patch testing (FPT), are commonly performed to help direct allergen avoidance. Characterization of EoE’s atopic profile is variable.

**Methods:** A retrospective chart review was conducted at a community allergy clinic of patients referred for allergy assessment in biopsy-proven EoE. History of EoE symptoms, SPT IgE positivity to common foods and inhalants and FPT results were tracked. When available, follow-up endoscopic results were collected to determine trends in EoE phenotype outcomes.

**Results:** Fifty patients were identified with biopsy-proven EoE. SPT was positive in 34/50 (68%), with the majority to inhalants. FPT was positive in 35/48 (73%). Combined SPT and FPT were negative in 3/50 (6%). Twenty-three of 48 (48%) were combined FPT and SPT positive, 12/48 (25%) were FPT-positive and SPT-negative, 10/48 (21%) were FPT-negative and SPT-positive and 3/48 (6%) were FPT-negative and SPT-negative.

Twenty-five patients with EoE went on to a second endoscopy. Six patients displayed remission while 19 remained EoE-positive. Fourteen of the 19 (74%) were SPT-positive; 5/19 (26%) were SPT-negative. In addition, 15/19 (79%) were FPT-positive; 4/19 (21%) were FPT-negative.

**Conclusions:** The majority (96%) of confirmed EoE patients displayed either an individual or combined allergic profile based on results of SPT and FPT. However, individual SPT or FPT was positive in 68–75% of EoE patients. Allergy evaluation results in the first and follow-up endoscopes remained similar on biopsy. A combined allergic evaluation will yield greater results in the potential allergic avoidance role in the management of EoE. The impact of the above allergic evaluation will be explored in a separate abstract.

### 32 Canadian food ladders for dietary advancement in children with IgE-mediated allergy to milk and/or egg

#### Alanna Chomyn^1^, Edmond S. Chan^1^, Joanne Yeung^1^, Timothy K. Vander Leek^2^, Brock A. Williams^1^, Lianne Soller^1^, Elissa M. Abrams^3,1^, Raymond Mak^1^, Tiffany Wong^1^

##### ^1^University of British Columbia, Vancouver, BC, Canada, ^2^University of Alberta, Edmonton, AB, Canada, ^3^University of Manitoba, Winnipeg, MB, Canada

###### **Correspondance:** Alanna Chomyn

*Allergy, Asthma & Clinical Immunology* 2022, **17(Suppl 1)**: 32

**Background:** Milk and egg ladders, or “food ladders”, are tools designed to guide patients with IgE-mediated allergies through a stepwise introduction of increasingly allergenic forms of milk and egg in a home-based setting. Little data exists supporting food ladder use in clinical practice. This study aimed to adapt existing food ladders to reflect dietary practices in Canada, and determine safety of food ladders in Canada.

**Methods:** Canadian allergists determined patient suitability to receive a food ladder in clinical management of milk or egg allergy^1^. Inclusion criteria included patient age < 18 years and a diagnosis of egg or milk allergy. Patients were provided with a survey link along with the relevant food ladder(s). Parents completed baseline and follow up surveys at 3 and 6 months. Descriptive statistics were obtained through REDCap.

**Results:** Between January – June 2021, ten 3-month follow up surveys and five 6-month follow up surveys were completed; 9 (90%) patients were still using food ladders at 3-month follow up, and one had stopped due to unclear reasons. All 5 patients who completed follow up surveys at 6 months were still using food ladders. Three patients (30%; 2 on egg ladder, 1 on milk ladder), experienced minor skin or gastrointestinal tract symptoms. None required epinephrine or emergency department visits. Eight (89%) parents felt comfortable managing ladder progression at home, and 1 (12.5%) parent felt unsure.

**Conclusions:** Preliminary data suggest food ladders may be a feasible, safe method for home-based dietary expansion in select milk and/or egg allergic children with mild IgE-mediated symptoms. Further evaluation is needed to confirm safety of food ladders.

**References:**

1. Submitted for publication

### 33 Evaluating EoE endpoints: treatments and allergic profiling in the determination of clinical outcomes

#### Jason Ohayon^1,2^, Tenzin Gyaltsen^1,2^

##### ^1^Hamilton Allergy, Hamilton, ON, Canada, ^2^McMaster University, Hamilton, ON, Canada

###### **Correspondance:** Tenzin Gyaltsen

*Allergy, Asthma & Clinical Immunology* 2022, **17(Suppl 1)**: 33

**Background:** Eosinophilic esophagitis (EoE) is diagnosed by biopsy-proven esophageal eosinophil infiltration. It is characterized by a variable allergic profile, with skin-prick testing (SPT) and food-patch testing (FPT) commonly conducted to guide allergen avoidance. Proton-pump inhibitors (PPIs) or budesonide may also be considered to manage symptoms.

**Methods:** A retrospective chart review was conducted at a community allergy clinic of patients referred for biopsy-proven EoE. History, SPT IgE-positivity, FPT results and treatments were tracked. When available, follow-up endoscopic and consultation results were collected to determine patterns in EoE outcomes.

**Results:** In 50 EoE patients, PPI treatment/avoidance was indicated in 17/50 (34%) while an avoidance diet alone was indicated in 31/50 (62%). 

Twenty-five patients underwent a second endoscopy. Six patients displayed remission (1 with treatment plan unavailable), with 1/5 (20%) on PPI/avoidance and 4/5 (80%) on avoidance alone. SPT-positivity was 67% while FPT-positivity was 83%. Nineteen remained EoE-positive, with 7/19 (37%) on PPI/avoidance and 12/19 (63%) on avoidance alone. SPT-positivity was 74% while FPT-positivity was 79%.

If not follow-up scoped, follow-up data was available for 14 patients. Eleven patients displayed clinical improvement/control, with 4/11 (36%) on PPI/avoidance and 7/11 (64%) on avoidance diet alone. SPT-positivity was 82% and FPT-positivity was 64%. Three remained symptomatic, and on avoidance diet alone. SPT-positivity was 67% and FPT-positivity was 33%.

**Conclusions:** The majority of patients practiced an avoidance diet alone with no PPI. Patients upon second endoscopy, both EoE-positive and negative, had been treated similarly. Allergic profiles were also similar across EoE-positive and negative patients. Upon follow-up consultation, clinically-improved and symptomatic patients had similar treatments and allergic profiles. These data suggest a reduced role for treatment and allergic profile in EoE improvement/control versus exacerbation, although it must be considered that clinically-improved patients may not seek/attend follow-up consultations/endoscopies.

## Immunology

### 34 COVID-19 vaccine related concerns and reactions: a retrospective chart review

#### Anna M. De Jong^1,2^, Stephanie G. Brooks^1,3^, Madeleine E. Smee^1^, Mina Abbaslou^1^, Gordon Sussman^1,4^

##### ^1^Gordon Sussman Clinical Research Inc., North York, ON, Canada, ^2^Faculty of Science, University of Western Ontario, London, ON, Canada, ^3^Temerty Faculty of Medicine, University of Toronto, Toronto, ON, Canada, ^4^Department of Medicine and Division of Clinical Immunology & Allergy, University of Toronto, Toronto, ON, Canada

###### **Correspondance:** Anna M. De Jong

*Allergy, Asthma & Clinical Immunology* 2022, **17(Suppl 1)**: 34

**Background:** The COVID-19 pandemic has increased levels of anxiety for many Canadians. However, patients with known allergies/immunological conditions have been particularly anxious regarding COVID-19 and related vaccinations. The purpose of this study is to describe patients with COVID-19 vaccine concerns who did not experience any reactions and explore the reactions that did occur.

**Methods:** A retrospective chart review was performed for patients seen at an Allergy and Immunology clinic in Toronto, Ontario between December 2020 and June 2021. Patients were included if they spoke with staff regarding COVID-19 vaccine concerns or reported a reaction to one of the vaccines.

**Results:** There were 41 patients (53.7% female) concerned about the COVID-19 vaccine who had no reaction. Approximately half received one dose (n = 19, 46.3%) of the vaccine. The mRNA vaccines were the most common (Pfizer-BioNTech n = 28, 68.3%; Moderna n = 4, 9.8%; not reported n = 6, 14.6%). Most common reasons for concern included history of urticaria (n = 11, 26.8%), adverse reaction to a vaccine or drug (n = 10, 24.4%), anaphylaxis (n = 7, 17.1%) or known polyethylene glycol allergy (n = 5, 12.2%). For those who experienced a vaccine reaction (n = 33, 90.9% female), 26 (83.9%) had a known allergy(ies) or immunological condition. The majority received the Pfizer-BioNTech vaccine (n = 26, 78.8%), followed by Moderna (n = 5, 15.2%) and AstraZeneca (n = 2, 6.1%). Approximately half of patients received one dose (n = 14, 42.4%; not reported n = 5, 15.2%). The most common reactions were urticaria (n = 12, 36.4%), paresthesia (n = 6, 18.2%), “COVID arm” (n = 4, 12.1%) and other cutaneous manifestations (n = 4, 12.1%). Majority of patients reported complete resolution of symptoms within a few days to weeks (n = 26, 78.8%), while seven (21.1%) have ongoing symptoms.

**Conclusions:** From preliminary data, only 33 patients reacted to a COVID-19 vaccine. Although vaccine hesitancy is prevalent and risk of reaction is low, patients with allergy(ies)/immunological conditions may benefit from an allergist/immunologist consultation prior to vaccination.

### 35 Antibodies to dense fine speckled 70 autoantigen (DFS70) predicts good outcome in mechanically ventilated COVID-19 patients

#### Rameen Jamil^1,2^, Kiho Son^1,2^, Allison Kennedy^3^, Tim Xiaotian Jua^1^, Braeden Cowbrough^3^, Sarah Culgin^2^, Melanie Kjarsgaard^1,2^, Katherine Radford^1,2^, Dawn Bowdish^1,3^, Roma Sehmi^1^, Parameswaran Nair^1,2^, Dan Perri^1,2^, Manali Mukherjee^1,2,3^

##### ^1^Department of Medicine, McMaster University, Hamilton, ON, Canada, ^2^The Research Institute of St. Joe’s. St Joseph’s Healthcare Hamilton, Hamilton, ON, Canada, ^3^McMaster Immunology Research Centre, Faculty of Health Sciences, Hamilton, ON, Canada

###### **Correspondance:** Rameen Jamil

*Allergy, Asthma & Clinical Immunology* 2022, **17(Suppl 1)**: 35

**Background:** Multiple factors (age, male sex, lymphopenia, co-morbidities) are associated with the severity of Coronavirus disease-2019 (COVID-19). Evidence of circulating autoantibodies [1] and neutrophil extracellular trap (NETs) [2] during acute phase of the infection indicate a possible autoimmune pathomechanism. We investigated if autoimmune markers in critically ill COVID-19 patients predict fatality

**Methods:** In a single-center, prospective, multiple time-point observational study with mechanically ventilated COVID-19 patients, blood and endotracheal aspirates (ETA) were collected at the time of intubation, at worsening (and/or 7 days), and at extubation. Seventeen common autoantibodies associated with clinical pathologies, and anti-DFS70 (dense fine speckled 70, an exclusion marker for rheumatologic disease), were detected using anti-nuclear/extra-nuclear antibody line immunoassay (IMTEC-ANA-LIA XL, Human Diagnostics, Germany).

**Results:** Of our interim analysis with 22 patients who completed the study, 10 had a fatal outcome (45%). Demographics/clinical parameters were unremarkable between survivor and fatal sub-groups. At 1:100 titer, 68% (15) patients had > 2 circulating autoreactivities on the ANA panel. Mortality was associated with increased dsDNA and NETs in serum and endotracheal aspirates, and low levels of circulating T-regulatory cells (P<0.05). However, presence of anti-DFS70 predicted good outcome (Odd ratio:41.3, P = 0.04) irrespective of other autoreactivities (Kaplan Meier, Log-rank test, P = 0.003).

**Conclusions:** To our knowledge, this is the first study to analyze markers of autoimmunity in ventilated patients to predict mortality. The data provides a strong rationale to develop anti-DFS70 as a biomarker as well as investigate their protective mechanism against an autoimmune pathology.

**References:**

1. Wang Y, Mao T, Klein J. et al: Diverse functional autoantibodies in patients with COVID-19. Nature. 2021.
2. Zuo Y, Yalavarthi S, Shi H, et al: Neutrophil extracellular traps in COVID-19. JCI Insight. 2020; 5(11).

### 36 Exploratory safety analysis of oral immunotherapy across the pediatric age spectrum in a community based allergy clinic

#### Duva Karunakaran^1,2^, Rosemary Invik^3^, Lianne Soller^1,4^, Victoria E. Cook^3,4^, Edmond S. Chan^1,4^, Scott B. Cameron^3,4^

##### ^1^Allergy and Immunology, BC Children’s Hospital, Vancouver, BC, Canada, ^2^Department of Medicine, Queen’s University, Kingston, ON, Canada, ^3^Community Allergy Clinic, Victoria, BC, Canada, ^4^Division of Allergy and Immunology, Department of Pediatrics, University of British Columbia, Vancouver, BC, Canada

###### **Correspondance:** Duva Karunakaran

*Allergy, Asthma & Clinical Immunology* 2022, **17(Suppl 1)**: 36

**Background:** Real-world safety of oral immunotherapy (OIT) across the pediatric age spectrum has not been well-studied. This is an exploratory analysis of the association between grade-2+ reactions and OIT when administered to children < 4 years old as compared to ≥4 years.

**Methods:** OIT was administered through a community practice in Victoria, BC in children aged 0–18 who fulfilled previously defined inclusion criteria [1]. Patients underwent OIT buildup to 1–10 foods in clinic over the course of several weeks, to achieve a maintenance dose of 300mg per food. Symptoms of reactions were tracked and patients with grade-2+ [1] reactions were compared according to age at OIT start using a chi-squared test. As a secondary outcome, linear regression modelling was performed to determine the relationship between number of foods treated and grade-2+ reactions.

**Results:** Between 2017 and 2021, 186 patients (median age: 5) started OIT, 23 (12.4%) of which had grade-2+ reactions during build-up. These reactions were experienced by 9% of patients < 4 years and 15% in ≥ 4 years (p = 0.48). The linear model found that incidence of grade-2+ reactions increased by 7.9% per food (R2 = 0.8551).

**Conclusions:** Although preliminary, our data suggests that number of foods treated increases the risk of grade-2+ reactions during OIT buildup. Further research is needed to determine whether there is some association between age and increasing number of foods treated, or whether a delay between initial diagnosis and start of OIT may play a role in this increased risk. Additionally, factors such as starting dose, history of previous reactions, food combinations of concurrent OIT, and other factors should be further explored to improve safety outcomes for all patients.

**References:**

1. Soller L et al. First real-world safety analysis of preschool peanut oral immunotherapy. The Journal of Allergy and Clinical Immunology: In Practice. 2019 Nov 1;7(8):2759–67.

### 37 Determining the impact of IL-5 on human mast cell responses to viral infections

#### Christopher R. Liwski, Ian Haidl, Liliana Portales-Cervantes, Jean S. Marshall

##### Dalhousie University, Department of Microbiology and Immunology, Halifax, NS, Canada

###### **Correspondance:** Christopher R. Liwski

*Allergy, Asthma & Clinical Immunology* 2022, **17(Suppl 1)**: 37

**Background:** Mast cells are tissue-resident sentinels that regulate responses to pathogens and allergens. They are critical for hypersensitivity reactions and typically activated in Th2 cytokine-rich environments during allergic disease. Mast cells respond to viruses by producing chemokines and type I and III interferons (IFNs). Clinical studies have investigated Th2 cytokine inhibitors, including IL-5 inhibitors, in asthma therapy^1–3^. However, the influence of IL-5 inhibition on mast cell responses to virus infection remains unclear. The effects of IL-5 or IL-5 inhibition on mast cell antiviral responses may be important in virus-associated exacerbation of asthma.

**Methods:** Human cord-blood-derived mast cells from anonymized donors (n = 14; 12 donors) were isolated and pre-treated in culture medium with or without 10 ng/mL IL-5 for 48 hours. Cells were infected with OC43 coronavirus at 1.0 MOI, treated with poly(I:C), or left in culture medium (mock) for 24 hours. qPCR and Luminex analysis were performed to assess mRNA and protein levels for type I and III IFNs*. *Statistical analysis was performed using mixed-effects analysis with Sidak’s multiple comparisons test.

**Results:** Coronavirus infection or poly(I:C) treatment of mast cells resulted in significantly higher levels of *IFNA2*, *IFNB1*, and *IFNL1* mRNA compared to respective mock treatments (p < 0.01–0.0001). Luminex analysis revealed that IFNα2 production was increased in IL-5-pre-treated and non-pre-treated cells following poly(I:C) stimulation and following coronavirus infection of IL-5-pre-treated cells compared to controls (p < 0.001–0.0001). Interestingly, the enhancement of mRNA and protein expression following coronavirus infection or poly(I:C) treatment was significantly greater in IL-5-pre-treated compared to non-pre-treated mast cells for all targets (p < 0.05–0.001).

**Conclusions:** IL-5-pre-treatment enhances the expression of type I and III IFN mRNAs by mast cells in response to coronavirus or poly(I:C), suggesting IL-5 may have an important role in mast cell antiviral responses. The mechanism by which IL-5 enhances mast cell IFN expression will be investigated further.

**References:**

1. Farne HA, Wilson A, Powell C, Bax L, Milan SJ. Anti-IL5 therapies for asthma. Cochrane Database Syst Rev 2017; 9: CD010834.
2. Sabogal Piñeros YS, Bal SM, van de Pol MA, Dierdorp BS, Dekker T, Dijkhuis A, Brinkman P, van der Sluijs KF, Zwinderman AH, Majoor CJ, Bonta PI, Ravanetti L, Sterk PJ, Lutter R. Anti-IL-5 in mild asthma alters rhinovirus-induced macrophage, B-cell, and neutrophil responses (MATERIAL). A placebo-controlled, double-blind study. Am J Respir Crit Care Med 2019; 199(4): 508–517.
3. Contoli M, Papi A. Effects of anti-IL-5 on virus-induced exacerbation in asthma. Light and shadow. Am J Respir Crit Care Med 2019; 199(4): 410–411.

### 38 Developing analytical confocal microscopy tools to study allergic disease

#### Jake Colautti, Joshua Koenig, Manel Jordana

##### McMaster University, Hamilton, ON, Canada

###### **Correspondance:** Jake Colautti

*Allergy, Asthma & Clinical Immunology* 2022, **17(Suppl 1)**: 38

**Background:** Interactions between immune cells are critical for their activation and effector function. Many immune cells reside within niches in the tissue microenvironment which are critical for their survival, proliferation, and activity. Traditional methods such as flow cytometry remove cells from their tissue environment for measurement at the single-cell level, and therefore capture neither cell-cell interactions nor cellular niches. This is typically overcome using immunofluorescence microscopy, which allows for *in situ* analysis but fails to capture heterogenous phenotypes as it is limited to the visualization of 7–8 markers simultaneously. IBEX (iterative bleaching extends multiplexity), an approach in which a specimen is stained with fluorescent antibodies, imaged, bleached, re-stained, and re-imaged, allows for visualization of a theoretically unlimited number of markers, potentiating highly multiplexed quantitative analysis of cellar phenotype, interactions, and tissue microenvironment.^1^

**Objective:** Develop an IBEX multiplexed imaging panel to study the cells and molecules associated with food allergy.

**Methods:** C57/Bl6 mice were immunized with ovalbumin (OVA) and alum intraperitoneally. Tissues were harvested, fixed, frozen, and sectioned for analysis. A panel of 60 antibodies was optimized for imaging. Images were captured using a Leica Stellaris 5 confocal microscope and analyzed with Ilastik and histoCAT.^1,2^

**Results:** IBEX reliably detects major immune cell populations over several staining cycles. Staining immunized mesenteric lymph nodes with OVA-AF647 allowed detection of allergen-specific B cells (B220^+^OVA^+^). Lastly, staining for immunoglobulin allows visualization of B cell isotype, which is validated by the absence of IgG1 and IgE signal in IgG1 and IgE deficient mice, respectively.

**Conclusions:** These results indicate that IBEX can be used to study major immune cell populations, B cell isotype, and allergen specificity in mouse models of food allergy. The use of this tool in allergic model systems will help better understand the spatial distribution of immune cells and potentially identify novel therapeutic targets.

**References:**

1. Radtke AJ, Kandov E, Lowekamp B, et al. IBEX: A versatile multiplex optical imaging approach for deep phenotyping and spatial analysis of cells in complex tissues. *Proc Natl Acad Sci*. 2020;117(52):33455. doi:10.1073/pnas.2018488117
2. Schapiro D, Jackson HW, Raghuraman S, et al. histoCAT: analysis of cell phenotypes and interactions in multiplex image cytometry data. *Nat Methods*. 2017;14(9):873–876. doi:10.1038/nmeth.4391

### 39 Changes in mental status associated with hypereosinophilia

#### Peter Anto Johnson, John Christy Johnson, Austin Mardon

##### University of Alberta, Edmonton, AB, Canada

###### **Correspondance:** Peter Anto Johnson

*Allergy, Asthma & Clinical Immunology* 2022, **17(Suppl 1)**: 39

**Background:** Hypereosinophilia is a rare but significant condition, wherein eosinophils infiltrate cells, release inflammatory cytokines, and may lead to systemic organ and damage. It could precipitated by allergic diseases, infection, or hypersensitivity reactions and lead to rapid changes in mental status. However, the mechanism of central nervous system-related changes in patients with hypereosinophilic syndrome is still poorly understood. We aimed to provide a descriptive overview to synthesize current evidence in the literature.

**Methods:** We conducted a review of the literature followed by a qualitative narrative synthesis following ENTREQ guidelines. Databases including PubMed/MEDLINE, EMBASE and Google Scholar were screened, and no time, setting, or language restrictions were imposed on the search strategy. Keywords in our search included: “eosinophilia”, “mental”, “consciousness”, “psychological”, “neurologic”, “nervous system” and “HES”. Primary research articles such as case studies, systematic reviews and meta-analyses, were included. Experimental and animal studies were excluded.

**Results:** We identified 14 articles that met our inclusion criteria. Of the 14, ten discussed the presence of mental changes including stroke, temporal arteritis, leptomeningeal dissemination, memory deficits and dysarthria associated with hypereosinophilia. Three were systematic reviews identifying 143 cases and evaluating outcomes and discussing risk factors. Two major mechanisms including encephalopathy, which can result in states of confusion, and multiple embolic strokes and/or an increased presence of ischemic lesions in the brain accumulated the greatest mentions in literature. Cerebrovascular disease was an important co-morbidity as well as a significant risk factor characterized or mentioned by 12 studies.

**Conclusions:** The evidence on changes in mental status in patients with hypereosinophilic syndrome is currently limited and of low quality involving retrospective studies, case studies, and reviews; thus, more rigorous studies are warranted.

### 40 Update on the diagnosis of primary immunodeficiencies with comprehensive genetic testing by next-generation sequencing

#### Christine Davies^1^, Zöe Powis^1^, Alicia Scocchia^1^, Päivi Kokkonen^2^, Elina Hirvonen^2^, Emma Mårtensson^2^, Hatice Duzkale^2^, Kim Gall^1^, Inka Saarinen^2^, Johanna Sistonen^2^, Samuel Myllynkangas^2^, Juha Koskenvuo^2^, Lotta Koskinen^2^, Tero-Pekka Alastalo^1^

##### ^1^Blueprint Genetics, Seattle, WA, USA, ^2^Blueprint Genetics, Espoo, Finland

###### **Correspondance:** Christine Davies

*Allergy, Asthma & Clinical Immunology* 2022, **17(Suppl 1)**: 40

**Background:** Historically, genetic testing for patients with suspected primary immunodeficiency disorder (PIDs) was done following clinical diagnosis. Genetic testing is now part of the diagnostic evaluation for PIDs given phenotypic heterogeneity, management, familial donor and genetic counselling implications. Here, we update the experience with PID multigene panels at a CLIA-certified diagnostic laboratory.

**Methods:** An update of our previous retrospective review was undertaken. Testing included 11 next-generation sequencing (NGS) PID panels with sequence, high-resolution copy number (CNV), and targeted non-coding variant analysis. CNV analysis was performed bioinformatically from NGS data using 2 variant calling algorithms, including one for small, intragenic, exon-level CNVs. Variant interpretation used an adaptation of the American College of Medical Genetics and Genomics guidelines. A molecular diagnosis occurred upon identifying a pathogenic or likely pathogenic variant consistent with phenotype and known associated disease inheritance.

**Results:** Results from 3,915 patients were analyzed. The median age at testing was 14 years. A molecular diagnosis was established in 12.8% (503) patients. Significantly, more patients < 1 year of age received molecular diagnoses (25.3%, P < 0.0001). Patients 51–60 (7.0%, P = 0.0006) and 61+ years of age (4.7%, P < 0.0001) were significantly less likely to receive molecular diagnoses. The chronic granulomatous disease (19/39, 48.7%) and severe combined immunodeficiency (13/40, 32.5%) panels had the highest diagnostic yields. The most common diagnoses were MEFV-associated familial Mediterranean fever (29, 5.8%) and ELANE-associated neutropenia (21, 4.2%). Unique genes accounted for 70 diagnoses (13.9%). Intronic variants 12.1% (61) and CNVs 12.5% (63) contributed frequently to diagnoses.

**Conclusions:** Young patients with PIDs are more likely to receive a molecular diagnosis. Nearly 25% of diagnostic variants were CNVs or intronic variants, which may be difficult-to-detect, or not included, depending on test capabilities and target regions. Genetic testing by NGS panels that includes high-resolution CNV and intronic variant analysis contribute to increased diagnostic yield and thus potentially improved care.

### 41 Dupilumab improves signs and symptoms of severe atopic dermatitis in children aged 6–11 years with and without history of comorbid asthma

#### Thomas Bieber^1^, Mark Boguniewicz^2^, Elaine C. Siegfried^3^, Zhen Chen^4^, Randy Prescilla^5^, Brad Shumel^4^

##### ^1^University Hospital of Bonn, Bonn, Germany, ^2^National Jewish Health and University of Colorado School of Medicine, Denver, CO, USA, ^3^Saint Louis University and Cardinal Glennon Children’s Hospital, St. Louis, MO, USA, ^4^Regeneron Pharmaceuticals, Inc., Tarrytown, NY, USA, ^5^Sanofi Genzyme, Cambridge, MA, USA

###### **Correspondance:** Mark Boguniewicz

*Allergy, Asthma & Clinical Immunology* 2022, **17(Suppl 1)**: 41

**Background:** Atopic dermatitis (AD) frequently presents with atopic comorbidities, including asthma. Dupilumab inhibits interleukin 4 and 13 signaling and is approved for treating multiple type 2 inflammatory diseases, including AD and asthma. We evaluated the efficacy of dupilumab with concomitant topical corticosteroids (TCS) for severe AD in children with and without history of comorbid asthma (h-asthma).

**Methods:** In this global double-blind, 16-week, phase 3 LIBERTY AD PEDS trial (NCT03345914), 367 children aged 6–11 years were randomized 1:1:1 to 300mg dupilumab every 4 weeks (q4w), weight-based 100/200mg dupilumab every 2 weeks (q2w); or placebo; with concomitant medium-potency TCS. Asthma history was reported by the caregiver.

**Results:** At baseline, 48%/52%/49% patients in the q4w/q2w/placebo groups reported h-asthma. Baseline q4w/q2w vs placebo Peak Pruritus Numerical Rating Scale scores (PP-NRS; with h-asthma: 7.9/7.9 vs 7.9; without h-asthma: 7.7/7.7 vs 7.6) and Eczema Area and Severity Index (EASI; with h-asthma: 36.7/38.7 vs 40.9; without h-asthma: 38.0/35.8 vs 37.2) were comparable in both patient subgroups. At Week 16, in both subgroups, significantly more patients receiving dupilumab q4w/q2w vs placebo achieved Investigator’s Global Assessment score 0/1 (with h-asthma: 37.9%/27.0% vs 11.7%; without h-asthma: 28.1%/32.2% vs 11.1%); ≥75% improvement in EASI (with h-asthma: 65.5%/58.7% vs 25.0%; without h-asthma: 73.4%/76.3% vs 28.6%); and ≥ 4-point reduction from baseline in PP-NRS (with h-asthma: 48.3%/50.8% vs 8.5%; without h-asthma: 53.2%/66.1% vs 15.9%); *P*<0.05 for all comparisons. Least squares mean change from baseline in EASI was: with h-asthma: − 80.1%/− 75.7% vs − 50.3%; without h-asthma: − 83.1%/− 80.5% vs − 45.9%; *P* <  0.05. Safety in the overall population was acceptable and consistent with the known dupilumab safety profile observed in adults and adolescents.

**Conclusions:** Dupilumab with concomitant TCS was equally efficacious vs placebo in children aged 6–11 years with severe AD with and without a history of comorbid asthma in improving signs and symptoms of AD.

### 42 Dupilumab improves signs and symptoms of severe atopic dermatitis in children aged 6–11 years with and without comorbid allergic rhinitis

#### Andreas Wollenberg^1^, Elaine C. Siegfried^2^, Mark Boguniewicz^3^, Zhen Chen^4^, Alvina Abramova^4^, Randy Prescilla^5^

##### ^1^Ludwig-Maximilian University, Munich, Germany, ^2^Saint Louis University and Cardinal Glennon Children’s Hospital, St. Louis, MO, USA, ^3^National Jewish Health and University of Colorado School of Medicine, Denver, CO, USA, 4. Regeneron Pharmaceuticals, Inc., Tarrytown, NY, USA, 5. Sanofi Genzyme, Cambridge, MA, USA

###### **Correspondance:** Andreas Wollenberg

*Allergy, Asthma & Clinical Immunology* 2022, **17(Suppl 1)**: 42

**Background:** Atopic dermatitis (AD) is a chronic inflammatory systemic disease that frequently is presented with atopic comorbidities, including allergic rhinitis (AR). Dupilumab inhibits signaling of interleukin-4 and interleukin-13, key and central drivers of type 2-mediated inflammation in multiple diseases, including AD and AR. This subgroup analysis evaluated the efficacy of dupilumab with concomitant topical corticosteroids (TCS) for severe AD in children with and without comorbid AR.

**Methods:** In the double-blind, 16-week, phase 3 LIBERTY AD PEDS trial (NCT03345914), 367 children aged 6–11 years with severe AD were randomized 1:1:1 to 300 mg dupilumab every 4 weeks (q4w); weight-based 100/200 mg dupilumab every 2 weeks (q2w); or placebo; with concomitant medium-potency TCS. AR history was reported by the caregiver.

**Results:** At baseline, 75/75/77 patients in the q4w/q2w/placebo groups reported a history of AR; 47/47/46 patients had no AR history. Baseline disease severity was comparable in both subgroups. At Week 16, more patients receiving dupilumab q4w/q2w vs placebo achieved Investigator’s Global Assessment score 0/1 (with AR: 32.0%/30.7% vs 9.1%; without AR: 34.0%/27.7% vs 15.2%); ≥ 75% improvement in Eczema Area and Severity Index (EASI-75; with AR: 68.0%/69.3% vs 20.8%; without AR: 72.3%/63.8% vs 37.0%); and ≥ 3-point reduction from baseline in Peak Pruritus Numerical Rating Scale score (with AR: 56.8%/65.8% vs 24.7%; without AR: 66.0%/70.2% vs 15.2%) (*P*<0.05 for all comparisons except q2w vs placebo for IGA in patients without AR [not statistically significant]). Least squares mean change from baseline in EASI was − 82.9%/− 79.7% vs − 46.5% and − 80.6%/− 76.6% vs − 51.8% in patients with AR and without AR, respectively; *P* < 0.05). Safety in the overall population was consistent with the known dupilumab safety profile in adults and adolescents.

**Conclusions:** Dupilumab with concomitant TCS improved signs and symptoms of AD in children aged 6–11 years with severe AD, with and without comorbid AR.

### 43 Long-term efficacy and safety of dupilumab in a phase 3, open-label extension trial in patients aged ≥6 to <12 years with uncontrolled, moderate-to-severe atopic dermatitis

#### Michael J. Cork^1^, Lawrence Eichenfield^2^, Zhen Chen^3^, Dimittri Delevry^3^, John T. O’Malley^4^, Ashish Bansal^3^

##### ^1^Sheffield Dermatology Research, University of Sheffield, Sheffield, United Kingdom, ^2^University of California and Rady Children’s Hospital, San Diego, CA, USA, ^3^Regeneron Pharmaceuticals, Inc., Tarrytown, NY, USA, ^4^Sanofi Genzyme, Cambridge, MA, USA

###### **Correspondance:** Michael J. Cork

*Allergy, Asthma & Clinical Immunology* 2022, **17(Suppl 1)**: 43

**Background:** Long-term treatment options of atopic dermatitis (AD) in children are limited. We report efficacy and safety data from the ongoing, long-term LIBERTY AD PED-OLE trial of dupilumab (NCT02612454).

**Methods:** Patients aged ≥ 6 months to < 18 years with moderate-to-severe AD who had participated in a previous dupilumab study were enrolled in this OLE study. Data reported here include patients aged ≥ 6 years to < 12 years (n = 362 at OLE baseline, n = 309 at Wk 4, n = 34 at Wk 52; data cutoff: July 22, 2019).

Patients received 300 mg every 4 weeks (q4w), which could be up-titrated in case of inadequate clinical response at Week (Wk) 16 as: patients < 60 kg—200 mg q2w; patients ≥ 60 kg—300 mg q2w.

**Results:** 18% of patients had an Investigator’s Global Assessment score of 0/1 at OLE baseline, 24.6% at Wk 4, 37.8% at Wk 28, and 44.1% at Wk52. Mean percent change (standard deviation) from parent study baseline (PSBL) to OLE baseline in Eczema Area and Severity Index (EASI) was − 59.4(36.4), with incremental improvement at Wk 4 (− 71.1 [26.2]), Wk 28 (− 81.8 [17.9]), and Wk 52 (− 85.7 [17.5]). At OLE baseline, 41.2% of patients achieved ≥ 75% reduction in EASI relative to PSBL, increasing to 54.4% at Wk 4, 72.4% at Wk 28 and 79.4% at Wk 52. Treatment-emergent adverse events (TEAEs) were reported in 58.8% of patients; 2.5% of patients had a serious TEAE. Most common TEAEs were AD exacerbation (15.5%) and nasopharyngitis (13.0%).

**Conclusions:** Long-term treatment with dupilumab showed sustained improvement in signs of AD in the cohort of patients who completed up to 52 weeks. Safety data were consistent with the known dupilumab safety profile.

### 45 Evaluation of rotavirus vaccine adherence for the 22q11.2DS patient population

#### Sophie McGregor^1^, Matthew Boroditsky^1^, Geraldine Blanchard-Rohner^2^, Kyla J. Hildebrand^3,4^

##### ^1^University of British Columbia, Medical Undergraduate Program, Vancouver, BC, Canada, ^2^Unit of Immunology and Vaccinology, Division of General Pediatrics, Department of Pediatrics, Gynecology and Obstetrics, Geneva University Hospitals, University of Geneva, Geneva, Switzerland, ^3^British Columbia Children’s Hospital, Vancouver, BC, Canada; Division of Allergy and Immunology, Department of Pediatrics, Faculty of Medicine, University of British Columbia, Vancouver, BC, Canada, ^4^British Columbia Children’s Hospital Research Institute, Vancouver, BC, Canada

###### **Correspondance:** Sophie McGregor

*Allergy, Asthma & Clinical Immunology* 2022, **17(Suppl 1)**: 45

**Background:** 22q11.2 Deletion Syndrome (22q11.2DS) can result in congenital abnormalities including immune dysfunction. International guidelines recommend immune evaluation of 22q11.2DS patients prior to administration of live vaccines [1]. A rotavirus immunization program for infants aged 2 and 4 months was implemented in British Columbia (BC) in January 2012 [2]. Prior to the Rotavirus vaccine, the first live vaccine was administered at 12 months. Adherence to immune workup recommendations prior to 2 months of age in patients with 22q11.2DS and adverse events following immunization is not known.

**Methods:** A retrospective chart review of children diagnosed with 22q11.2DS in BC from January 1, 2012 to January 1, 2019 was conducted. Demographic, clinical, laboratory, and immunization data and adverse reactions to vaccines were obtained from hospital records. International pediatric consensus guidelines were used as a reference to determine adherence to guidelines for immunologic workup [1]. Institutional research ethics board approval was obtained.

**Results:** Records of forty-two children with 22q11.2DS were reviewed and 39 children had immunization records available. Twenty-two out of 39 (56.4%) received at least one dose of a live attenuated rotavirus vaccine. No adverse events following immunization were noted. Ten (25.6%) infants received an immunological assessment prior to rotavirus vaccine administration, and six out of the ten (15.3%) had a CD4+ lymphocyte count higher than the cut-off of 500 × 10^6^cells/L to qualify for safe administration of a live attenuated vaccination yet did not receive the Rotavirus vaccine [3].

**Conclusions:** In this cohort of patients with 22q11 DS, the majority did not receive immunological workup consistent with international guidelines for immunization, though no adverse immunization events were identified. Further assessment is warranted to determine what immunological workup is needed prior to Rotavirus vaccine. Greater dissemination of 22q11.2DS guidelines and improved infant screening for 22q11.2DS in BC is recommended.

**References:**

1. Bassett AS, McDonald-McGinn DM, Devriendt K, Digilio MC, Goldenberg P, Habel A, et al. Practical Guidelines for Managing Patients with 22q12 Deletion Syndrome. J Pediatr 2011;159:332–339.e
2. Naus, M. Transition to a three-dose rotavirus vaccine in BC. BCMJ 2018 60:223–224
3. Canada PHA. Rotavirus vaccine: Canadian Immunization Guide 2017 [cited 2021 Jun 24] Available from: https://www.canada.ca/en/public-health/services/publications/healthy-living/canadian-immunization-guide-part-4-active-vaccines/page-19-rotavirus-vaccine.html#p4c18a6.

### 46 Hereditary angioedema in Canada: Changes in medication use and untreated attacks between the 2017 and 2020 surveys

#### Jacquie Badiou^2^, Michelle Cooper^2^, Daphne Dumbrille^2^, Robert Bick^4^, Maggie Dao^1^, Suzanne M. Kelly^1^, Gina Lacuesta^3^, Pauk Keith^5^, Amin Kanini^6^, Martine Paquette^2^, William H. Yang^7^

##### ^1^Red Maple Trials, Inc., Ottawa, ON, Canada, ^2^HAE Canada, Ottawa, ON, Canada, ^3^Dalhouse University, Halifax, NS, Canada, ^4^Health Policy Consultant, Markham, ON, Canada, ^5^MacMaster University, Hamilton, ON, Canada, ^6^University of British Columbia, Vancouver, BC, Canada, ^7^Ottawa Allergy Research Corporation, Ottawa, ON, Canada

###### **Correspondance:** Suzanne M. Kelly

*Allergy, Asthma & Clinical Immunology* 2022, **17(Suppl 1)**: 46

**Background:** HAE is a genetic disease leading to intermittent attacks of angioedema of the face, the extremities, genitalia and the abdomen. Access to treatment may be impacted by the mode of delivery. Many approved medications are delivered intravenously. Newer approved medications are delivered subcutaneously. The oral medications used to treat HAE (androgen and tranexamic acid) are older, not approved for HAE (androgen), have unwanted side effects and are less effective. Newer, effective oral medications are desired by patients with HAE.

**Methods:** Using data from the HAE Canada patient surveys performed in 2017 and 2020, we analyzed and compared responses to questions on the type of medications used to treat HAE and number of attacks not treated because of access to medication.

**Results:** IV medication use was similar in 2017 (on-demand: 58.8%, prophylaxis 41.3%) and 2020 (on-demand: 41.3%, short-term (44.0%), long-term (32.5%) prophylaxis). Similarly, there was little change in oral medication use. In 2017: 3.8% for on-demand and prophylaxis; in 2020: 2.5%, on demand, 9.2% short-term and 3.4% long-term prophylaxis. By contrast, subcutaneous medication use has increased from in 2017: 10.0% on-demand, 1.25% prophylaxis to, in 2020, 60.4% on-demand, 28.1% short-term and 38.4% long-term prophylaxis. Percentages may exceed 100% because of multiple categories for use. In 2017, the number of attacks not treated because of medication access was zero for 4.35% of patients while in 2020 it was zero for 59.7%.

**Conclusions:** The use of newer, self-administered HAE treatments has significantly increased between 2017 and 2020. In parallel, the percent of patients with attacks not treated because of lack of access to medication has decreased significantly. Medications taken at home which are non-invasive and accessible (subcutaneous or oral) are desired by HAE patients and will likely have a significant impact on their quality of life.

## Other Allergy/Immunology

### 47 Allergic reactions to the COVID-19 vaccine: McGill University Health Center (MUHC) experience

#### Faisal ALMuhizi^1,2^, Michael Fein^1,5^, Sofianne Gabrielli^3^, Lene H. Garvey^4^, Christos Tsoukas^1,5^, Moshe Ben-Shoshan^3,5^, Ana Copaescu^1,5,6^, Ghislaine Isabwe^1,5^

##### ^1^Division of Allergy and Clinical Immunology, Department of Medicine, McGill University Health Centre (MUHC), McGill University, Montreal, QC, Canada, ^2^Department of Internal Medicine, Security Forces Hospital Program, Riyadh, Saudi Arabia, ^3^Division of Pediatric Allergy and Clinical Immunology, Department of Medicine, Montréal Children Hospital McGill University Health Centre, Montreal, QC, Canada, ^4^Department of Dermatology and Allergy, Allergy Clinic, Copenhagen University Hospital – Herlev and Gentofte, Copenhagen, Denmark, ^5^The Research Institute of the McGill University Health Centre, McGill University, Montreal, QC, Canada, ^6^Centre for Antibiotic Allergy and Research, Department of Infectious Diseases, Austin Health, Heidelberg, VIC, Australia

###### **Correspondance:** Faisal ALMuhizi

*Allergy, Asthma & Clinical Immunology* 2022, **17(Suppl 1)**: 47

**Background:** The coronavirus disease 2019 (COVID-19) pandemic presents an enormous challenge for public health and clinicians globally (1). Messenger ribonucleic acid (mRNA) vaccines contain polyethylene glycol (PEG) 2000, associated with allergic reactions at 2.5–11.1 cases/1 million doses (2). Our aim is to assess the validity of PEG skin prick testing (SPT) and the safety of COVID-19 vaccines in potentially high-risk candidates.

**Methods:** From January 1st to June 17th 2021, adult patients referred to the MUHC allergy clinics considered at risk of anaphylaxis were prospectively recruited. The entry criteria was any documented history of anaphylaxis. Evaluated patients underwent SPT with PEGs: PEG 35 (cremophor), PEG 3000 (50% w/v), PEG 3350 (50% w/v), Polysorbate 80 (20% w/v), PEG 20,000 (0.01%, 0.1%, 1%, and 10% w/v) (3). Following SPT, placebo-controlled vaccine challenges were carried out.

**Results:** Of the 70-patients that met the enrollment criteria; 16 (22.8%) had reacted to the first vaccine dose. Forty-two patients underwent SPT and 4 (9.5%) had a positive test. One had a positive challenge with oral PEG-3350, the others did not undergo oral challenge. Two tolerated the AstraZeneca vaccine. Of the remaining two patients, one had a delayed positive SPT to PEG and tolerated the Pfizer-BioNTech vaccine, while the other is pending vaccine challenge. Thirty-one patients (44.3%) underwent COVID vaccine challenge at the allergy clinic, 28 (40%) at the vaccination center, and 11 (15.7%) are pending challenge. Thirty-one (44.3%) tolerated the vaccine: 23 (74.2%) received single full-dose and 8 (25.8%) 2-split dose. Of the 28 who received the vaccine at a vaccination center, one reported a delayed large local erythema following both doses of the Pfizer-BioNTech vaccine.

**Conclusions:** In a cohort with a history of anaphylaxis and increased risk of allergic reactions to the COVID-19 vaccines, following allergist evaluation, including negative PEG skin testing, vaccine administration was safe without serious adverse events.

1. Copaescu A, Smibert O, Gibson A, Phillips EJ, Trubiano JA. The role of IL-6 and other mediators in the cytokine storm associated with SARS-CoV-2 infection. J Allergy Clin Immunol. 2020;146(3):518–34.e1.
2. Blumenthal KG, Robinson LB, Camargo CA, Jr., Shenoy ES, Banerji A, Landman AB, et al. Acute Allergic Reactions to mRNA COVID-19 Vaccines. Jama. 2021;325(15):1562–5.
3. Bruusgaard-Mouritsen MA, Jensen BM, Poulsen LK, Johansen JD, Garvey LH. Optimizing investigation of suspected allergy to polyethylene glycols. J Allergy Clin Immunol. 2021.

### 48 Use of skin testing in evaluation of patients with adverse events following immunization: A Canadian Immunization Research Network Study

#### Beth E. MacDonald^1^, Caroline Muñoz^2^, Manish Sadarangani^3^, Juthaporn Cowan^4^, Joseline Zafack^5^, Julia E. Upton^6^, Zainab Abdurrahman^7^, Mary McHenry^1^, Kyla Hildebrand^3^, Karina A. Top^1^

##### ^1^Canadian Center for Vaccinology, IWK Health Centre and Nova Scotia Health Authority, Dalhousie University, Halifax, NS, Canada, ^2^Cardio-Respiratory Research Lab, McMaster University, Hamilton, ON, Canada, ^3^Vaccine Evaluation Center, BC Children’s Hospital, University of British Columbia, Vancouver, BC, Canada, ^4^The Ottawa Hospital Research Institute, University of Ottawa, Ottawa, ON, Canada, ^5^Public Health Agency of Canada, Ottawa, ON, Canada, ^6^Division of Immunology and Allergy, Department of Paediatrics, Hospital for Sick Children, University of Toronto, Toronto, ON, Canada, ^7^Department of Pediatrics, McMaster Children’s Hospital, Hamilton, ON, Canada

###### **Correspondance:** Beth E. MacDonald

*Allergy, Asthma & Clinical Immunology* 2022, **17(Suppl 1)**: 48

**Background:** Vaccines may cause rare allergic reactions. The Special Immunization Clinic (SIC) Network evaluates patients with adverse events following immunization (AEFIs). Allergy skin testing is indicated for patients with suspected hypersensitivity reactions within 1 hour and anaphylaxis within 4 hours of vaccination. We sought to determine frequency of positive skin testing, revaccination recommendations, and AEFI recurrences among participants assessed in the SIC Network (2013–2019).

**Methods:** We analyzed SIC Network patients who required further doses of associated vaccine(s), underwent skin testing to a vaccine or excipient, and consented to participate. Revaccinated participants were followed up for AEFI recurrence. Data on referral indication, medical history, vaccinations, AEFI details, skin testing results and recurrences were collected. Skin testing results were compared between patients with a skin testing indication (referred for allergic-like event (ALE) onset < 4h or any AEFI onset < 1h). Data were extracted from the SIC Network database for descriptive analysis.

**Results:** Analysis included 147 participants; 98 (67%) had a skin testing indication. Among 49 participants tested outside indications, there were 18 ALE onset ≥ 4h, 6 injection site reactions, 5 non-urticarial rash, 2 vasovagal reactions, and 7 other AEFIs. Positive skin tests occurred in 25/98 (26%) participants with an indication versus 11/49 (22%) without. Among participants with positive testing, revaccination was recommended to 15/25 (60%) with an indication versus 7/11 (64%) without, and of those revaccinated, recurrences occurred in 5/13 (38%) versus 0/4 (p = 0.261). Among participants with negative testing, 94% were recommended for revaccination and of those revaccinated, recurrences occurred in 0/53 with a testing indication versus 3/22 (14%) without (p = 0.023). No recurrences were serious.

**Conclusions:** Participants underwent skin testing for a range of AEFIs and most were recommended for revaccination irrespective of results. Having an indication for skin testing was associated with decreased AEFI recurrence risk among participants with negative skin tests.

### 49 Safety of COVID-19 vaccines in patients on chronic immunoglobulin treatment for immunodeficiencies. Data from the Ontario Immunoglobulin Treatment (ONIT) registry

#### Juthaporn Cowan^1^, Stephen Betschel^2^, Susan Waserman^3^, D. William Cameron^1^

##### ^1^The Ottawa Hospital, Ottawa, ON, Canada, ^2^University of Toronto, Toronto, ON, Canada, ^3^McMaster University, Hamilton, ON, Canada

###### **Correspondance:** Juthaporn Cowan

*Allergy, Asthma & Clinical Immunology* 2022, **17(Suppl 1)**: 49

**Background:** Coronavirus disease-19 (COVID-19) vaccines were shown to be safe and effective in clinical trials of healthy individuals. Patients with immunodeficiencies or patients who were taking immunoglobulin treatment were excluded from the initial studies. Uncertainty about vaccine safety contributes to vaccine hesitancy in some immunodeficient patients. Nevertheless, mass vaccination and rapid vaccine rollout is required to halt the pandemic crisis. We aim to report the safety outcome of patients on chronic immunoglobulin treatment who received COVID-19 vaccines from January to September 2021.

**Methods:** Using the ONIT registry established at the Ottawa Hospital, Hamilton Health Sciences, and Unity Health Toronto, we will report on any medically attended adverse events following COVID-19 vaccination in patients who have been enrolled in the ONIT program. Immunization, infection history, immunoglobulin treatment indication, dosage and infusion parameters are core data that are being collected at every virtual or in-person clinic visit.

**Results:** To date (as of June 22, 2021), there are 239 patients registered in the ONIT registry at the Ottawa site. There were 201 clinic visits from Jan to Jun 2021. Of these, 52 received at least one dose of the COVID-19 vaccine. Median age 62 (range 21–84). Thirteen were fully vaccinated with homologous vaccine type. Of 65 doses given, 46 were Pfizer/BioNTech; 15 were Moderna; 4 were AstraZeneca vaccines. None had medically attended adverse events. No COVID-19 case was reported in vaccinated patients whereas there were two reported in the unvaccinated individuals. Both had underlying hematological malignancies. We will report updated data collected by September 2021 at the annual meeting.

**Conclusions:** COVID-19 vaccines appear to be safe in patients who are on chronic immunoglobulin treatment.

### 50 Covid Vaccine Evaluation of Resources and Solutions (COVERS)

#### Gregory Gooding^1^, Jennifer L. Protudjer^2,3,4^, Sofianne Gabrielli^1^, Greg Shand^1^, Christine McCusker^1^, Francisco Noya^1^, Maria Harvey^1^, Melodie Chalifour^1^, Catherine Sicard^5^, Elissa Abrams^2,3^, Jacques-Alexandre Amiel^5^, Moshe Ben-Shoshan^1^

##### ^1^Division of Allergy and Clinical Immunology, Department of Pediatrics, Montreal Children’s Hospital, McGill University Health Centre, Montreal, QC, Canada, ^2^Department of Pediatrics and Child Health, University of Manitoba, Winnipeg, MB, Canada, ^3^The Children’s Hospital Research Institute of Manitoba, Winnipeg, MB, Canada, ^4^George and Fay Yee Centre for Healthcare Innovation, Winnipeg, MB, Canada, ^5^Department of Pharmacy, Montreal Children’s Hospital, McGill University Health Center, Montreal, QC, Canada

###### **Correspondance:** Gregory Gooding

*Allergy, Asthma & Clinical Immunology* 2022, **17(Suppl 1)**: 50

**Background:** COVID-19 vaccination efforts focus on adolescents and adults and are expected to include children< age 12 years shortly. We aim to assess barriers and identify solutions for vaccination, especially in parents and their children.

**Methods:** Families seen at the outpatient allergy clinic at the Montreal Children’s Hospital and at a private allergy clinic were invited to complete an anonymous online survey on COVID-19 and vaccination. Statistical results were analyzed using R (version 4.0.0). Uni and multivariate logistic regressions were compared to estimate factors associated with vaccine hesitancy.

**Results:** Between May and June 2021, 97 parents and children responded to the survey. The majority (36.1%) of children were 6 to 10 years old (range: 0 – 17years). Most parents (56.7%) were 40–49 years (range: 20–59 years) and 41.2% had at least college level education. The most common barrier to vaccination was fear of adverse effects. (49.5%). Over half of families (56.7%) believed that a history of allergies was a contra indication for vaccination. Fifty-nine (60.8%) participants stated that dissemination of additional information would increase their willingness to be vaccinated. Educational videos (59.8%) were preferred by respondent. Health professionals i.e. (physicians, nurses and pharmacists) were the favored sources of information.

Most (96.9%) parents reported that their children’s vaccinations were up to date. Interestingly 76.3% of parents indicated they would vaccinate their children for while only 44.3% stated a plan to vaccinate for seasonal influenza in 2021 COVID (28.1% difference, 95%CI: 17.9%, 46.0%).

Hesitant parents were less likely to have children between the ages of 11–14 and were more likely to be of Asian descent while controlling for parental sex and education level.

**Conclusions:** Most families of children with food allergy plan to vaccinate their children for COVID-19. Videos addressing identified concerns and misinformation, especially the adverse effects of this vaccine would be reassuring.

### 51 Adverse reactions to mRNA COVID-19 vaccines at the Windsor Allergy Asthma Clinic

#### Akos A. Kazinczi

##### Windsor Allergy Asthma Clinic, Windsor, ON, Canada

###### **Correspondance:** Akos A. Kazinczi

*Allergy, Asthma & Clinical Immunology* 2022, **17(Suppl 1)**: 51

**Background:** Three COVID-19 vaccination clinics were opened during the spring of 2021 in association with the Windsor Allergy Asthma Clinic at the Moy Medical Center. These clinics included a general public clinic, a high-risk anaphylaxis clinic (concerns of potential anaphylaxis in 1st dose), and a desensitization clinic (patients with acute allergic reactions to 1st doses of COVID-19 mRNA vaccines).

**Methods:** A prospective observational trial from March 18 to June 23, 2021 was performed to determine the number of adverse/allergic reactions to the mRNA vaccines in each of these clinics. Pfizer-BioNTech or Moderna mRNA-1273 vaccines were provided in the general public clinic, while the Pfizer-BionTech vaccines were provided in the high-risk anaphylaxis clinic and desensitization clinic. The number of people experiencing adverse reactions within the first 15 minutes following immunization was recorded.

**Results:** A) Public Vaccination Clinic (N = 14401)—18 adverse reactions (0.125%) were recorded. 3 people (0.020%) experienced hypersensitivity type reactions ranging from transient dyspnea to systemic anaphylaxis, 1 person (0.007%) experienced a panic attack involving tachycardia, diaphoresis, and tachypnea, 11 people (0.076%) experienced vasovagal syncope, and 2 people (0.014%) experienced nausea with vomiting. 1 person (0.007%) experienced profound fatigue and was transported to urgent care.

B) High-risk Anaphylaxis Clinic (N = 131)—No adverse reaction was recorded among the patients that were vaccinated.

C) Desensitization Clinic (N = 11)—1 adverse reaction was recorded (9.090%). The patient was recorded to have a sustained elevation in blood pressure accompanying palpitations.

**Conclusions:** Adverse reactions to mRNA COVID-19 vaccines were found to be extremely rare. Only 1 person experienced systemic anaphylaxis. The partnership between public health and the Windsor Allergy Asthma Clinic was beneficial by providing reassurance with respect to anxiety of potential allergic reactions with the new mRNA vaccines.

### 52 Pandemic pandemonium and the power of placebo

#### Vince Wu, Ijaz Ogeer, Wardha Wardha, Jason A. Ohayon

##### Hamilton Allergy, Hamilton, ON, Canada

###### **Correspondance:** Vince Wu

*Allergy, Asthma & Clinical Immunology* 2022, **17(Suppl 1)**: 52

**Background:** With increasing uptake of COVID-19 vaccines and media focus on adverse reactions, questions and anxiety surrounding vaccinations intensified. The potential relationship between patient anxiety and adverse reactions risks vaccine hesitancy and the possibility of increased COVID-19 infections. The need for allergy consultation for adverse reactions to COVID-19 vaccines is essential in ensuring completion of vaccination protocols, especially in patients identified as having adverse reactions from first vaccinations.

**Methods:** Two patients with reactions to their first dose of the Pfizer-BioNTech COVID-19 vaccine were assessed for potential allergy. Symptoms included throat tightness, dyspnea, tingling, and dizziness immediately after vaccination. One of the patients required treatment with epinephrine with subsequent need for emergency room visit.

A two-step approach was taken to assess these patients for a potential allergic cause and exclude anxiety-induced reactions. Patients were blinded and initially skin tested to saline, observed for 15 minutes, and then assessed prior to skin testing to polyethylene glycol (PEG) as a suspected IgE mediated allergic trigger in the Pfizer-BioNTech vaccine.

**Results:** Both patients tested negative to saline. However, both experienced similar or worse symptoms as their initial reaction to Pfizer-BioNTech vaccination with dyspnea, pruritus, coughing, chest tightness, and dizziness during their allergy assessment. Notably, urticaria was absent.

One of the patients then went on to receive skin testing to PEG and tested negative.

Both patients later received their second vaccination without issue.

**Conclusions:** Two patients experienced “allergic-type” reactions after their first COVID-19 vaccine, not felt to be IgE mediated. Testing to saline elicited similar allergic-type symptoms, likely due to anxiety presenting as pseudo allergic reactions. Benefits of allergy consult and blinded testing to saline facilitated completion of vaccination.

### 53 Graded challenge with the SARS Co-V vaccine in patients with potential IgE mediated allergy to mRNA Vaccines: a case series

#### Jasper Johar, Gina Lacuesta, Pascale Clark, Lori Connors

##### Dalhousie University, Halifax, NS, Canada

###### **Correspondance:** Jasper Johar

*Allergy, Asthma & Clinical Immunology* 2022, **17(Suppl 1)**: 53

**Background:** There have been reported reactions to the SARS-CoV-2 mRNA vaccines in the literature ranging from mild dermatologic reactions to anaphylaxis. [1,2] To date, there exists little data as to whether patients can be challenged safely with the SARS-CoV-2 mRNA vaccine if they have had a prior severe allergic reaction.

**Methods:** We present a case series of patients at a single community based allergy clinic with a history consistent with possible IgE mediated reaction to a SARS-CoV-2 mRNA vaccine who received treatment with epinephrine. Epicutaneous prick testing was performed with either the Pfizer-BioNtech or Moderna SARS-CoV-2 mRNA vaccine, 1:10 dilution and then 1:1 dilution, with positive and negative controls. Patients with negative skin tests then went on to receive a two or three-step graded challenge with the tested vaccine. Steps were done in 30 minutes increments and each patient was observed for 1 hour after receiving the final dose. Patients were consented to a case series as part of our institution’s protocol.

**Results:** We did not identify any patients with positive skin prick testing to the SARS-CoV-2 mRNA vaccines, nor did any patients have objective findings of allergy on vaccine challenge.

**Conclusions:** In conclusion, graded challenge with the SARS-CoV-2 mRNA vaccine can be safely performed by an allergist in patients with a prior history of possible IgE mediated reaction to an mRNA vaccine.

**References:**

1. Allergic Reactions Including Anaphylaxis After Receipt of the First Dose of Pfizer-BioNTech COVID-19 Vaccine – United States, December 12–23, 2020. MMWR Morb Mortal Wkly Rep 2021;70:46–51.
2. Banerji A, Wickner PG, Saff R, Stone CA Jr, Robinson LB, Long AA, Wolfson AR, Williams P, Khan DA, Phillips E, Blumenthal KG. mRNA Vaccines to Prevent COVID-19 Disease and Reported Allergic Reactions: Current Evidence and Suggested Approach. J Allergy Clin Immunol Pract 2021. Apr;9(4):1423–1437.

### 54 Drug-induced anaphylaxis assessment: creating an online database of non-irritating drug concentrations for allergy skin testing

#### Christopher Knox^1^, Erika Lee^2^, Elizabeth Phillips^3^, Christine Song^2^, Peter Vadas^2^, Stephen Betschel^2^

##### ^1^Faculty of Medicine, University of Toronto, Toronto, ON, Canada, ^2^Division of Clinical Immunology and Allergy, St. Michael’s Hospital, Toronto, ON, Canada, ^3^Pharmacology & Pathology, Microbiology & Immunology, Vanderbilt University Medical Center, Nashville, TN, USA

###### **Correspondance:** Christopher Knox

*Allergy, Asthma & Clinical Immunology* 2022, **17(Suppl 1)**: 54

**Background:** Drug hypersensitivity reactions (DHRs) are common and represent 30–40% of all known causes of anaphylaxis.^1^ For drugs that have skin testing option available, DHRs can be assessed using skin prick test (SPT) and/or intradermal test (IDT) at non-irritating concentrations.^2^ Currently there is no central database with published non-irritating concentrations of drugs available for skin testing.

**Methods:** We began creating our database using the available non-irritating concentrations recommended in “Practical Guidance for the Evaluation and Management of Drug Hypersensitivity: Specific Drugs.”^3^ We then expanded the database by conducting a comprehensive literature search in Medline in order to collect the known non-irritating drug concentrations for skin testing (SPT/IDT), patch testing for delayed hypersensitivities, desensitization protocols, and cross-reactivity amongst drugs. When selecting articles, we gave preference for recent articles, those that were relevant to our search, or from a top Immunology journal.

**Results:** Our database contains references to over 500 articles and spans over 400 drugs, ranging from a wide variety of categories including antimicrobials, chemotherapeutics, biologicals, peri-operative agents, anticoagulants and coagulation factors. For each drug, the user is provided with SPT/IDT concentrations, patch testing concentrations, references to desensitization protocols that have been used, and allergic cross-reactivity amongst drugs. We created a prototype website to allow for users to easily interact with the database.

**Conclusions:** The creation of this database with a collection of known drug concentrations for skin testing, patch testing, desensitization protocols and allergic cross-reactivity amongst drugs will allow physicians to access the information readily, effectively and efficiently in a busy clinic. Future directions include creating an online version of the database and a monitoring system where allergists can submit newly published non-irritating concentrations to the website.

**References:**

1. Wood RA, Camargo CA, Lieberman P, Sampson HA, Schwartz LB, Zitt M, et al. Anaphylaxis in America: the prevalence and characteristics of anaphylaxis in the United States. J Allergy Clin Immunol. 2014;133(2):461–7.
2. Montañez MI, Mayorga C, Bogas G, et al. Epidemiology, Mechanisms, and Diagnosis of Drug-Induced Anaphylaxis. Front Immunol. 2017;8:614. Published 2017 May 29. doi:10.3389/fimmu.2017.00614
3. Broyles AD, Banerji A, Barmettler S, et al. Practical Guidance for the Evaluation and Management of Drug Hypersensitivity: Specific Drugs [published correction appears in J Allergy Clin Immunol Pract. 2021 Jan;9(1):603] [published correction appears in J Allergy Clin Immunol Pract. 2021 Jan;9(1):605]. J Allergy Clin Immunol Pract. 2020;8(9S):S16-S116. doi:10.1016/j.jaip.2020.08.006

### 55 Cannabis Allergy: a 4 patient case series

#### Jasper Johar, Lori Connors

##### Dalhousie University, Halifax, NS, Canada

###### **Correspondance:** Jasper Johar

*Allergy, Asthma & Clinical Immunology* 2022, **17(Suppl 1)**: 55

**Background:** With the legalization of cannabis occurring in Canada and worldwide, the incidence of cannabis allergy is becoming increasingly recognized. [1] Reactions ranging from rhinitis to anaphylaxis with cannabis exposure have been described, highlighting the need to identify cannabis allergy. [2] Allergy to cannabis is felt to be due to Can s 3, a nonspecific lipid transfer protein, with several other cannabis allergens likely playing a role. [3] A prior study has demonstrated that Can s 3 is a reliable extract for skin prick testing and can identify positive individuals who are sensitized for cannabis allergy. [3] This extract is not yet readily available in most centers.

**Methods:** Here, we present a 4 patient case series of patients with respiratory symptoms with inhalational exposure to cannabis, as well as and one with contact urticaria with cannabis exposure. Patients were consented to a case series as part of our institution’s protocol.

**Results:** All four patients had positive skin prick testing for tree pollen, as well as their individual extracts of cannabis flower which they reacted to. One patient who had reacted to cannabis oil and flower had positive skin prick testing to only flower and not oil. Three among the five are continuing to use cannabis at risk, and have not yet presented with anaphylaxis.

**Conclusions:** The use of cannabis flower for skin testing is a useful alternative to commercial extract testing. As cannabis use increases, it is prudent for allergists to include cannabis use and potential reactivity as a routine part of our history taking. Future research is needed in the utility and practicalities of cannabis challenges in the allergist’s office.

**References:**

1. Jackson, B., Cleto, E. and Jeimy, S. An emerging allergen: Cannabis sativa allergy in a climate of recent legalization. Allergy Asthma Clin Immunol 2020. 16:53.
2. Liskow B, Liss JL, Parker CW. Allergy to marihuana. Ann Intern Med 1971. **75**(4): 571–3.
3. Decuper II., Van Gasse AL., Faber AF, Elst J., Mertens C., Rihs HP et al. Exploring the Diagnosis and Profile of Cannabis Allergy. Journal of Allergy and Clin Immunol 2019. 7,3: 983–989.

### 56 The development and implementation of a proactive penicillin allergy de-labelling program for low risk inpatients at The Ottawa Hospital

#### Derek Lanoue^1,4^, Derek MacFadden^1,2,4^, Carl Vanwalraven^1,2,4^, Tim Olynych^1,3,4^, Caroline Nott^1,2,4^

##### ^1^The Ottawa Hospital, Ottawa, ON, Canada, ^2^The Ottawa Hospital Research Institute, Ottawa, ON, Canada, ^3^Yang Medicine, Ottawa, ON, Canada, ^4^University of Ottawa, Ottawa, ON, Canada

###### **Correspondance:** Derek Lanoue

*Allergy, Asthma & Clinical Immunology* 2022, **17(Suppl 1)**: 56

**Background:** Penicillin allergy is estimated to affect approximately 10% of hospitalized patients and is associated with numerous adverse outcomes.^1^ Several allergy assessment modalities are available contingent upon the clinical history. Direct oral challenge has emerged as a safe, effective way of evaluating patients with low risk of penicillin allergy by history.^2^ There is increasing support that proactively assessing penicillin allergy in hospitalized patients should be incorporated into antimicrobial stewardship programs.^3^

**Methods:** We piloted a penicillin allergy de-labelling program where all inpatients were proactively identified, screened, and if deemed low-risk by history (reactions greater than 10 years ago that were not severe cutaneous adverse reaction or anaphylaxis) administered a single 250mg amoxicillin oral challenge without preceding skin testing and monitored for 1 hour. The goal of the pilot program was to establish feasibility, resource utilization, and challenge outcomes.

**Results:** Between April 16th and April 30th 2021 there were 66 patients admitted to the Ottawa Civic Hospital with penicillin allergy label on their Electronic Medical Record (EMR). 38 were excluded because of clinical instability, cognitive impairment or pregnancy. 9 patients were deemed to have a moderate or high-risk penicillin allergy. 19 patients were deemed low-risk and appropriate for a penicillin oral challenge. 14 consented and were challenged in hospital. All 14 patients tolerated the challenge and were de-labelled on their hospital EMR with a note sent to their family physician and home pharmacy. There were no adverse events during oral challenges.

**Conclusions:** Our pilot study demonstrates that a proactive approach including direct oral challenge in low risk inpatients with penicillin allergy is a safe and feasible way to de-label patients. Our approach could be implemented more broadly as part of antimicrobial stewardship efforts across Canada.

**References:**

1. Sousa-Pinto, B., Cardoso-Fernandes, A., Araújo, L., Fonseca, J. A., Freitas, A., & Delgado, L. (2018). Clinical and economic burden of hospitalizations with registration of penicillin allergy. *Annals of Allergy, Asthma and Immunology*, *120*(2), 190–194.e2.
2. Trubiano, J. A., Thursky, K. A., Stewardson, A. J., Urbancic, K., Worth, L. J., Jackson, C., Phillips, E. J. (2017). Impact of an Integrated Antibiotic Allergy Testing Program on Antimicrobial Stewardship: A Multicenter Evaluation. *Clinical Infectious Diseases*, *65*(1), 166–174.
3. Macy E, Khan DA, Castells MC, Lang DM. Penicillin Allergy Testing: A Key Component of Antibiotic Stewardship. Clin Infect Dis. 2017 Feb 15;64(4):531–532.

### 57 Assessment of multiple opinion consults to the BC Children’s Hospital Allergy Clinic

#### Elliot James^1^, Edmond S. Chan^1^, Lianne Soller^1,2^, Tiffany Wong^1^

##### ^1^Division of Allergy and Clinical Immunology, Department of Pediatrics, BC Children’s Hospital, The University of British Columbia, Vancouver, BC, Canada, ^2^BC Children’s Hospital Research Institute, Vancouver, BC, Canada

###### **Correspondance:** Elliot James

*Allergy, Asthma & Clinical Immunology* 2022, **17(Suppl 1)**: 57

**Background:** Multiple opinion referrals contribute to long clinic waitlists, resulting in delayed treatment of patients with allergic disease and increased healthcare costs. Since the prevalence of allergic disease is increasing, it is important to determine and minimize the motivating factors behind multiple opinion referrals to ensure timely access and delivery of medical care. The impact of multiple opinion referrals in allergy has not been documented. This study aimed to determine how often multiple opinion referrals are occurring in BC Children’s Hospital (BCCH) Allergy Clinic, and why.

**Methods:** A retrospective chart review of new consults to BCCH Allergy Clinic from September 1, 2016–August 31, 2017 was performed. A data abstraction form was used to collect information. Preliminary data analysis included reporting frequencies and proportions for categorical variables. A one-sample t-test for proportions compared patients seeking multiple opinions for food allergy versus other allergic conditions.

**Results:** 218 out of 1037 (21%) of new consults assessed in our clinic have previously received allergy services from another healthcare provider, including other allergists (52.3%), general pediatricians (21.9%), and naturopaths (15%). 80.8% of patients previously received validated or scientifically unproven allergy tests including skin prick, serum sIgE, serum sIgG, Vega, and applied kinesiology testing. Patients were more likely to seek multiple opinions for food allergy concerns (74.3%) than all other allergic conditions combined (25.7%) (p < 0.001). Most common reasons for seeking another opinion included: seeking formal allergist assessment after receiving non-allergist advice (32.1%), and dissatisfaction with previous opinion (16.5%).

**Conclusions:** This is the first study evaluating multiple opinion consults in an allergy clinic setting. We found that such referrals are relatively common, particularly for food allergy concerns. Multiple opinion referrals may be reduced by referring patients directly to allergists and improving satisfaction with initial consultation visits. Further quality improvement work is required to reduce multiple opinion consults to allergists.

### 58 Economic evaluation of budesonide orodispersible tablets for the treatment of eosinophilic esophagitis: a cost-utility analysis

#### Catherine Beauchemin^2,8^, Alex Castonguay^2^, Edmond S. Chan^3^, Evan S. Dellon^4,7^, Brian G. Feagan^5,6,9^, Christopher Ma^9,10^, Susan Waserman^11^, John Cook^12^, David Claveau^1^

##### ^1^AVIR Pharma Inc., Blainville, QC, Canada, ^2^PeriPharm Inc., Montreal, QC, Canada, ^3^Division of Allergy & Immunology, Department of Pediatrics, University of British Columbia, British Columbia Children’s Hospital, Vancouver, BC, Canada, ^4^Center for Esophageal Diseases and Swallowing, Division of Gastroenterology and Hepatology, University of North Carolina School of Medicine, Chapel Hill, NC, USA, ^5^Department of Medicine, Western University, London, ON, Canada, ^6^London Health Sciences Centre, London, ON, Canada, ^7^Center for Gastrointestinal Biology and Disease, Division of Gastroenterology and Hepatology, University of North Carolina School of Medicine, Chapel Hill, NC, USA, ^8^University of Montreal, Montreal, QC, Canada, ^9^Alimentiv Inc., London, ON, Canada, ^10^Division of Gastroenterology and Hepatology, Departments of Medicine & Community Health Sciences, University of Calgary, Calgary, AB, Canada, ^11^Division of Allergy and Clinical Immunology, Department of Medicine, McMaster University, Hamilton, ON, Canada, ^12^CHEORS, North Wales, PA, USA

###### **Correspondance:** David Claveau

*Allergy, Asthma & Clinical Immunology* 2022, **17(Suppl 1)**: 58

**Background:** Budesonide orodispersible tablets (BOT) have been approved in Europe and Canada for the treatment of eosinophilic esophagitis (EoE), a rare and chronic disease. The objective of this study was to assess the economic impact of BOT on both the induction and maintenance of clinico-pathological remission of EoE by performing a cost-utility analysis (CUA).

**Methods:** For both the induction and maintenance settings, BOT was compared to no treatment in a target population of adult patients with EoE non-responsive to proton pump inhibitor (PPI) treatment. Markov models were developed for the induction and maintenance setting over a 52-week and life-time horizon, respectively. Analyses were performed from both a Canadian Ministry of Health (MoH) and societal perspective. The resulting incremental cost-utility ratios (ICURs) were compared to a willingness-to-pay (WTP) threshold of $50,000 Canadian dollars/quality-adjusted life-year (QALY). Sensitivity and scenario analyses were conducted to assess the robustness of the base-case results.

**Results:** In the base-case probabilistic analysis, BOT compared to no treatment resulted in an ICUR of $1,073/QALY and $30,555/QALY from a MoH perspective in the induction and maintenance setting, respectively. BOT was a cost-effective option for both induction and maintenance in > 99% of Monte Carlo simulations. In the scenario analyses, the deterministic ICUR of BOT compared to no treatment varied from $682/QALY to $8,510/QALY in the induction setting and $21,005/QALY to $55,157/QALY in the maintenance setting.

**Conclusions:** BOT was cost-effective compared to no treatment for both the induction and maintenance of clinico-pathological remission of EoE in patients non-responsive to PPIs.

### 59 Cannabis allergy: a retrospective review of a Vancouver allergy practice

#### Shun Chi Ryan Lo, Hasandeep Kular, Amin Kanani

##### University of British Columbia, Vancouver, BC, Canada

###### **Correspondance:** Shun Chi Ryan Lo

*Allergy, Asthma & Clinical Immunology* 2022, **17(Suppl 1)**: 59

**Background:** *Cannabis sativa* and *Cannabis indica* are plant species from which hemp products are derived. Preparations of cannabis with dried leaves or flowers, or marijuana, are also used recreationally or medicinally for the psychoactive effects of its cannabinoids. Cannabis allergy is increasingly reported in literature. Symptoms range in severity from mild to life-threatening, typically depending on route of consumption. Cross-reaction with fruits and vegetables has also been described, through sensitization to non-specific lipid transfer proteins. With the gradual increase in cannabis consumption in Canada since the Cannabis Act in 2018, cannabis allergy is accordingly expected to be more prevalent. Here, we aim to better characterize cannabis allergy in the British Columbia population via retrospective review.

**Methods:** Medical records of an allergy practice dating from 1990 to 2018 were screened for reports of “cannabis,” “marijuana,” or “hemp” allergy. Patient characteristics, attributed symptoms, atopic comorbidities and results of crude marijuana extract or hemp skin testing, where available, were compiled.

**Results:** Among 17 identified patients, 11 were male (64%) and average age was 38.6 years. 6 patients (35%) reported rhinitis, 6 patients (35%) reported worsening asthma, 7 patients (41%) reported urticaria, and 3 (18%) patients reported anaphylaxis. Symptoms developed with smoking marijuana in 6 patients (35%), with touching marijuana in 9 patients (53%) and with ingesting hemp in 3 patients (18%). Skin testing was positive in 6 out of 7 (86%) patients tested to marijuana, and in 3 out of 4 (75%) patients tested to hemp. Atopic comorbidities included allergic rhinitis (76%), asthma (53%), food allergy (18%) and oral allergy syndrome (41%).

**Conclusions:** Our study characterized patients with a variety of symptoms suggestive of cannabis allergy. Atopic comorbidities were common. The results highlight the importance of future studies to better understand the impact of cannabis allergy, especially when validated skin testing becomes available.

### 60 Canadian practice pattern on diagnosis and management of eosinophilic esophagitis

#### Emran Bashar, Marcela Roquim, Jodi Faulkner, David Claveau

##### AVIR Pharma Inc, Blainville, QC, Canada

###### **Correspondance:** Emran Bashar

*Allergy, Asthma & Clinical Immunology* 2022, **17(Suppl 1)**: 60

**Background:** The real-world Canadian practice pattern (PP) of diagnosis and management of eosinophilic esophagitis (EoE) is not well studied. The aim of this study is to report on any differences in practice and characterize adherence to guidelines.

**Methods:** A cross-sectional online survey containing 15 questions was conducted among 43 gastroenterologists and allergists caring for pediatric and adult EoE patients from academic and community settings from multiple Canadian cities.

**Results:** Of 43 respondents, around 90% were adult practitioners with 84% GIs and 14% allergists. 10% of participants preferred a proton pump inhibitors (PPIs) trial before diagnosing EoE, which is consistent with the proportion of responders who were not familiar with the new 2020 AGA JTF guidelines. Most participants performed biopsies in mid and distal (approx. 85%) over proximal segments (63%). More than 50% of participants used PPIs as first line therapy whereas 31% of respondents considered patient characteristics before choosing a therapy. When it came to steroid treatment, 50% preferred budesonide slurry, followed by fluticasone inhaler (34%). The use of scoring systems (EoEHSS, EEsAII-Pro, EREFS) while following patients was not common. Clinical and symptom remission had higher preference over endoscopic remission in terms of treatment success. In contrast to other recent international PP survey results, a higher proportion (75%) of practitioners prescribed maintenance therapy for more than 50% of patients. For patients using maintenance therapy, a majority of specialists only performed scoping at 2 years or when patients experience symptoms.

**Conclusions:** There is a substantial variability among specialists in terms of diagnosis and management of EoE in Canada. While most of them were familiar with recent international guidelines, some standardization is still required on a recommended approach to tailor treatment choices, define treatment success and follow patients in order to improve outcomes and prevent recurrences and disease progression.

### 61 Application of a digital point-of-care tool to assess patients with suspected beta-lactam allergies: a quality improvement initiative

#### Patrick R. Mckernan^1^, Scott Cameron^3,2,1^, Raymond Mak^3,2^, Tiffany Wong^3,2^

##### ^1^Department of Pediatrics, Faculty of Medicine, University of British Columbia, Victoria, British Columbia, Canada, Victoria, BC, Canada, ^2^BC Children’s Hospital Research Institute, Vancouver, BC, Canada, ^3^Division of Allergy & Immunology, Department of Pediatrics, Faculty of Medicine, University of British Columbia, Vancouver, BC, Canada

###### **Correspondance:** Patrick R. Mckernan

*Allergy, Asthma & Clinical Immunology* 2022, **17(Suppl 1)**: 61

**Background:** Beta-lactam allergies are commonly misdiagnosed in the general population leading to negative outcomes including increased use of alternative antibiotics, rates of antibiotic resistance, duration of hospitalizations, and medical costs [1]. There is a paucity of comprehensive and user-friendly clinical resources to help healthcare providers efficiently identify and de-label low-risk individuals.

**Methods:** We led a multidisciplinary team in British Columbia to develop a mobile point-of-care tool. The tool’s algorithm is adapted from Canadian practice guidelines [1,2]. The tool was piloted locally in May 2021 and launched in June 2021. Our aims are to promote public education and reduce unnecessary referrals to allergists. By Oct 2021, we aim to have 300 user hits, achieve de-labeling for at least 50% of clinical encounters, and have the tool adopted by 6 health authorities by Oct 2021.

**Results:** Our tool has been adopted at the BC Women’s and Children’s Hospital and Vancouver Island Health Authority.

As of June 24, 2021, preliminary data shows:

- 53 total user hits
- 21 patients assessed to be low risk
- 18 patients assessed not to be allergic based on history alone
- 5 patients that tolerated oral challenges
- 5 patients assessed to have a possible allergy
- 44% of outcomes occurred in a clinical setting

**Conclusions:** This mobile app is a unique and practical evidence-based tool to help assess and manage patients labeled with beta-lactam allergy. It represents an important step in removing barriers to the identification and removal of erroneous beta-lactam allergies. More work is required to achieve widespread awareness and usage of this tool in various practice settings.

**References:**

1. Jeimy, S., Ben-Shoshan, M., Abrams, E.M. et al: Practical guide for evaluation and management of beta-lactam allergy: position statement from the Canadian Society of Allergy and Clinical Immunology. Allergy Asthma Clin Immunol 16, 95 (2020). https://doi.org/10.1186/s13223-020-00494-2
2. Tiffany Wong, Adelle Atkinson, Geert t’Jong, Michael J Rieder, Edmond S Chan, Elissa M Abrams, Beta-lactam allergy in the pediatric population, Paediatrics & Child Health, Volume 25, Issue 1, February 2020, Page 62, https://doi.org/10.1093/pch/pxz179

### 62 Epicutaneous testing for Polyethylene Glycol in Patients with a suspected IgE mediated reaction to PEG asparaginase: a case series

#### Jasper Johar, Mary McHenry, Gina Lacuesta, Pascale Clark, Lori Connors

##### Dalhousie University, Halifax, NS, Canada

###### **Correspondance:** Jasper Johar

*Allergy, Asthma & Clinical Immunology* 2022, **17(Suppl 1)**: 62

**Background:** There have been reported reactions to the Pfizer-BioNtech and Moderna SARS Co-V vaccines in the literature ranging from localized skin reactions to anaphylaxis. [1,2] Polyethylene glycol (PEG), used as a nanoparticle stabilizer of mRNA in the above two vaccines, has been identified as among the most likely allergenic compounds in the vaccine. [3] PEG asparaginase is a common chemotherapeutic agent used in treatment of childhood leukemias, which is a form of *Escherichia coli L-asparaginas*e covalently linked to polyethylene glycol. There exists little evidence in using epicutaneous testing for PEG in patients with a label of allergy to asparaginase.

**Methods:** We present a case series of patients at a single community centre with history of possible IgE mediated reactions to PEG-asparaginase. Patients underwent epicutaneous testing to PEG with 1:10 and 1:1 dilutions. Patients were consented to a case series as part of our institution’s protocol.

**Results:** None of the patients with a label of PEG asparaginase allergy had positive skin prick testing to PEG. Further follow-up is planned to determine SARS Co-V vaccine tolerability.

**Conclusions:** Further data is required to determine whether negative PEG epicutaneous testing is useful in patients with a history of PEG-asparaginase allergy, in the context of safety of SARS Co-V vaccines.

**References:**

1. Allergic Reactions Including Anaphylaxis After Receipt of the First Dose of Pfizer-BioNTech COVID-19 Vaccine – United States, December 12–23, 2020. MMWR Morb Mortal Wkly Rep 2021;70:46–51.
2. Allergic Reactions Including Anaphylaxis After Receipt of the First Dose of Moderna COVID-19 Vaccine – United States, December 21, 2020 – January 10, 2021. MMWR Morb Mortal Wkly Rep 2021;70:125–129.
3. Banerji A, Wickner PG, Saff R, Stone CA Jr, Robinson LB, Long AA, Wolfson AR, Williams P, Khan DA, Phillips E, Blumenthal KG. mRNA Vaccines to Prevent COVID-19 Disease and Reported Allergic Reactions: Current Evidence and Suggested Approach. J Allergy Clin Immunol Pract. 2021 Apr;9(4):1423–1437.

## Urticaria/Angioedema

### 63 Acute and chronic spontaneous urticaria patients report increased rates of medication allergies.

#### Brandon R. Clark^1^, Judy Bornais^2^, Scott Miller^3^, Joel Liem^1,4^

##### ^1^Schulich School of Medicine & Dentistry, London, ON, Canada, ^2^Faculty of Nursing, University of Windsor, Windsor, ON, Canada, ^3^Windsor-Essex County Health Unit, Windsor, ON, Canada, ^4^Windsor Asthma & Allergy Clinic, Windsor, ON, Canada

###### **Correspondance:** Brandon R. Clark

*Allergy, Asthma & Clinical Immunology* 2022, **17(Suppl 1)**: 63

**Background:** The pathophysiology of acute urticaria and chronic spontaneous urticaria (CSU) is not completely known. Antibiotic allergies are on the rise, and the typical history is associated with urticarial reactions which can persist. Given this overlap, we sought to determine whether self-reported antibiotic allergies are increased in patients with a history of acute urticaria or CSU.

**Methods:** Retrospective chart review of 950 patients was completed using the electronic medical records of the Windsor Allergy & Asthma Clinic. Cases were identified as having a history of acute urticaria or CSU, whereas controls had an established diagnosis of food allergies, asthma, allergic rhinitis or non-allergic rhinitis. The number of reported medication and antibiotic allergies were compared between the case and control groups using Pearson’s chi square analyses.

**Results:** Patients with a history of urticaria were more likely to report an antibiotic allergy than would be expected by chance when compared to our control group (p = 0.023). A total of 42/239 (17.6%) patients with a history of urticaria (acute or chronic) vs 82/711 (12%) of the control group reported one antibiotic allergy. Additionally, 8/239 (3.3%) of our cases (acute or chronic) versus 14/711 (2%) of our control group reported 2 antibiotic allergies. Subgroup analyses looking at whether the nature of urticaria, acute vs. chronic, affected the allergy reporting risk did not find a significant difference. Both groups were more likely to report antibiotic allergies when compared to the control group (p = 0.036).

**Conclusions:** These results raise concern regarding the nature of self-reported antibiotic allergies in patients with a history of urticaria. These “antibiotic allergies” may be a result of concomitant urticarial exacerbations rather than true IgE mediated mechanisms. This has implications on choice of antibiotic agents in the era of antibiotic stewardship and growing microbial resistance.

### 64 Prevalence, management and anaphylaxis risk of cold urticaria: a systematic review and meta-analysis

#### Connor Prosty^1^, Sofianne Gabrielli^2^, Michelle Le^3^, Luis F. Ensina^4^, Xun Zhang^5^, Elena Netchiporouk^3^, Moshe Ben-Shoshan^2^

##### ^1^Faculty of Medicine, McGill University, Montreal, QC, Canada, ^2^Division of Allergy, Immunology and Dermatology, McGill University, Montreal, QC, Canada, ^3^Division of Dermatology, McGill University, Montreal, QC, Canada, ^4^Department of Pediatrics, Federal University of São Paolo, São Paolo, SP, Brazil, ^5^Centre for Outcome Research and Evaluation, Research Institute of McGill University Health Centre, Montreal, QC, Canada

###### **Correspondance:** Connor Prosty

*Allergy, Asthma & Clinical Immunology* 2022, **17(Suppl 1)**: 64

**Background:** Cold urticaria is a subtype of chronic inducible urticaria (CIndU) associated with significant morbidity and a risk of life-threatening allergic reactions. To date, few studies have assessed the prevalence, management, and resolution rate of cold urticaria and the prevalence of associated anaphylaxis.

**Methods:** A search of English and French-language observational studies reporting on cold urticaria and/or CIndU published in the last 10 years (2011 to 2021) was conducted using PubMed and EMBASE databases. Studies with at least 10 patients with cold urticaria were included in the systematic review and studies with at least 30 patients were included in the meta-analysis. Five meta-analyses were conducted to evaluate: the prevalence of cold urticaria among CIndU and chronic urticaria (CU) cases, the management of cold urticaria with H1-antihistamines and omalizumab and the prevalence of associated anaphylaxis.

**Results:** Twenty-two studies were included in the systematic review and 14 in the meta-analysis. The pooled prevalence of cold urticaria among CU and CIndU cases were 7.62% (95% CI: 3.45%, 15.99%; I^2^ = 98%) and 26.10% (95% CI: 14.17%, 43.05%; I^2^ = 97%), respectively. Cold urticaria was managed by H1-antihistamines in 95.67% (95% CI: 92.47%, 97.54%; I^2^ = 38%) of patients and omalizumab in 5.95% (95% CI: 2.55%, 13.27%; I^2^ = 83%) of patients. The five and 10-year resolution rates ranged from 13.8–26.6% and 24.5–44.5%, respectively. The pooled prevalence estimate of anaphylaxis among cold urticaria cases was 21.49% (95% CI: 15.79%, 28.54%; I^2^ = 69%). No fatalities were reported in any of the studies.

**Conclusions:** Our results indicate that cold urticaria constitutes an appreciable proportion of CIndU and CU cases. The vast majority of patients are treated with H1-antihistamines and a minority with omalizumab. However, anaphylaxis is not rare, and the prescription of an epinephrine auto-injector prescription should be considered.

### 65 Correlation of UAS7 and CDLQI score among children with Chronic Spontaneous Urticaria

#### Sofianne Gabrielli^1^, Gregory Gooding^1^, Michelle Le^2^, Lydia Zhang^1^, Elena Netchiporouk^2^, Sharon Baum^3^, Shoshana Greenberger^4^, Luis F. Ensina^5^, Fatemeh Jafarian^2^, Xun Zhang^6^, Moshe Ben-Shoshan^1^

##### ^1^Division of Pediatric Allergy and Clinical Immunology, Department of Pediatrics, McGill University Health Centre, Montreal, QC, Canada, ^2^Division of Dermatology, McGill University, Montreal, QC, Canada, ^3^Department of Dermatology, Chaim Sheba Medical Center, Tel-Aviv University, Sackler School of Medicine, Tel Hashomer, Israel, ^4^Department of Dermatology, Sheba Medical Center, Ramat-Gan, Israel, ^5^Department of Pediatrics, Federal University of Sao Paolo, Sao Paolo, SP, Brazil, ^6^Centre for Outcome Research and Evaluation, Research Institute of McGill University Health Centre, Montreal, QC, Canada

###### **Correspondance:** Sofianne Gabrielli

*Allergy, Asthma & Clinical Immunology* 2022, **17(Suppl 1)**: 65

**Background:** Chronic spontaneous urticaria (CSU) is defined as the presence of wheals and/or angioedema for more than six weeks. Changes in weekly Urticaria Activity Score (UAS7), calculated as the sum of scores of the number of hives and pruritus severity over 7 days, strongly correlate with changes to the Dermatology Life Quality Index (DLQI) score in adults. However, the UAS7 has not yet been validated for children with CSU. We aimed to assess if UAS7 was correlated with the Children’s DLQI (CDLQI) score among children with CSU.

**Methods:** Children with CSU were recruited at the Montreal Children’s Hospital from 2019 to 2020. Data on demographics, clinical characteristics, UAS7, and CDLQI score was collected at study entry through a standardized questionnaire. Pearson’s correlation coefficient was calculated to assess the correlation between UAS7 and CDLQI scores in children.

**Results:** Forty-six children were included in the analysis, of which 24 (52.2%) were male. The median age of appearance of urticaria was 8.0 years [Interquartile Range (IQR: 3.4, 12.4]. At study entry, the mean UAS7 was 14.5 [Standard deviation (SD): 14.2)] on a scale of 42 and the mean CDLQI score was 5.7 (SD: 4.9) on a scale of 30. UAS7 and CDLQI score were found to be positively correlated, r = 0.41, p-value = 0.004.

The majority of patients were treated with antihistamines (89.1%) with 78% taking the standard dose, 17.1% taking double and 4.9% taking four-times the standard dose. Seven patients (15.2%) required treatment with omalizumab. Treatment with omalizumab was associated with elevated UAS7 score at baseline [adjusted Odds Ratio (aOR) 1.02 (95%CI 1.02, 1.03)] while adjusting for age and sex.

**Conclusions:** Our findings suggest that the UAS7 score can correlate with CSU activity on children’s quality of life. However, larger-scale studies are needed to evaluate the validity of UAS7 on urticaria severity in children.

### 66 Clinical characteristics, diagnosis and management of hereditary angioedema (HAE): A Canadian single center retrospective review

#### Angeliki Barlas, Hasandeep Kular, Amin Kanani

##### Clinical Immunology and Allergy, University of British Columbia, Vancouver, BC, Canada

###### **Correspondance:** Angeliki Barlas

*Allergy, Asthma & Clinical Immunology* 2022, **17(Suppl 1)**: 66

**Background:** Hereditary angioedema (HAE) is a result of C1 inhibitor deficiency that leads to painful swelling of the extremities, bowel mucosa, face and upper airway [1]. It is a rare condition that can lead to life-threatening swelling attacks [1]. Over time, there have been updates to the management of HAE, including the use of icatibant and self-administration of C1-esterase inhibitor [2]. This retrospective review creates a better understanding of the disease presentation, demographics, treatment outcomes and health care utilization in individuals with HAE.

**Methods:** The clinical data of 44 HAE patients were reviewed and analyzed from a private electronic medical record retrospectively from January 1, 1990 to March 15, 2019.

**Results:** A total of 44 patient charts were evaluated. The average age at diagnosis was 22 with an average age of 40 at the end of the study. A mean of 88.6% of patients reported a family history of HAE. The mean number of attacks among study patients was 14, with the majority of attacks lasting between 24 to 72 hours. The most common sites of swelling reported by patients were peripheral, abdominal and facial. A mean of 40.9% of patients were treated prophylactically with danazol, 38.6% with C1-esterase inhibitor, and 15.9% with tranexamic acid at some point during their diagnosis. Among all patients, the mean number of emergency room visits for swelling attacks was four for the duration of the study.

**Conclusions:** Timely recognition and optimal treatment are important for the management of HAE and creating a database to identify clinical outcomes and health care utilization is imperative. This study is the first retrospective review of HAE patients in British Columbia and will add to the literature in recognizing and managing HAE as well as describing the standard of care for these patients.

**References:**

1. Betschel S et al. Canadian hereditary angioedema guideline. Allergy, asthma, and clinical immunology. 2014;10:50.
2. Cicardi M, Craig TJ, Martinez-Saguer I, Hébert J, Longhurst HJ. Review of Recent Guidelines and Consensus Statements on Hereditary Angioedema Therapy with Focus on Self-Administration. International Archives of Allergy and Immunology. 2013;161:3–9.

### 67 Long-term efficacy of enoxaparin for refractory chronic spontaneous urticaria with elevated d-dimer: a case series

#### Stephanie G. Brooks^1,2^, Mina Abbaslou^2^, Alexander Shusterman^2^, Anna M. De Jong^2,3^, Gordon Sussman^2,4^

##### ^1^Temerty Faculty of Medicine, University of Toronto, Toronto, ON, Canada, ^2^Gordon Sussman Clinical Research Inc., North York, ON, Canada, ^3^Faculty of Science, University of Western Ontario, London, ON, Canada, ^4^Department of Medicine and Division of Clinical Immunology & Allergy, University of Toronto, Toronto, ON, Canada

###### **Correspondance:** Stephanie G. Brooks

*Allergy, Asthma & Clinical Immunology* 2022, **17(Suppl 1)**: 67

**Background:** Refractory chronic spontaneous urticaria (CSU) negatively impacts quality of life. There are reports of successfully treated CSU with low molecular weight heparin [1,2] but without long-term follow up. In this study we investigate the long-term efficacy of enoxaparin for severe refractory CSU.

**Methods:** A retrospective chart review was performed for patients seen at an Allergy and Immunology clinic in Toronto, Ontario between June 2000 and June 2021. Patients were included if they were treated with enoxaparin for severe refractory CSU. Included patients who responded to enoxaparin were phoned for follow-up.

**Results:** Ten patients were treated with a recommended daily dose of enoxaparin 30mg subcutaneous for their CSU. Four patients were excluded for lost to follow up (n = 1, 10%), early drug discontinuation to participate in a clinical trial (n = 2, 20%), or try CBD oil (n = 1, 10%). Therefore, six patients (57.8 ± 13.7yo; n = 4, 66.7% female) were included in this study. All patients had associated angioedema and elevated d-dimer (766–3961 ng/ml FEU). Prior failed treatments were antihistamines (n = 6, 100%), 300–600mg Omalizumab (n = 6, 100%), prednisone (n = 5, 83.3%), Montelukast (n = 3, 50.0%) and 400mg hydroxychloroquine (n = 2, 33.3%). Two patients did not respond to enoxaparin and are being temporarily managed with low-dose cyclosporine. One patient stopped treatment after a month as they saw no improvement in their symptoms and found the injections to be inconvenient. However, their hives are currently uncontrolled and they are contemplating a retrial of enoxaparin. The remaining patients were on enoxaparin for six months including one month of tapering and have been in remission for two (n = 2, 66.6%) or three (n = 1, 33.3%) years. All CSU medications have been discontinued.

**Conclusions:** Enoxaparin can lead to remission of refractory CSU in patients who have elevated d-dimer and have failed first, second, and third line treatments.

**References:**

1. Asero R, Tedeschi A, Cugno M. Heparin and tranexamic acid therapy may be effective in treatment-resistant chronic urticaria with elevated d-dimer: a pilot study. Int Arch Allergy Immunol. 2010; 152:384–389.
2. Kobric D, Zaidi S, Abbas KF, Sussman G. Treatment refractory chronic spontaneous urticaria patients achieves remission with low molecular weight heparin. J. Allergy Clin. Immunol. 2019; AB51

## Case Reports

### 68 Life-threatening anaphylaxis during venom immunotherapy: a case report

#### Zainab Ridha^1^, Martine Boivin^2^, Aubert Lavoie^2^, Remi Gagnon^2^, Jean-Philippe Drolet^2^

##### ^1^Faculty of Medicine, Université Laval, Quebec, QC, Canada, ^2^Division of Allergy and Clinical Immunology, Department of Medicine, Centre Hospitalier de l’Université Laval (CHUL), Quebec, QC, Canada

###### **Correspondance:** Zainab Ridha

*Allergy, Asthma & Clinical Immunology* 2022, **17(Suppl 1)**: 68

**Background:** Hymenoptera venom anaphylaxis occurs in about 3% of adult population. Risk of fatal anaphylaxis is higher in patients with systemic mastocytosis (SM). Diagnostic criteria are not always fulfilled according to WHO diagnostic SM criteria, but most patients harbour KITD816V mutation, which has diagnostic relevance. Venom immunotherapy (VIT) is the only disease-modifying therapy for insect sting anaphylaxis. We present a case of severe anaphylaxis induced by VIT in a patient with normal baseline serum tryptase.

**Case Presentation:** A 53-year-old male working as an outdoor operator with a medical history of anaphylaxis after Hymenoptera sting and during previous VIT attempt was referred to our clinic to reintroduce VIT, successfully conducted using ultrarush protocol. At first treatment dose, the patient experienced disseminated erythroderma, diaphoresis, and agitation. His symptoms then culminated in full-blown anaphylaxis: he developed fatigue, abdominal pain, bradypnea, and circulatory collapse. He became unconscious and exhibited convulsive movements. He was immediately treated with multiple intramuscular epinephrine shots, intravenous (IV) fluids, IV antihistamines, and IV hydrocortisone. He finally recovered promptly. Laboratory investigations revealed normal baseline serum tryptase level. A bone marrow biopsy showed morphologically normal mast cells without multifocal dense mast cell infiltrates. Molecular genetic testing confirmed bone marrow KITD816V mutation.

**Conclusions:** This case highlights that serum tryptase level should be measured in patients with history of life-threatening venom reaction. Bone marrow biopsy must be seriously considered in case of anaphylaxis after VIT despite normal tryptase level to exclude mastocytosis. Diagnostic criteria of SM were not fully met in our patient. However, we hypothesised he suffers from prediagnostic indolent systemic mastocytosis as these patients can lack mast cell cluster and exhibit a serum tryptase level <20 ng/mL. KITD816V mutation should be considered as pre-diagnostic for SM as it is detected in 80% of patients. Patients without full diagnostic criteria for SM could need adapted VIT protocol.

**Statement of Consent**

Written Informed Consent for this case report was obtained from the patient.

### 69 An unusual case of anaphylaxis to chlorhexidine

#### Sujen Saravanabavan^1^, Scott Cameron^2,3^, Raymond Mak^2^, Tiffany Wong^2^, Stephanie C. Erdle^2^

##### ^1^Department of Pediatrics, Faculty of Medicine, University of Ottawa, Ottawa, ON, Canada, ^2^Division of Allergy and Immunology, Department of Pediatrics, University of British Columbia, Vancouver, BC, Canada, ^3^Community Allergy Clinic, Victoria, BC, Canada

###### **Correspondance:** Sujen Saravanabavan

*Allergy, Asthma & Clinical Immunology* 2022, **17(Suppl 1)**: 69

**Background:** Chlorhexidine’s excellent antimicrobial properties have resulted in its increasing use in healthcare settings. Proportionally, there has been an increase in type I and IV hypersensitivity reactions, including anaphylactic events. Many of these reactions occur in the preoperative or periprocedural context. This increase in allergic events is thought to be secondary to the rising use of chlorhexidine, and subsequent increase in sensitization.

**Case Presentation:** An 11-year-old female with inflammatory bowel disease (IBD) developed two separate anaphylactic reactions during her monthly infliximab infusion. Initially she was referred for assessment of infliximab allergy. However, after careful history taking, it was noted that on both occasions, chlorhexidine was used as the antiseptic agent prior to IV insertion. Skin prick testing was positive to chlorhexidine at 14x8mm and negative for other possible causative agents. Although chlorhexidine had been used and tolerated during other infliximab infusions, our hypothesis was that on these two occasions the chlorhexidine did not fully dry before IV insertion, leading to iatrogenic injection of chlorhexidine and resulting anaphylaxis. To confirm this hypothesis, timed skin prick testing was performed at 5-minute intervals as the chlorhexidine dried, with gradual decreasing wheal size and no wheal development after 15 minutes of drying. She was advised to avoid use of all chlorhexidine products in the future.

**Conclusions:** This case illustrates the importance of considering chlorhexidine in cases of procedural anaphylaxis, even if there is a history of tolerance. This case highlights opportunities for primary prevention at an institutional level by using alternative cleaning products for procedures with low risk of infection. Our center is working on implementing policies to discontinue use of chlorhexidine in patients requiring repeat procedures.

**Statement of Consent**

Written Informed Consent for this case report was obtained from the patient.

### 70 A good catch! The de novo presentation of heated shellfish hypersensitivity in a previously shellfish tolerant adult

#### Ijaz Ogeer^1^, Saajida Hosein^1^, Vince Wu^1^, Wardha Wardha^1^, Jason Ohayon^1,2^

##### ^1^Hamilton Allergy, Hamilton, ON, Canada, ^2^McMaster University, Hamilton, ON, Canada

###### **Correspondance:** Ijaz Ogeer

*Allergy, Asthma & Clinical Immunology* 2022, **17(Suppl 1)**: 70

**Background:** Worldwide prevalence of shellfish allergy (SA) is on the rise and estimated at 3%. Higher incidence has been reported in adolescents/adults. [1] Tropomyosin is the major allergen in shrimps and prawns, and has been characterized as heat-stable. Heated shrimp (HS) is shown to have an elevated tropomyosin concentration as compared to unheated shrimp (US).[2] Additionally, the extraction efficiency of tropomyosin is increased by heating.[2] Despite concerning histories for SA, standard skin prick testing (SPT) is not supportive of allergy diagnosis.

**Case Presentation:** A 57-year-old female previously tolerant of shellfish developed an acute adverse reaction with palmar/plantar pruritus minutes after consuming HS. She denied dyspnea and gastrointestinal symptoms. She responded to repeated doses of oral antihistamines. Her comorbidities included hypertension, diabetes and dyslipidemia controlled without change in medication. The patient was tolerant to finned fish.

SPT identified negative results to common food allergens including six shellfish extracts. Positive responses were noted to ragweed and mold spores only. Prick to prick testing (PPT) to HS and US was positive for HS alone. The patient was counselled on shellfish avoidance and prescribed emergency injectable epinephrine.

**Conclusions:** Current diagnostic algorithms require detailed history and extract-based SPT or serum specific IgE [1] for diagnosis of SA. Sensitivity of current commercial shellfish extracts is not as high as other foods, likely due to the highly variable protein content in extracts [3]. Alternatively, heated shellfish protein may be rendered allergenic, thus supporting the use of HS and US material for PPT.

De novo SA in previously tolerant patients can present with a negative SPT. Clinical acumen with PPT to heated and unheated samples is needed to catch this subset of patients, with the consideration of a diagnostic oral challenge. Further research is required to standardize the use of PPT in allergy testing.

**Statement of Consent**

Written Informed Consent for this case report was obtained from the patient.

### 71 A case series of three patients with both complete deletion of exon 8 of the c-kit gene and hereditary alpha-tryptasemia

#### Maggie M. Jiang, Peter Vadas

##### Division of Allergy and Clinical Immunology, St. Michael’s Hospital, University of Toronto, Toronto, ON, Canada

###### **Correspondance:** Maggie M. Jiang

*Allergy, Asthma & Clinical Immunology* 2022, **17(Suppl 1)**: 71

**Background:** Mutation of the c-kit proto-oncogene is strongly implicated in mast cell disorders[1]. Over 90% of adult patients with systemic mastocytosis (SM) have a gain of function point mutation (D816V) in exon 17 of c-kit[2]. C-kit mutations have also been detected in exons 8–11 in patients with SM[2]. Hereditary alpha-tryptasemia (HαT) can present with symptoms of mast cell activation (MCA) resembling those in SM, and has been found to occur in 5% of the general population and in 12–17% of patients with SM[3].

**Case Presentation:** Herein, we describe three patients with complete deletion of exon 8 of the c-kit gene. All three patients had symptoms of MCA along with elevated basal serum tryptase. Interestingly, two of the patients were first degree relatives with a strong family history of elevated tryptase and symptoms of MCA. All underwent bone marrow biopsy and additional cutaneous and/or gastrointestinal tract biopsies. None fulfilled WHO criteria for SM. Molecular analysis of the c-kit gene demonstrated that all three patients had complete deletion of exon 8 but no known pathogenic mutation, including the D816V mutation. TPSAB1 copy number analysis was consistent with a diagnosis of HαT in all patients.

**Conclusions:** This is the first description of complete deletion of exon 8 of the c-kit gene. Whether there is a strong association between this mutation and the presence of HαT or whether the patients underwent molecular analysis of the c-kit gene because they presented with symptoms of MCA with the incidental finding of this gene deletion is not yet known. Nevertheless, these three cases illustrate the complex work up of patients presenting with symptoms of MCA and highlight the importance of considering more common conditions such as HαT as causes of MCA over poorly understood c-kit mutations.

**Patient Consent**

Written informed consent for this case report was obtained from the patients.

### 72 “Don’t taste the rainbow”: rare presentation of anaphylaxis and contact urticaria post food colourant exposure.

#### Meridith Dales^1,2^, Jason Ohayon^2^, Wardha Wardha^2^

##### ^1^University of Waterloo, Waterloo, ON, Canada, ^2^Hamilton Allergy, Hamilton, ON, Canada

###### **Correspondance:** Meridith Dales

*Allergy, Asthma & Clinical Immunology* 2022, **17(Suppl 1)**: 72

**Background:** Food colourants play a large role in food production. Allura Red, Brilliant Blue, Tartrazine, and Sunset Yellow are the most common colour additives, with documented hypersensitivity reactions [1,2]. True anaphylactic reactions to food colourants are rarely reported, especially in adults. Skin prick testing rarely results in positive responses to food colourants [1].

**Case Presentation:** A 33-year-old female with a history of seasonal allergies, asthma, and anaphylaxis to banana presented to an allergy clinic for assessment of anaphylactic reactions post food colourant exposures. As a child, the patient developed throat tightness after consuming candy with red dye. Subsequent ingestion of yellow food dye in a throat lozenge resulted in throat tightness and difficulty breathing. On both occasions, the patient required epinephrine rescue with resultant allergic diagnosis to red and yellow colourants. A third reaction involved use of a bathing product (Bath Bomb) containing blue dye. The patient developed contact urticaria responsive to antihistamine treatment.

Skin prick testing was performed using the ClubHouse™ Food Colour Preparation vials in red, yellow and blue, of which an ingredient is tartrazine. Positive responses compared to histamine control (4 mm) were found in blue dye (10 mm), red dye (7 mm) and yellow dye (13 mm). The patient was confirmed to have multiple dye allergies. Counselling was provided on avoidance of food colourings, both via ingestion and contact, with provision of epinephrine auto-injector.

**Conclusions:** This study illustrates the rare presentation of colourant induced anaphylaxis by ingestion and urticaria by contact to red, yellow, and blue dyes. This case highlights the importance of medical history and allergy testing to relevant allergens in food induced anaphylaxis, along with the unique presentation of contact urticaria. Although extremely rare, allergists should be aware of food colourant anaphylaxis to assess and counsel accordingly.

**Statement of Consent**

Written Informed Consent for this case report was obtained from the patient.

### 73 When it’s not allergy- myasthenia gravis masquerading as COVID-19 vaccine allergy

#### Jonathan Nichilo^1^, Vince Wu^1^, Jason Ohayon^1^

##### ^1^Hamilton Allergy, Hamilton, ON, Canada, ^2^McMaster University, Hamilton, ON, Canada

###### **Correspondance:** Jonathan Nichilo

*Allergy, Asthma & Clinical Immunology *2022, **17(Suppl 1)**: 73

**Background:** Individuals with myasthenia gravis (MG) are of particular importance with regards to COVID-19 vulnerability and vaccination precedence (1). However, evidence suggests COVID vaccines may contribute to exacerbations within these populations (2). It is important to consider pre existing conditions in MG patients who have expressed subsequent symptoms to COVID-19 vaccinations, even after negative allergic testing.

**Case Presentation:** A 65 year old female with corticosteroid dependent MG was referred for allergy assessment to COVID vaccine components. The patient presented with lightheadedness, numbness/blisters in mouth, difficulty swallowing, chest pain, high pulse rate (96bpm), and low blood pressure (112/72) after receiving her first dose (D1) of the Pfizer vaccine. The patient tested negative to the main allergic components, polyethylene glycol (PEG) and polysorbate 80, but displayed symptoms of dizziness and respiratory distress after skin testing (ST). Physical examination was reassuringly normal, including vitals and respiratory examination. A vasovagal episode and anxiety was felt to be the cause of patients’ reactions. The patient was encouraged to continue with her second vaccine dose (D2), and advised antihistamine usage the day before, day of, and day after for reassurance purposes.

Further consultation with a neuromuscular specialist recommended corticosteroid augmentation 48 hours prior to one week post D2 to prevent MG exacerbation. The patient was advised to increase prednisone dosage (6mg to 8mg) two days prior to D2, and continue with 8mg for 1 week following. No adverse outcomes were reported following administration of D2. It was suspected the patient’s D1 triggered her myasthenia, presenting with allergy-like symptoms.

**Conclusions:** Negative allergic testing is insufficient to dismiss patients with preexisting MG. It is imperative to recognize underlying health conditions which may manifest adverse COVID-19 vaccine complications. In certain autoimmune conditions, such as MG, modest corticosteroid augmentation will prevent symptom presentation mimicking allergy from COVID vaccination.

**Statement of Consent**

Written Informed Consent for this case report was obtained from the patient.

### 74 Heterozygous BACH2 variant in a patient with history of immunodeficiency, recurrent angioedema and systemic lupus erythematosus

#### Peter Stepaniuk, Amin Kanani

##### University of British Columbia, Vancouver, BC, Canada

###### **Correspondance:** Peter Stepaniuk

*Allergy, Asthma & Clinical Immunology* 2022, **17(Suppl 1)**: 74

**Background:** *BACH2* is a transcription factor that serves as a critical regulator of T and B lymphocyte differentiation and maturation. Two missense heterozygous *BACH2* variants previously reported in the literature were associated with autoimmunity as well as a CVID-like picture. *BACH2*-deficient mice have also been shown to have absent Treg cells and an excess of effector and memory T cells resulting in autoimmunity. However, the true pathogenicity of these variants in humans is uncertain as there are 16 heterozygotes and one homozygote variant of *BACH2* in the gnomAD database.

**Case Presentation:** We present the case of a 54-year-old female with a longstanding history of recurrent pyelonephritis/urosepsis, systemic lupus erythematosus and angioedema since childhood. She has been on intravenous immunoglobulin replacement (IVIG) and C1 inhibitor replacement for many decades which has reduced her infectious and angioedema burden. Although pre-treatment investigations are not available, while on IVIG and C1 inhibitor replacement she was observed to have normal lymphocyte subsets, normal immunoglobulins (IgA, IgM, IgE), normal CH50/AH50, but a persistently low C4 at 0.15 g/L (0.20–0.60). Genetic testing identified a heterozygous variant *BACH2 *c.2362G > A, p.(Glu778Lys). The patient also had heterozygous variants in *CD55* and *NCF4* which were not felt to be pathogenic. The same heterozygous *BACH2* variant identified had previously been reported in a father and daughter both of whom presented with bowel inflammation and hypogammaglobulinemia presenting as recurrent sinopulmonary infections.

**Conclusions:** *BACH2* haploinsufficiency is increasingly being recognized as a possible cause of a syndrome of BACH2-related immunodeficiency and autoimmunity (BRIDA). Our patient has a long-standing history of immune dysregulation since childhood and it is possible that the identified *BACH2* variant is pathogenic. With increased access to genetic testing, it is likely that more *BACH2* variants will be identified in the future which will hopefully clarify the pathogenicity of this gene.

**Statement of Consent**

Written Informed Consent for this case report was obtained from the patient.

### 75 Choanal atresia masquerading as unilateral rhinitis

#### Aisha Mohammed, Savannah Sommerhalder, David Lindsay

##### University of Texas Medical Branch, Galveston, TX, USA

###### **Correspondance:** Aisha Mohammed

*Allergy, Asthma & Clinical Immunology* 2022, **17(Suppl 1)**: 75

**Background:** Neonatal nasal anomalies are rare. These anomalies include choanal atresia, congenital nasal pyriform aperture stenosis (CNPAS), nasolacrimal duct cysts, and congenital nasal septal deviation. Choanal atresia is often detected in the neonate with respiratory distress at birth requiring lifesaving airway placement, but when obstruction is unilateral, cases may go undetected until later in life.

**Case Presentation:** A 3 year old female, ex 30 weeker triplet, is referred to the Allergy and Immunology clinic by her pediatrician for persistent nasal congestion, right sided rhinorrhea, difficulty feeding and intermittent right sided purulent discharge that started shortly after birth and persisted despite oral antihistamines, nasal sprays and recurrent antibiotics. She had multiple visits with ENT for lacrimal duct stenosis, otitis media, bacterial conjunctivitis & sinusitis. Subsequent rhinoscopy revealed right sided blockage. Landmark sinus CT confirmed choanal atresia. This was surgically repaired with complete relief of symptoms. Of note, due to prematurity, in the NICU she required a feeding support via NGT that, coincidentally, was repeatedly placed in the patent left nostril.

**Conclusions:** Bilateral choanal atresia is detected at birth due to life-threatening respiratory distress and requires lifesaving airway placement. However, when atresia is unilateral, it may remain undetected until later in life, such as in our 3 year old patient. During her NICU stay, nasogastric tubes for feeding were fortuitously placed in the patent left nostril each time, thereby evading the diagnosis of right-sided choanal atresia. Our patient had unilateral choanal atresia masquerading as chronic rhinitis. Choanal atresia is a rare congenital deformity resulting from failed recanalization of nasal fossae during fetal development. In 50% cases Choanal atresia is an isolated finding; but is a cardinal feature of CHARGE syndrome. Suspicion for congenital and non-congenital airway obstruction should be high in patients who do not improve with nasal steroids and antihistamines prompting further evaluation.

**Statement of Consent**

Written Informed Consent for this case report was obtained from the patient.

### 76 New onset chronic urticaria following AstraZeneca COVID-19 vaccination

#### Arun Dhir^1^, Godfrey Lam^2^

##### ^1^Internal Medicine, Department of Medicine, University of British Columbia, Vancouver, BC, Canada, ^2^Allergy and Immunology, Department of Medicine, University of British Columbia, Vancouver, BC, Canada

###### **Correspondance:** Arun Dhir

**Allergy, Asthma & Clinical Immunology** 2022, **17(Suppl 1)**: 76

**Background:** Vaccinations have been suggested to be a possible trigger for chronic urticaria. While acute urticaria following COVID-19 vaccination has been described in the literature, as of this writing there are no published cases of new onset chronic urticaria following vaccination.

**Case Presentation:** A 55-year-old male who was previously healthy, with no history of atopy or urticaria, received his first dose of the AstraZeneca vaccine. He had no immediate symptoms following vaccination. By the next morning, he developed inducible urticaria with dermatographism. He had no fevers, chills, lymphadenopathy, or arthralgias. He was started on regular non-sedating antihistamines, with some effect. He had a normal complete blood count and differential. Tryptase, C3, and C4 levels, and total complement activity were normal. He was assessed by allergy and immunology approximately 8 weeks post-vaccination and continued to have ongoing inducible urticaria. Skin prick testing to 1:1 polyethylene glycol 3350 (Restorolax) and 1:1 polysorbate 80 (Refresh Ultra eye drops) were negative. He was advised to receive an mRNA-based COVID-19 vaccine for his second dose.

**Conclusions:** It is possible that the development of chronic urticaria in this patient may have been due to the use of an adenoviral vector-based vaccine, given the association between viral infections and chronic urticaria. Clinicians should be cognisant that chronic urticaria may present following COVID-19 vaccination. As it is likely that similar presentations will be reported in the future, it is crucial that patients understand the non-allergic nature of chronic urticaria and that they are not at an elevated risk of an IgE-mediated allergic reaction following a second vaccination. Given that chronic forms of urticaria tend to be benign and may be self-limited, patients should be provided with the opportunity to consider the risks and benefits of a second dose.

**Statement of Consent**

Written Informed Consent for this case report was obtained from the patient.

### 77 Absent antibody response following Pfizer COVID-19 vaccination in a patient on ocrelizumab with pre-existing hypogammaglobulinemia and heterozygous variant in TNFRSF13B

#### Arun Dhir^1^, Amin Kanani^2^

##### ^1^Internal Medicine, Department of Medicine, University of British Columbia, Vancouver, BC, Canada, ^2^Allergy and Immunology, Department of Medicine, University of British Columbia, Vancouver, BC, Canada

###### Correpondance: Arun Dhir

*Allergy, Asthma & Clinical Immunology* 2022, **17(Suppl 1)**: 77

**Background:** Ocrelizumab is a form of anti-CD20 therapy with B cell depleting effects used in patients with multiple sclerosis (MS). This medication has been associated with an impaired antibody response to mRNA COVID-19 vaccinations. Amongst MS patients, receiving B cell depleting therapy has been identified as a risk factor for severe COVID-19 infection.

**Case Presentation:** A 56-year-old physician with a history of MS and resolved chronic spontaneous urticaria was incidentally found to have hypogammaglobulinemia, with IgG levels ranging from 4.0 to 4.8 (normal range: 7.0–16.0). He had normal IgA and IgM levels. There was no history of recurrent sinopulmonary infections. Medications included aspirin and glatiramer initially, but he was later switched to ocrelizumab due to progression of his MS. He had protective measles, rubella, and varicella antibody titres. Initially, he had inadequate titres to mumps but after vaccination, he achieved protective levels. However, response to pneumococcal vaccination administered while on ocrelizumab was inadequate. His last dose of ocrelizumab was in October of 2020. He received his first dose of the Pfizer COVID-19 mRNA in January of 2021 and his second dose 41 days later. At 55 days post-vaccination, he had no evidence of COVID-19 antibody titres, measured by two different assays. In light of his hypogammaglobulinemia and poor vaccine responses, genetic analysis was performed. A pathogenic heterozygous variant in TNFRSF13B was identified, which is associated with autosomal recessive common variable immunodeficiency. Possession of a single allele may have contributed to the patient’s presentation.

**Conclusions:** Clinicians should be aware that patients on ocrelizumab may have an inadequate response to COVID-19 vaccinations and that these patients are also at a higher risk of severe COVID-19 infection. Consideration should be given to attempting vaccination prior to initiating therapy if possible, or otherwise vaccinating near the end of dose cycles.

**Statement of Consent**

Written Informed Consent for this case report was obtained from the patient.

### 78 A case of cocoa allergy in a young girl

#### Wade T. Watson^1^, Edson Castilho^2^

##### ^1^Dalhousie University, IWK Health Centre, Halifax, NS, Canada, ^2^IWK Health Centre, Halifax, NS, Canada

###### **Correspondance:** Wade T. Watson

*Allergy, Asthma & Clinical Immunology* 2022, **17(Suppl 1)**: 78

**Background:** Chocolate is produced by fermenting, roasting and grinding cacao beans from the fruit pod of the cacao tree. Cocoa powder is made by removing 75% of the cocoa butter and drying the residue. Cocoa powder contains carbohydrates, fat and protein. Allergic reactions to cocoa are extremely rare. We report a case of an allergic reaction to cocoa in a young girl.

**Case Presentation:** A six year old female with multiple food allergies (kiwi, tree nuts, wheat and egg) ingested a drink consisting of one teaspoon of Hershey’s™ Cocoa mixed with boiled milk and sugar. After two to three sips she complained of abdominal pain and she developed facial urticaria. There were no other symptoms. She was given an oral antihistamine and her symptom improved within two hours. Although an epinephrine auto injector was available, it was not used. An epicutaneous test to the Hershey’s Cocoa resulted in a 9 mm wheal. The reaction was reported to Canadian Food Inspection Agency. The cocoa powder was tested for contamination and found to be free of wheat, egg and tree nuts. There was no test available for kiwi. An epicutaneous test to a different brand of cocoa powder (Camino™) was subsequently performed and was also positive with a 9 mm wheal. Epicutaneous tests to both cocoa powders were negative in a control subject.

**Conclusions:** Chocolate/cocoa can be a food allergen. While many allergic reactions to chocolate have been attributed to contamination with other food allergens, we believe that this demonstrates a true cocoa allergy. Isolated chocolate/cocoa allergy should be considered in those individuals reacting to chocolate/cocoa-containing food products.

**Statement of Consent**

Written Informed Consent for this case report was obtained from the patient.

### 79 The use of dupilumab in severe atopic dermatitis during pregnancy

#### Nabeel Akhtar^1^, Touraj Khosravi-Hafsehjani^2^, Daud Akhtar^4^, Gurbir Dhadwal^2^, Amin Kanani^3^

##### ^1^University College Dublin, School of Medicine, Dublin, Ireland, ^2^University of British Columbia, Department of Dermatology and Skin Science, Vancouver, BC, Canada, ^3^University of British Columbia, Department of Medicine, Division of Allergy and Immunology, Vancouver, BC, Canada, ^4^University of British Columbia, Vancouver, BC, Canada

###### **Correspondance:** Nabeel Akhtar

*Allergy, Asthma & Clinical Immunology* 2022, **17(Suppl 1)**: 79

**Background:** Atopic dermatitis (AD), is a chronic inflammatory skin disorder that manifests with severe pruritis. In pregnancy, AD is the most common skin disease accounting for 36–59% of all dermatoses. The rising prevalence of AD poses a significant economic and health burden. Current treatment options for moderate to severe AD in pregnancy include oral corticosteroids, azathioprine, cyclosporine and phototherapy. Presently, the only biologic approved for moderate to severe AD is dupilumab. We describe a case of severe AD treated safely with dupilumab during pregnancy with subsequent resolution of symptoms in the post-partum period.

**Case Presentation:** A 33-year-old, presented in the setting of worsening AD refractory to high potency topical corticosteroids, systemic corticosteroids, antibiotics, methotrexate, cyclosporine as well as ultraviolet phototherapy. Her Investigator Global Assessment for Atopic Dermatitis score of 4 was indicative of severe disease. She was subsequently initiated on dupilumab 300mg every 2 weeks with significant improvement in her condition. She became pregnant 12 months later and remained on dupilumab 300mg every 2 weeks until 27 weeks gestation at which point dupilumab was stopped. Unfortunately, she experienced a severe flare of AD only 2 weeks after discontinuation. Therefore, she was restarted on dupilumab at 29 weeks gestation. At 38 weeks the patient underwent urgent Cesarean section with delivery of a healthy female infant There were no issues postpartum. The patient was reassessed 6 weeks postpartum and had opted to breastfeed. Her AD remained well controlled without recommencement of her dupilumab therapy.

**Conclusions:** This case report adds to current literature regarding the use dupilumab in pregnancy. To our knowledge, this is the first case report of a pregnant patient with AD treated with dupilumab in Canada. Our case is the first to demonstrate symptom resolution without re-initiation of dupilumab in the postpartum setting with excellent maternal and fetal outcomes.

**Statement of Consent**

Written Informed Consent for this case report was obtained from the patient.

### 80 Common variable immunodeficiency complicated by autoimmune cytopenias and non-malignant multisystem lymphoid proliferations

#### Leila A. Alenazy^1^, Geneviève C. Digby^2^, Stephen Betschel^3^, Rozita Borici-Mazi^4^

##### ^1^Department of Medicine, Queen’s University, Kingston, ON, Canada, ^2^Division of Respirology, Department of Medicine, Queen’s University, Kingston, ON, Canada, ^3^Division of Clinical Immunology and Allergy, Department of Medicine, University of Toronto,, Toronto, ON, Canada, ^4^Division of Allergy and Immunology, Department of Medicine, Queen’s University, Kingston, ON, Canada

###### **Correspondance:** Leila A. Alenazy

*Allergy, Asthma & Clinical Immunology* 2022, **17(Suppl 1)**: 80

**Background:** Common variable immune deficiency (CVID) is a primary immunodeficiency characterized by decreased or absent antibody production and impaired humoral immune function. Although autoimmune cytopenias are reported in 11–18% of CVID patients, lymphoid proliferations leading to multi-organ dysfunction are less well characterized.

**Case Presentation:** We describe a 51 y male who was diagnosed with CVID in 2017 and started on immunoglobulin replacement. A year later, he developed mediastinal and hilar lymphadenopathy due to non-specific granulomatous inflammation. He subsequently developed sudden onset of elevated polyclonal IgG and IgM, severe life-threatening autoimmune cytopenias, diffuse lymphadenopathy and massive splenomegaly. Several lymph node biopsies and bone marrow examinations ruled out infectious or malignant causes. Genetic sequencing using a broad panel for primary immunodeficiency (including FAS, FASL, CASP8) was unremarkable. His cytopenias and elevated immunoglobulins responded to treatment with rituximab consisting of 375mg/m^2^ once weekly for 4 doses. His symptoms relapsed 6 months later, with severe cytopenias, pulmonary opacities, and diffuse lymphadenopathy. A second course of rituximab was administered, followed by splenectomy, and his cytopenias recovered. Several months later, he was admitted with shortness of breath, severe hypoxemia requiring a FiO2 80%, diffuse pulmonary infiltrates, lymphadenopathy and elevated immunoglobulins. His lung condition responded to treatment with high dose steroids and rituximab. Although lung biopsy was not performed due to the severity of hypoxemia, the clinical presentation, imaging and response to treatment were suggestive of granulomatous–lymphocytic interstitial lung disease (GLILD). A liver biopsy was remarkable for significant lymphocytic infiltrates. An inguinal lymph node biopsy showed follicular lymphoid hyperplasia and no malignancy. After discharge from hospital, he remains stable on mycophenolate, rituximab every 6 months and immunoglobulin replacement.

**Conclusions:** This case report underscores the significant morbidity caused by non-malignant lymphoid prolifera-tion in CVID patients, the necessity to monitor for malignant transformation, and the complexities of management.

**Statement of Consent**

Written Informed Consent for this case report was obtained from the patient.

### 81 Telangiectasia macularis eruptiva perstans with systemic involvement: a case report

#### Abdul Rahman Ayoub^1^, Jonathan Lai^2^, Kara Robertson^3^

##### ^1^Department of Medicine, Western University, London, ON, Canada, ^2^Division of Histopathology, Life Labs, Toronto, ON, Canada, ^3^Division of Clinical Immunology and Allergy, Department of Medicine, London, ON, Canada

###### **Correspondance:** Abdul Rahman Ayoub

*Allergy, Asthma & Clinical Immunology* 2022, **17(Suppl 1)**: 81

**Background:** Telangiectasia macularis eruptiva perstans (TMEP) is a rare form of cutaneous mastocytosis which usually presents as ill-defined reddish brown telangiectactic macules [1]. Although cutaneous mastocytosis rarely presents with systemic involvement, almost half of patients with TMEP demonstrate systemic mastocytosis [2]. Currently, there is no guidance surrounding systemic testing in patients with TMEP.

**Case Presentation:** A 50-year-old male was referred to our Clinical Immunology and Allergy clinic with a history of a rash. He initially presented to hospital 12 years prior with group A beta hemolytic streptococcus bacteremia treated with multiple different antibiotics. One week following discharge, he developed erythematous brown spots on his right leg which were flat, non-pruritic and not painful. The rash later expanded to his trunk and extremities. A skin biopsy performed two years prior to referral to our clinic demonstrated telangiectasia macularis eruptiva perstans. The CD117 stain showed increased perivascular and interstitial mast cells in the superficial dermis. Darier’s sign was negative on physical examination. Although he had no symptoms of systemic involvement, a serum tryptase was elevated at 47.6 ng/mL in the context of normal kidney and liver function. A skeletal survey was normal and an abdominal ultrasound ruled-out splenomegaly. The patient was referred to Hematology, and a myeloid panel was negative for the c-KIT mutation. Bone marrow biopsy demonstrated a mild increase in paratrabecular and perivascular atypical mast cells, in keeping with systemic mastocytosis. The patient will return to the Allergy Clinic for venom testing.

**Conclusions:** Unlike other forms of cutaneous mastocytosis, patients with TMEP have a high likelihood of an underlying systemic mast cell disorder. Therefore, any patient presenting with characteristic skin findings should be investigated as having a cutaneous manifestation of systemic mastocytosis. This case demonstrates the utility of serum tryptase, and its role in triggering additional investigations and guiding appropriate therapy.

**Statement of Consent**

Written Informed Consent for this case report was obtained from the patient.

### 82 A case of selective radiocontrast media reaction with confirmation and clinical guidance using intradermal skin testing

#### Shun Chi Ryan Lo, Persia Pourshahnazari

##### University of British Columbia, Vancouver, BC, Canada

###### **Correspondance:** Shun Chi Ryan Lo

*Allergy, Asthma & Clinical Immunology* 2022, **17(Suppl 1)**: 82

**Background:** Immediate reactions to radiocontrast media (RCM) have traditionally been attributed to non-IgE mediated direct mast cell activation. Previous studies have demonstrated positive intradermal testing in some patients, suggesting an underlying IgE-mediated mechanism; however, reported sensitivity has ranged widely, and clinical use of skin testing remains controversial. Recently published expert consensus recommendations include consideration of skin testing for identification of alternative RCM. Here, we report a case in which skin testing was vital to a favourable outcome.

**Case Presentation:** Written consent for publication was obtained from the following patient’s next of kin. A 67 year-old man developed 2 episodes of reactions attributed to RCM during coronary angiograms. Risk factors included only severe cardiovascular disease, without background atopy. In the first episode, pruritus, flushing, voice hoarseness, urticaria and angioedema developed minutes after administration of an unknown RCM, necessitating epinephrine and abortion of the angiogram. Angiogram was reattempted 5 days later, with prednisione, hydrocortisone and diphenhydramine premedication. The same symptoms developed within minutes of receiving ioversol. Intraoperative change of RCM to iodixanol did not resolve symptoms. Intradermal testing to iohexol, iodixanol and iopamidol (1:10 and 1:100 dilutions) performed 10 months later was positive to iohexol and iodixanol, and negative to iopamidol at both dilutions. Ioversol was not available for testing. Subsequent angiogram using iopamidol proceeded without reaction.

**Conclusions:** In this case, the results of skin testing provided support of IgE-mediated reactions to RCM. It further successfully identified an alternative RCM to provide clinical guidance. These findings reinforce that skin testing should be considered for evaluation of immediate reactions to RCM.

**Statement of Consent**

Written Informed Consent for this case report was obtained from the patient.

### 83 HAE-nC1 INH with reduction in C1 INH during an acute attack in one sibling

#### Mariam Narous^1^, Chrystyna Kalicinsky^1,2^

##### ^1^Department of Internal Medicine, University of Manitoba, Winnipeg, MB, Canada, ^2^Department of Allergy and Clinical Immunology, University of Manitoba, Winnipeg, MB, Canada

###### **Correspondance:** Mariam Narous

*Allergy, Asthma & Clinical Immunology* 2022, **17(Suppl 1)**: 83

**Background:** Hereditary angioedema with normal C1 esterase inhibitor level and function (HAE-nC1 INH) is a familial form of bradykinin-mediated angioedema, without urticaria. Attacks can be prolonged, involving laryngeal, mucosal, limb and gastrointestinal edema. We present a case of two siblings with typical HAE symptoms, though inconsistent C1 esterase inhibitor biochemistries.

**Case Presentation:** Sibling 1: 60-year-old female with frequent episodes of abdominal distention, lip and limb swelling, raspy breathing, occurring since menarche. Baseline C1 esterase inhibitor level, function, and C4 normal. During one episode, C1 esterase inhibitor level decreased to 0.16 g/L with normal C4. Failing high-dose antihistamine, she was trialed on prophylactic C1 esterase inhibitor, Berinert, 1500–2000 units IV almost daily, and Icabitant for acute attacks. With prophylaxis, she had frequent, but less severe attacks. Response to Icatibant incomplete.

Sibling 2: 57-year-old female, with severe abdominal swelling since menarche. Hysterectomy for adenomyosis at the age of 31 did not reduce abdominal attacks. Non-abdominal swelling began at 35 years, with laryngeal swelling while running a marathon. Normal C1 inhibitor level, function and C4 at baseline. C1 inhibitor level did not decrease with attacks. She was trialed on prophylactic C1 esterase inhibitor, Berinert 1500–2000 units IV, escalating to daily use, and Icabitant for acute attacks, with incomplete response.

To confirm this was HAE-nC1 INH, rather than atypical HAE Type 1, sequencing of SERPING1 and targeted mutation analysis for F12 (codon 328 and c.971_1018+24del72) and PLG (p.K330E) was performed, demonstrating no abnormalities. Screening of the ANGPT1 gene was not available.

**Conclusions:** Genetic testing confirmed both sisters have HAE-nC1 INH of unknown etiology. The finding of reduction in C1 inhibitor level during an attack, and normal at baseline, has been documented in our centre with idiopathic bradykinin-mediated angioedema, but not in HAE. This may represent a specific form of HAE-nC1 INH.

**Statement of Consent**

Written Informed Consent for this case report was obtained from the patient.

### 84 A case of sulphite sensitivity presenting as status asthmaticus

#### Andrew Wong-Pack, Nina Jindal

##### University of Toronto, Toronto, ON, Canada

###### **Correspondance:** Andrew Wong-Pack

*Allergy, Asthma & Clinical Immunology* 2022, **17(Suppl 1)**: 84

**Background:** Sulphite sensitivity is a described trigger for bronchospasm in 5% of asthmatics [1], thought to be secondary to sulphur dioxide generation in the oropharynx. High levels are commonly found in wine [1].

**Case Presentation:** A 41 year old male with no significant comorbidities ingested a meal containing tilapia and fruits, while visiting a local vineyard during late summer. Approximately 1 hour later he experienced shortness of breath and felt panicked. Symptoms rapidly progressed to respiratory arrest and he was found to be cyanotic and unresponsive. EMS was called and CPR was performed. He was transferred to hospital, placed on ventilatory support and treated with systemic corticosteroids. He was discharged subsequently after negative cardiac workup.

On further history, he had been consuming wine prior to onset of symptoms. He previously only used PRN Ventolin for periodic dyspnea but had no formal diagnosis of asthma. He also endorsed worsening of asthma symptoms during pollen seasons. He had noticed symptoms worsening with wine consumption in the past and previously mostly avoided this.

He had negative skin prick testing to all relevant foods and tolerated all same foods without issue thereafter. He had positive skin testing to grass and ragweed. Baseline serum tryptase was unremarkable. PFTs showed moderate, fully reversible airflow obstruction, with significant bronchodilator response, consistent with asthma. He was prescribed Symbicort for maintenance and reliever therapy and provided with a list of sulphite rich foods to avoid.

**Conclusions:** Status asthmaticus and sulphite sensitivity should be considered in possible anaphylaxis [2] and are important considerations for allergists. Sulphites are uncommon but important potential triggers for asthmatic patients [2].

This is a unique presentation of status asthmaticus exacerbated by sulphite sensitivity with exposure to sulphites, concurrent aeroallergen exposure during peak pollen seasons in a setting of previously unrecognized allergic asthma.

**Statement of Consent**

Written Informed Consent for this case report was obtained from the patient.

### 85 “By golly, Jim, I’m a doctor not a pharmacist”: The allergist role in selecting the appropriate calcium supplement to prevent anaphylaxis in the shellfish allergic population

#### Tyler Seto, Wardha Wardha, Jason Ohayon

##### Hamilton Allergy, Hamilton, ON, Canada

###### **Correspondance:** Tyler Seto

*Allergy, Asthma & Clinical Immunology* 2022, **17(Suppl 1)**: 85

**Background:** Calcium is necessary to prevent disease in the adult population where nearly half is currently using calcium supplementation (1,2). Calcium carbonate (CaC), a common calcium supplement, is derived from shellfish (1). Shellfish allergy may pose a risk in patients taking this form of calcium supplementation. The importance of identifying alternative sources of calcium supplementation in this population is necessary for the consulting allergist to prevent anaphylaxis. Therefore, the appropriate calcium supplementation must be considered for shellfish allergic patients.

**Case Presentation:** A 53 year-old male with a suspected shellfish allergy was referred in 2008 for an allergy assessment. Shrimp ingestion led to an anaphylactic reaction characterized by nausea, cramps, dizziness, sweating, and diarrhea. Shellfish allergy was confirmed and epinephrine was prescribed with counselling and avoidance. In 2020, the patient was diagnosed with osteopenia and CaC supplementation was recommended. He then developed a similar anaphylactic reaction as above, but milder. CaC was suspected given its sourcing from shellfish. The patient was counselled to avoid CaC and an arrangement was made for an in-office oral challenge to an alternate source of calcium. The patient underwent an oral challenge to 300 mg of calcium citrate, a non-shellfish pharmaceutically derived source of calcium. The patient tolerated the challenge over a 3 hour monitoring period without any adverse effects.

**Conclusions:** This presentation reveals the importance of identifying patients at risk for anaphylaxis due to shellfish derived CaC. Aging adults with increasing prevalence of osteoporosis should be screened for shellfish allergy prior to recommendation of CaC. Allergists counselling these patients at younger ages should also highlight the importance of avoidance of CaC found easily in the over the counter sections of pharmacies. Pharmaceutically derived calcium citrate can act as an alternative calcium supplement for shellfish allergic patients.

**Statement of Consent**

Written Informed Consent for this case report was obtained from the patient.

### 86 Developing a tocilizumab desensitization protocol from scratch for a patient with Castleman’s disease: a case report

#### Angela S. Maccan^1^, Stephanie C. Erdle^1^, Donna-Marie Lynch^2,4^, Kathleen A. Marquis^2,4^, Tim Lau^3^, Raymond Mak^1^

##### ^1^University of British Columbia, Vancouver, BC, Canada, ^2^Brigham and Women’s Hospital, Boston, MA, USA, ^3^Vancouver General Hospital, Vancouver, BC, Canada, ^4^Harvard Medical School, Boston, MA, USA

###### **Correspondance:** Angela S. Maccan

*Allergy, Asthma & Clinical Immunology* 2022, **17(Suppl 1)**: 86

**Background:** Anaphylaxis to biologic agents may preclude patients from receiving appropriate therapy for their autoimmune disease. Drug desensitization can allow patients to continue to safely receive first line therapy but is resource intensive and tolerance is only temporary. Patients need to be re-desensitized with each infusion.

**Case Presentation:** We present the case of a 40 year old female with HHV-8 negative multicentric Castleman’s disease who was well-controlled on monthly tocilizumab infusions before developing severe anaphylaxis on her most recent dose. Intradermal skin testing was strongly positive to tocilizumab at 1:100, 1:10 and neat concentrations.

As the patient required tocilizumab for the treatment of her Castleman disease, a custom 16-step desensitization protocol in the intensive care unit was suggested. On step 16, she experienced intense abdominal cramping requiring large doses of intravenous (IV) dilaudid. Given the severe side effects experienced, monthly desensitization may not be ideal. Our plan is to transition her to scheduled subcutaneous (SC) tocilizumab weekly after IV desensitization to maintain a more steady serum level. In order to facilitate this transition, our clinical pharmacists performed a detailed pharmacokinetic/ pharmacodynamic review of SC tocilizumab to ensure feasibility, optimal timing of doses and to review peak/ trough serum levels for safety. A 7-step subcutaneous desensitization protocol with weekly injections was suggested.

**Conclusions:** This is a unique case in which a patient developed an allergy to a longstanding medication, necessitating a customized approach to desensitization which is still being evaluated.Transitioning a patient to SC tocilizumab after initial IV desensitization may be a feasible option to minimize side effects and reduce resource utilization, however further investigations into the safety of this approach is necessary.

**Statement of Consent**

Written Informed Consent for this case report was obtained from the patient.

### 87 Management of alopecia areata with a second generation antihistamine: a case report

#### Angela S. Maccan, Stephanie C. Erdle, Wingfield Rehmus, Tiffany Wong

##### University of British Columbia, Vancouver, BC, Canada

###### **Correspondance:** Angela S. Maccan

*Allergy, Asthma & Clinical Immunology* 2022, **17(Suppl 1)**: 87

**Background:** Alopecia areata (AA) is an unpredictable autoimmune disease which can be difficult to treat. Common treatments for mild disease include corticosteroid injections (poorly tolerated by children) or topical therapies. Two case reports have described a response of alopecia areata to mono-therapy with antihistamines and one retrospective review found benefit for use of antihistamines as an adjunct to topical therapy. The potential mechanism of antihistamine in treatment of AA has not been well characterized. There are no published reports of pediatric AA patients being treated with antihistamines alone.

**Case Presentation:** We present the case of a five year old girl who developed alopecia totalis over the course of several months after one year of localized patches on her scalp.

She was seen by a pediatric dermatologist and treated with topical clobetasol daily, but this was discontinued when the patches consolidated into alopecia totalis. She added a multivitamin and homeopathic supplement with no effect. At the family’s request, she was seen by Allergy to consider allergic triggers for the alopecia, and to discuss treating with antihistamines. Serum specific IgE antibodies to common environmental allergens were negative. Given the safety profile of second generation antihistamines, it was decided to try fexofenadine 30mg daily, increasing to twice daily after three weeks of no improvement.

Within 2 weeks of commencing twice daily dosing, hair regrowth was noted.There have been no adverse events throughout the treatment. Our plan is to complete 6 months of twice daily fexofenadine and then slowly taper the dose.

**Conclusions:** This case suggests the potential utility of trialing second generation antihistamines in the treatment of alopecia areata. Further monitoring of this patient is required to assess disease control upon taper of the antihistamine. More studies are required to clarify the role of atopic immunology in alopecia areata.

**Statement of Consent**

Written Informed Consent for this case report was obtained from the patient.

### 88 Cholinergic urticaria: a possible genetic link? Monozygotic twins with cholinergic urticaria

#### Vaidehi Bhatt, Wardha Wardha, Jason Ohayon

##### Hamilton Allergy, Hamilton, ON, Canada

###### **Correspondance:** Vaidehi Bhatt

*Allergy, Asthma & Clinical Immunology* 2022, **17(Suppl 1)**: 88

**Background:** Cholinergic urticaria (CU) is characterized by pruritic wheals secondary to elevated core temperature. Genetic studies of chronic urticaria, urticaria pigmentosa, and non-histaminergic angioedema have established possible multiple gene polymorphisms but few have established a genetic link in cholinergic urticaria. Genetic susceptibility to cholinergic urticaria may overlap with genetic susceptibility to atopic disease which relationship is unknown in monozygotic twins.

**Case Presentation:** Monochorionic diamniotic twins, aged 13, presented to an allergy clinic with an 18-month history of pruritic rashes on the trunk and back post-exercise. Both twins experienced an onset of pinpoint urticarial lesions with cardiovascular exercise, limited to the skin without anaphylaxis. The urticaria resolved through treatment by non-sedating oral antihistamines or consistent lowering of core temperature. There was no history of any spontaneous urticarial lesions without increased temperature.

Allergy assessment included dermatologic examination, skin prick testing (SPT), and planned urticaria provocation testing. Both twins tested positive for grass and ragweed pollen allergy, as well as house dust mite allergy on SPT. Twin A also tested positive for dog dander allergy whereas Twin B tested positive for rabbit allergy. Urticaria provocation testing could not be completed during the initial encounter but is planned for the second encounter. Both twins were prescribed oral antihistamines and counseled for use 1 hour prior to exercise.

**Conclusions:** This case identifies a unique identical twin presentation of CU. The patients’ identical genetic disposition may play a role in the development of atopic presentation, with similar IgE-mediated inhalant presentation and unique cholinergic urticarial presentation. The overlap of genetic susceptibility in cholinergic urticaria and atopic disease should be further highlighted. The role of genetics in CU presentation should be studied in a larger capacity in monozygotic twins and siblings to understand disease etiology and to highlight the risk for CU in siblings.

**Statement of Consent**

Written Informed Consent for this case report was obtained from the patient.

### 89 Leucovorin hypersensitivity: a case report

#### Geetanjalee Sadi, Kara Robertson

##### Division of Clinical Immunology and Allergy, Department of Medicine, Western University, London, ON, Canada

###### **Correspondance:** Geetanjalee Sadi

*Allergy, Asthma & Clinical Immunology* 2022, **17(Suppl 1)**: 89

**Background:** Leucovorin is a reduced form of folic acid and is used to reduce the toxic side effects of methotrexate or enhance the activity of fluorouracil. Leucovorin has multiple clinical applications including rescue therapy following methotrexate and in combination with fluorouracil (5-FU) for the treatment of colon cancer. Few cases of leucovorin hypersensitivity reactions have been reported in the literature, and even fewer have been confirmed with objective testing.

**Case Presentation:** A 53 year old woman diagnosed with stage III colorectal cancer was treated with a right hemicolectomy and adjuvant chemotherapy with FOLFOX; a chemotherapy regimen consisting of oxaliplatin, leucovorin and 5-FU. She developed recurrent hives with multiple oxaliplatin infusions, despite premedication with dexamethasone and montelukast. Leucovorin was administered concurrently during these infusions. She completed a desensitization protocol to oxaliplatin administered by her medical oncologist, since this was assumed to be the culprit agent. Despite several months on desensitization protocol, she continued to develop hives. The patient was also found to have vomiting, hives and elevated blood pressure following infusion with leucovorin and 5-FU without oxaliplatin, but not during infusions of 5-FU alone. Leucovorin allergy was not initially suspected. Her chemotherapy regimen was changed to single-agent capecitabine; which has lower response rate. She was then referred for allergy assessment. On evaluation, skin prick and intradermal testing to leucovorin at a concentration of 10mg/mL was positive at dilutions of 1:10 and 1:100. Skin testing to oxaliplatin at a concentration of 5mg/mL was negative.

**Conclusions:** Hypersensitivity reactions during FOLFOX cycles are commonly attributed to oxaliplatin. Although leucovorin hypersensitivity is rare, it is important to recognize that IgE mediated reactions to leucovorin can occur and can be confirmed with objective testing.

**Statement of Consent**

Written Informed Consent for this case report was obtained from the patient.

